# Targeting the undruggable transcription factor, KLF5, with a peptidomimetic small molecule, NC114, attenuates pressure overload-induced cardiac remodeling and fibrosis

**DOI:** 10.1038/s41598-025-32155-y

**Published:** 2026-01-12

**Authors:** Thanachai Methatham, Natsuka Kimura, Shota Tomida, Tamaki Ishima, Yuki Taguchi, Hideki Uosaki, Eiji Sakashita, Hitoshi Endo, Ryozo Nagai, Kenichi Aizawa

**Affiliations:** 1 Department of Translational Research, Clinical Research Center, Jichi Medical University Hospital, Tochigi, 329-0498 Japan; 2 Division of Functional Biochemistry, Department of Biochemistry, Jichi Medical University, Tochigi, 329-0498 Japan; 3 Jichi Medical University, Tochigi, 329-0498 Japan; 4 Clinical Pharmacology Center, Jichi Medical University Hospital, Tochigi, 329-0498 Japan

**Keywords:** KLF5, Hypertrophy, Fibrosis, Pressure overload, Oxidative stress, Metabolic dysregulation, Biochemistry, Cardiology, Cell biology, Diseases, Drug discovery, Molecular biology

## Abstract

Krüppel-like factor 5 (KLF5) is an intrinsically disordered transcription factor involved in cardiac remodeling, cancer, and metabolic diseases. Targeting KLF5 has been a persistent challenge in drug development due to its structural inaccessibility. We investigated cardioprotective effects of NC114, a rationally designed small molecule that mimics a short, hydrophobic α-helical motif in KLF5, thereby disrupting its protein–protein interactions. Adult C57BL/6J male mice underwent transverse aortic constriction (TAC) or sham surgery, followed by administration of NC114 or vehicle. NC114-treated TAC mice exhibited preserved cardiac function, reduced heart weight-to-body weight ratio, and markedly attenuated interstitial fibrosis. Gene expression analysis demonstrated decreased cardiac expression of *Klf5*, *Nppb*, *Tgfb1*, *PAI-1*, *Col1a1*, and *Fn1*. NC114 also suppressed oxidative stress and reduced phosphorylation of PKCδ and expression of HIF-1α during the early phase post-TAC. Metabolomic profiling revealed that NC114 treatment reversed TAC-induced accumulation of organic and amino acids. NC114, a novel peptidomimetic molecule, targets the undruggable transcription factor KLF5 to attenuate cardiac hypertrophy, fibrosis, and metabolic dysregulation in pressure overload-induced heart failure. This study highlights the potential of KLF5 inhibition as a therapeutic strategy in cardiovascular disease.

**Supplementary Information:**

The online version contains supplementary material available at 10.1038/s41598-025-32155-y.

## Introduction

Heart failure (HF) is a complex syndrome characterized by progressive maladaptive remodeling, fibrosis, and metabolic dysregulation, contributing to high global morbidity and mortality^1–3^. Despite advances in pharmacological and device-based therapies, current HF treatments focus primarily on hemodynamic support and symptom management, rather than on addressing fundamental molecular mechanisms that drive disease progression^4–6^. The transition from compensatory left ventricular (LV) hypertrophy to decompensated HF remains incompletely understood^7,8^. A key limitation in HF research is the lack of in vivo time-course analyses, which are essential to identify sequential molecular events leading to HF.

One of the major drivers of HF pathophysiology is Krüppel-like factor 5 (KLF5), a zinc-finger transcription factor involved in fibrosis, extracellular matrix (ECM) remodeling, metabolic reprogramming, and inflammation^9–12^. KLF5 expression is upregulated in response to pressure overload, ischemia, and metabolic dysfunction, promoting fibroblast activation, myocyte hypertrophy, and metabolic shifts^9,13–15^. Elevated KLF5 levels have been associated with adverse outcomes in HF, diabetic cardiomyopathy, vascular disease, and cancer, underscoring its central role in cellular stress responses^16–18^. Experimental models have demonstrated that KLF5 deletion or inhibition alleviates cardiac fibrosis and improves myocardial adaptation to stress, suggesting that KLF5 could be a promising therapeutic target for HF^9^. Recent studies have further shown that acute inhibition or cardiomyocyte-specific deletion of KLF5 also attenuates myocardial ischemia/reperfusion injury, reducing infarct size and inflammation while preserving systolic function, thereby highlighting its pathogenic role in both chronic and acute cardiac injury^19^. However, no pharmacological agents specifically targeting KLF5 have been developed to date, and its role in HF progression remains unknown.

In addition to fibrosis, oxidative stress and mitochondrial dysfunction are hallmarks of HF progression^20,21^. One of the major mediators of oxidative stress in HF is protein kinase C delta (PKCδ). It is activated in response to the accumulation of reactive oxygen species (ROS), which contribute to inflammation, mitochondrial damage, and cardiomyocyte apoptosis^22,23^. Persistent PKCδ activation has been implicated in adverse cardiac remodeling and HF progression^23,24^. Given the interconnected roles of KLF5^25^ and PKCδ^24^ in HF pathogenesis, a therapeutic strategy targeting both pathways could provide significant cardioprotective benefits.

NC114 is a peptidomimetic molecule, originally designed as an anticancer agent that inhibits KLF5^26^. NC114 effectively inhibits tumor growth and induces growth arrest in colorectal cancer cells by preventing PKCδ activation and FOXM1 nuclear translocation, without significant side effects^27^. By mimicking the helical motif of KLF5, NC114 suppresses its transcriptional activity, leading to reduced fibrosis, oxidative stress, and metabolic dysfunction. Given the mechanistic overlap between KLF5-regulated pathways in oncology and cardiology, we hypothesized that NC114 might attenuate pathological cardiac remodeling, thereby preventing HF progression. However, its potential cardioprotective effects have not been explored.

This study investigated effects of NC114 on cardiac remodeling, its sustained benefits following short-term treatment, molecular pathways that it modulates, and its role in mitigating oxidative stress and myocardial injury. To investigate therapeutic effects of NC114, we employed a transverse aortic constriction (TAC) mouse model of pressure overload-induced HF^28^. Unlike previous studies that focused on static endpoints, our study integrated in vivo time-course analysis, evaluating molecular and functional changes at 1 week and 4 weeks post-TAC. This approach allowed us to track progression from early hypertrophic adaptation to overt HF, providing critical insights into early molecular events that drive disease progression.

Our findings demonstrate that NC114 significantly improved survival, preserved cardiac function, and attenuated pathological remodeling in TAC mice. NC114-treated TAC mice had higher ejection fraction (EF), lower heart weight to body weight (HW/BW) ratios, and less fibrosis than vehicle-treated TAC mice. Transcriptomic and metabolomic analyses revealed that NC114 downregulated fibrosis-related genes (*Tgfb1*, *Col1a1*), reduced oxidative stress markers (*Nox4*, *Bcl2*), preserved glycine levels, improved the glutathione/glutathione disulfide (GSH/GSSG) ratio, and suppressed PKCδ activation. Notably, beneficial effects persisted even though NC114 was administered only during the first 10 days post-TAC, suggesting that early intervention can prevent HF progression. Unlike conventional HF therapies focused on hemodynamics, NC114 acts via KLF5 inhibition, offering a novel, mechanism-based approach that bridges oncology and cardiology.

## Results

### NC114 improves cardiac function and survival in TAC Mice

**Fig. 1:**
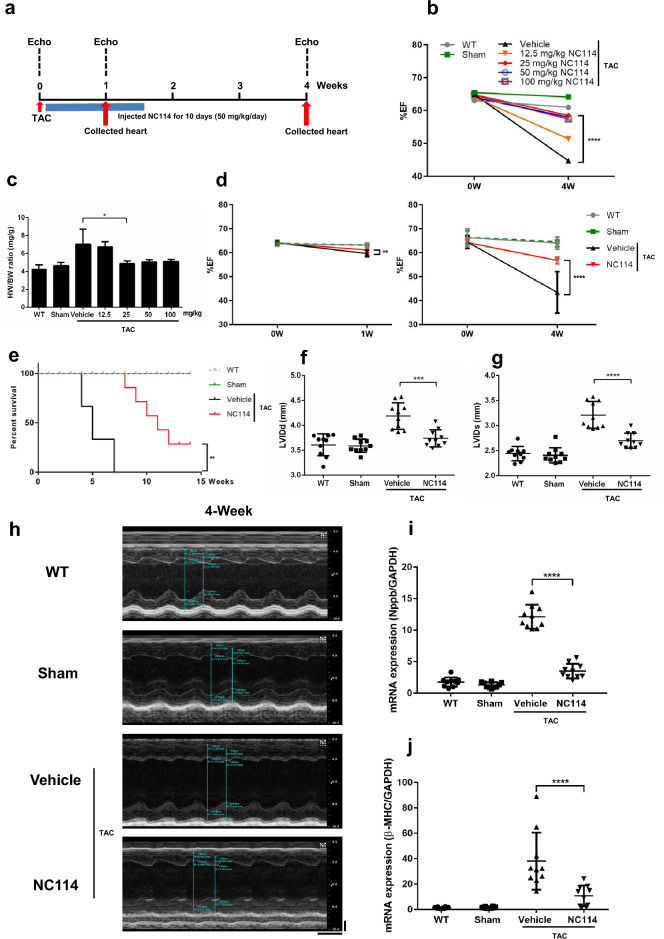
NC114 improves cardiac function and survival in TAC mice. (**a**) Schematic of the experimental design: NC114 was administered at 50 mg/kg/day for 10 days following transverse aortic constriction (TAC). Echocardiography was performed before TAC, and at 1 week and 4 weeks post-TAC. Hearts were collected for analysis, 1 week and 4 weeks post-TAC. (**b**) Comparison of ejection fraction (EF, %) with various doses of NC114 treatment (n = 3 per group). (**c**) Heart weight to body weight (HW/BW) ratios of WT, sham, vehicle, and NC114-treated TAC mice at different doses of NC114 (n = 3 per group). (**d**) Ejection fraction (EF, %) was measured in the left ventricle before TAC, and at 1 week and 4 weeks post-TAC (n = 10 per group). (**e**) Long-term survival curves showing significantly improved survival rates in NC114-treated TAC mice compared to untreated TAC mice (n = 3 per group; NC114, 50 mg/kg/day for 10 days). (**f** and **g**) Left ventricular internal dimensions at end-diastole (LVIDd) and end-systole (LVIDs) measured 4 weeks post-TAC, indicating improved systolic function in NC114-treated TAC mice (n = 10 per group). (**h**) Representative M-mode echocardiographic images of the left ventricle (LV), 4 weeks post-TAC. Vertical scale bar: 1 mm; horizontal: 100 ms. (**i** and **j**) Quantitative real-time PCR analysis of brain natriuretic peptide (*Nppb*) and β-myosin heavy chain (*β-MHC*) expression, demonstrating induction after TAC and subsequent reduction with NC114 treatment (n = 10 per group). GAPDH was used as the reference gene. Statistical analyses were performed using one-way and two-way ANOVA followed by Tukey’s multiple comparisons test. ****P < 0.0001, ***P < 0.001, **P < 0.01, *P < 0.05 vs. vehicle group.

C57BL/6J mice were randomly assigned to undergo LV pressure overload, which causes cardiac hypertrophy and HF, as shown in the experimental protocol (Fig. 1a). Two to three hours after TAC, mice were injected intraperitoneally with NC114 (50 mg/kg/day), twice a day for 10 days, and were classified as NC114-treated TAC mice. Echocardiography was performed before TAC and at 1 week and 4 weeks after surgery. Mice were sacrificed, and hearts were collected at 1 week and 4 weeks post-TAC for further analysis. NC114 was tested at four concentrations (12.5, 25, 50, and 100 mg/kg), administered intraperitoneally twice daily. A dose of 25 mg/kg per injection (50 mg/kg/day) proved effective in preventing HF induced by pressure overload in TAC mice, as evidenced by its ability to protect against the reduction in EF (Fig. 1b). NC114 treatment at 50 mg/kg/day for 10 days significantly reduced the HW/BW ratio (Fig. 1c) and mitigated the decline in EF, 1 week and 4 weeks post-TAC (Fig. 1d).

NC114 treatment also enhanced survival of TAC mice. Untreated TAC mice (vehicle) died within 7 weeks, whereas NC114-treated TAC mice survived as long as 12–14 weeks (Fig. 1e). Furthermore, left ventricular internal dimensions at end-diastole (LVIDd) and end-systole (LVIDs) at 4 weeks post-TAC were significantly reduced in NC114-treated TAC mice compared to those treated with vehicle (Fig. 1f-h).

At 4 weeks post-TAC, mRNA expression levels of HF markers, brain natriuretic peptide (*Nppb*) and β-myosin heavy chain (*β-MHC*), were significantly lower in NC114-treated TAC mice than in vehicle-treated TAC mice (Fig. 1i,j). These findings collectively suggest that NC114 effectively prevents deterioration of cardiac function and improves survival in TAC-induced HF.

### NC114 attenuates cardiac hypertrophy and reduces pressure overload-induced cardiac fibrosis

**Fig. 2:**
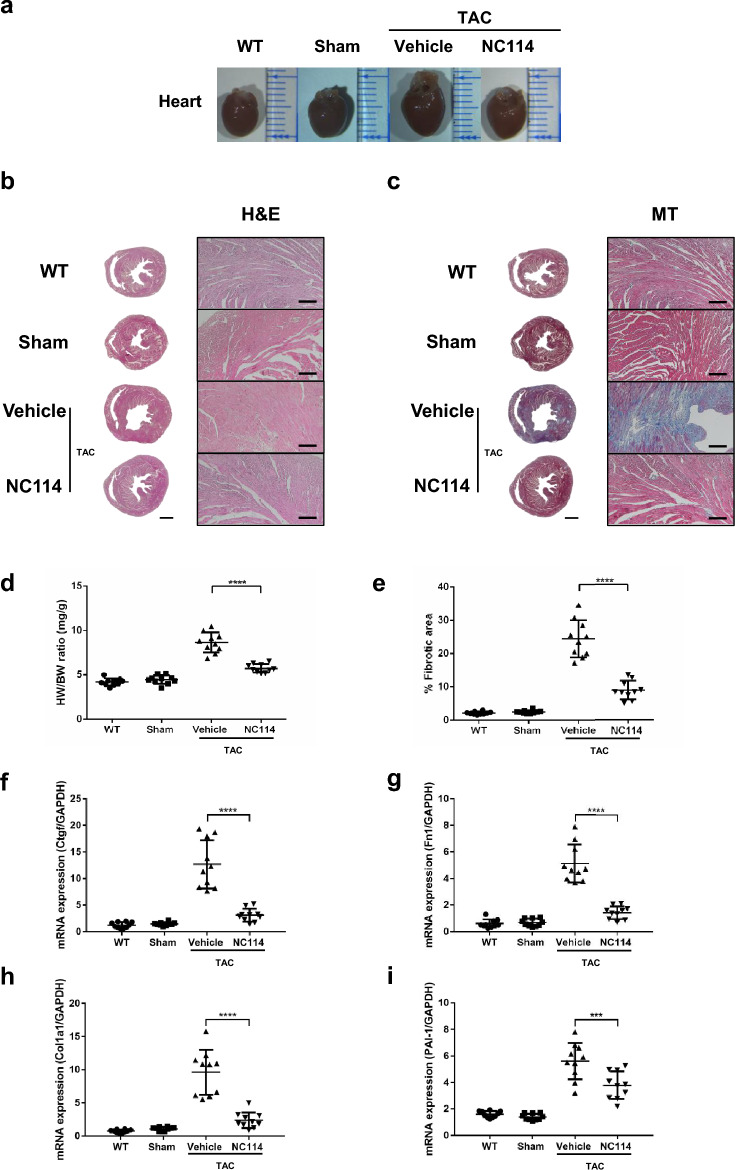
NC114 attenuates cardiac hypertrophy and reduces pressure overload-induced cardiac fibrosis. (**a**) Representative images of heart size from WT, sham, vehicle-treated TAC, and NC114-treated TAC mice at 4 weeks post-TAC. (**b**) Representative cross-sections of cardiomyocytes stained with hematoxylin and eosin (H&E). Scale bars: 2 mm (left) and 200 µm (right). (**c**) MT staining (MT) of cardiac tissues, highlighting fibrotic areas. Scale bars: 2 mm (left) and 200 µm (right). (**d**) Heart weight to body weight (HW/BW) ratios among indicated groups (n = 10 per group). (**e**) Quantification of fibrotic areas in indicated groups (n = 10 per group). (**f** to **i**) Quantitative real-time PCR analysis of connective tissue growth factor (*Ctgf*), Fibronectin (*Fn1*), Collagen Type I Alpha 1 Chain (*Col1a1*), and plasminogen activator inhibitor-1 (*PAI-1*) mRNA levels in left ventricles (LVs) of indicated groups (n = 10 per group). GAPDH was used as the reference gene. Statistical analyses were performed using one-way ANOVA followed by Tukey’s multiple comparisons test. ****P < 0.0001, ***P < 0.001 vs. vehicle group.

Morphological changes and histological analysis of heart sections stained with hematoxylin and eosin H&E revealed hypertrophic changes in mice subjected to TAC in the 4-week post-TAC group. Cardiac myocyte cross-sectional area (CSA) in NC114-treated TAC mice was significantly smaller than in vehicle-treated TAC mice (Fig. 2a,b; Supplementary Fig. 1). MT staining (MT) demonstrated reduced fibrosis in NC114-treated TAC mice compared to vehicle-treated TAC mice. (Fig. 2c).

NC114 treatment significantly reduced the HW/BW ratio in TAC mice at 4 weeks post-TAC, indicating attenuation of cardiac hypertrophy (Fig. 2d). Furthermore, interstitial fibrosis observed in cardiac tissues of TAC mice was markedly reduced in NC114-treated mice (Fig. 2e).

NC114 treatment decreased mRNA expression of several key markers of cardiac fibrosis at 4 weeks post-TAC, including connective tissue growth factor (*Ctgf*), fibronectin (*Fn1*), and Collagen Type I Alpha 1 Chain (*Col1a1*), compared to vehicle-treated mice (Fig. 2f-h). Additionally, plasminogen activator inhibitor-1 (*PAI-1*) expression was significantly reduced in NC114-treated TAC mice (Fig. 2i).

These findings demonstrate that NC114 effectively mitigates pressure overload-induced cardiac hypertrophy and fibrosis, suggesting its potential as a therapeutic agent for preventing maladaptive cardiac remodeling.

### NC114 reduces macrophage infiltration and inflammatory mediators in TAC-induced cardiac stress

**Fig. 3:**
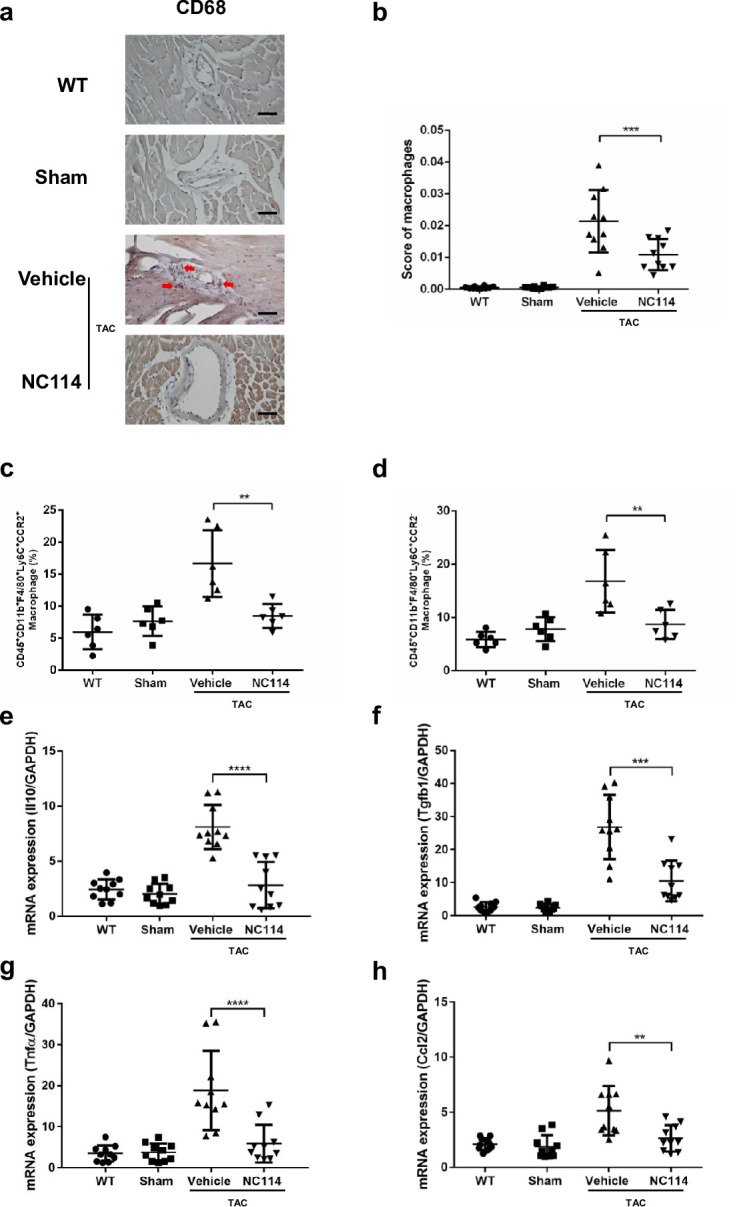
NC114 reduces macrophage infiltration and inflammatory mediators in TAC-induced cardiac stress. (**a**) Immunohistochemical analysis of CD68 in heart sections to detect macrophage infiltration. Red arrows indicate CD68-positive macrophages. Scale bar: 200 µm. (**b**) Quantification of macrophage infiltration (CD68-positive cells) in cardiac tissues using ImageJ (version 1.53e) (n = 10 per group). (**c** and **d**) Flow cytometry analysis of macrophage populations in heart tissues, specifically CD45⁺CD11b⁺F4/80⁺Ly6C⁺CCR2⁺ and CD45⁺CD11b⁺F4/80⁺Ly6C⁺CCR2⁻ subtypes, 4 weeks post-TAC (n = 6 per group). (**e** to **h**) Quantitative real-time PCR analysis of mRNA expression levels of inflammatory mediators, including interleukin 10 (*Il10*), transforming growth factor-β (*Tgfb1*), tumor necrosis factor-α (*Tnfα*), and chemokine ligand 2 (*Ccl2*) in the heart, 4 weeks post-TAC (n = 10 per group). GAPDH was used as the reference gene. Statistical analyses were performed using one-way ANOVA followed by Tukey’s multiple comparisons test. ****P < 0.0001, ***P < 0.001, **P < 0.01 vs. vehicle group.

Macrophage infiltration, as detected by CD68 staining, was significantly elevated in cardiac tissues following TAC, but was markedly reduced in NC114-treated TAC mice in the 4-week, post-TAC group (Fig. 3a,b). Flow cytometry analysis further confirmed that levels of both CD45⁺CD11b⁺F4/80⁺Ly6C⁺CCR2⁺ and CD45⁺CD11b⁺F4/80⁺Ly6C⁺CCR2⁻ macrophages were significantly decreased in NC114-treated TAC mice compared to vehicle-treated TAC mice (Fig. 3c,d). These results demonstrate that NC114 reduces both CCR2⁺- and CCR2⁻-resident cardiac macrophages in TAC mice.

In addition to reducing macrophage infiltration, NC114 treatment suppressed expression of key inflammatory mediators. The mRNA levels of interleukin 10 (*Il10*), transforming growth factor beta 1 (*Tgfb1*), tumor necrosis factor alpha (*Tnfα*), and chemokine (C–C motif) ligand 2 (*Ccl2*) were significantly lower in NC114-treated TAC mice than in vehicle-treated TAC mice (Fig. 3e-h). These findings indicate that NC114 effectively attenuates inflammation in TAC-induced cardiac stress by reducing macrophage infiltration and suppressing expression of pro-inflammatory mediators.

### NC114 preserves the cardiac transcriptomic profile upon pressure overload by TAC

RNA-sequencing revealed that the gene expression profile of NC114-treated TAC mice closely resembles that of wild-type (WT) controls, underscoring the protective effect of NC114 on cardiac transcriptional homeostasis. This conclusion was based on downstream analyses of RNA-sequencing data, including principal component analysis (PCA), which showed that transcriptomic profiles of NC114-treated TAC mice clustered near those of WT mice, whereas vehicle-treated TAC mice formed a distinct group at both 1 week and 4 weeks post-TAC (Supplementary Fig. 3). Supporting this, heatmap analysis of the top 100 differentially expressed genes (DEGs) demonstrated that TAC-induced changes (both upregulation and downregulation) were largely normalized toward a WT- or Sham-like pattern in NC114-treated mice (Supplementary Fig. 4). These findings indicate that NC114 treatment restores global gene expression toward a WT-like state under pressure overload conditions.

To further characterize the transcriptomic impact of NC114, we compared DEGs, defined as genes with an absolute log₂ fold-change (|logFC|) ≥ 1 and a false discovery rate (FDR) < 0.05, between TAC + Vehicle and WT mice (Supplementary Figs. 5–9), and between TAC + NC114 and TAC + Vehicle mice (Supplementary Figs. 10–14). Reactome-pathway-enrichment analysis revealed that NC114 reversed many TAC-induced transcriptional changes, often showing opposing effects in key biological pathways. Specifically, ECM-related pathways, including collagen and elastic fiber formation, were markedly upregulated in vehicle-treated TAC mice, but significantly downregulated by NC114 (Supplementary Figs. 6, 7, 13, and 14). Conversely, mitochondrial metabolic pathways, including the TCA cycle, respiratory electron transport, and fatty acid β-oxidation, were suppressed by TAC, but restored or enhanced by NC114 treatment (Supplementary Figs. 8, 9, 11, and 12). Collectively, these findings indicate that TAC induces pathological transcriptional remodeling characterized by ECM activation and mitochondrial suppression, whereas NC114 treatment counteracts these alterations and promotes a more homeostatic transcriptional state. At 4 weeks post-TAC, the restorative effect of NC114 on mitochondrial gene expression diminished and was no longer statistically significant, whereas suppression of ECM-related gene expression remained evident (Supplementary Figs. 12, 14). Enrichment of pathways downregulated in vehicle-treated TAC mice and upregulated by NC114 became less pronounced at this late stage, suggesting that while broad transcriptomic effects of NC114 wane over time, its anti-fibrotic influence persists.

**Fig. 4:**
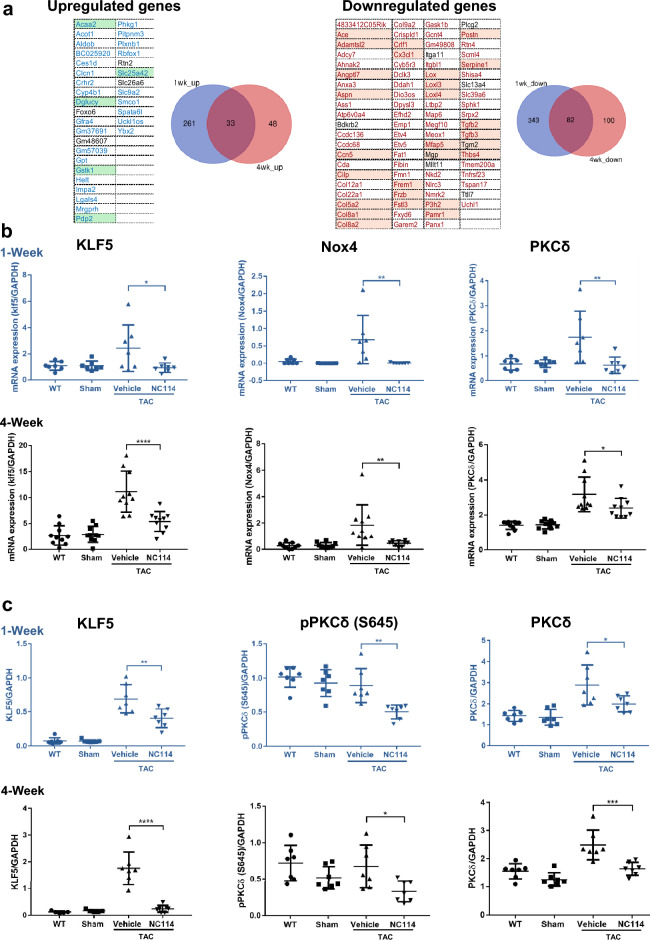
NC114 preserves the cardiac transcriptomic profile upon pressure overload by TAC. (**a**) Commonly upregulated and downregulated genes in 1-week and 4-week NC114-treated TAC mice, compared to vehicle-treated TAC mice. Commonly upregulated and downregulated genes at both time points are displayed below their respective diagrams. Note: Genes localized to the extracellular region (GOCC:0005576) and mitochondria (GOCC:0005739), based on subcellular compartment annotations, are highlighted in orange (27 genes) and green (5 genes), respectively, in the gene name column. Left: Venn diagram illustrating overlap of upregulated differentially expressed genes (DEGs) between 1-week (blue) and 4-week (red) NC114-treated mice compared to Vehicle-treated controls. Right: Venn diagram illustrating overlap of downregulated DEGs between 1-week (blue) and 4-week (red) NC114-treated mice compared to Vehicle-treated controls. (**b**) Quantitative real-time PCR analysis of mRNA expression *Klf5*, *Nox4*, and *PKCδ* in heart tissues, 1 week and 4 weeks post-TAC (n = 7–10 per group). GAPDH was used as the reference gene. (**c**) Western blot analysis of protein expression levels, including KLF5, pPKCδ (S645), and PKCδ in heart tissues from various groups, 1 week and 4 weeks post-TAC (n = 5-7 per group). GAPDH was used as a loading control. Representative blots are shown in Supplementary Figs.19, 21, 22 and 27. Data for 1 week are shown in blue. Statistical analyses were performed using one-way ANOVA followed by Tukey’s multiple comparisons test. ****P < 0.0001, ***P < 0.001, **P < 0.01, *P < 0.05 vs. vehicle group.

To better understand time-dependent transcriptional dynamics modulated by NC114, we analyzed overlapping DEGs 1 week and 4 weeks after TAC, using Venn diagrams. This analysis identified genes consistently dysregulated by TAC (Vehicle vs. WT; see Supplementary Fig. 15) and genes persistently reversed by NC114 (NC114 vs. Vehicle; see Fig. 4a; for details, see in Supplementary Fig. 16). In NC114-treated TAC mice, 294 genes were upregulated and 425 genes were downregulated at 1 week, whereas 81 and 182 genes were up-regulated and downregulated, respectively, at 4 weeks. Among these, 33 upregulated and 82 downregulated genes were identified at both 1 week and 4 weeks post-TAC (Supplementary Fig. 16). Importantly, 90.2% (74/82) of commonly downregulated genes were originally upregulated in vehicle-treated TAC mice (Supplementary Fig. 15a), indicating effective suppression of TAC-induced gene activation by NC114. Similarly, 87.9% (29/33) of commonly upregulated genes had been downregulated by TAC (Supplementary Fig. 15b), suggesting that NC114 restores suppressed mitochondria-associated gene expression patterns. Gene ontology analysis revealed that these reversals predominantly involved transcripts localized to the extracellular region (GOCC:0,005,576) or mitochondria (GOCC:0,005,739), supporting the dual role of NC114 in attenuating ECM remodeling and enhancing mitochondrial metabolic resilience.

Several persistently modulated DEGs emerged as key targets of NC114. Insulin-like Growth Factor-Binding Protein 7 (*Igfbp7*), transforming growth factors 2 and 3 (*Tgfb2* and *Tgfb3*), which encode secreted pro-fibrotic factors, were consistently downregulated, suggesting durable suppression of fibrotic signaling. In addition, Collagen type V alpha 2 chain (*Col5a2*), Collagen Type VIII Alpha 1 Chain (*Col8a1*), and Collagen Type VIII Alpha 2 Chain (*Col8a2*), which encode structural components of the extracellular matrix, were also persistently downregulated, indicating attenuation of ECM remodeling at the level of both signaling and matrix composition. In contrast, the Glutathione S-transferase kappa 1 (*Gstk1*) gene, which encodes a mitochondrial enzyme involved in redox regulation, was upregulated, indicating improved antioxidant defense. Additionally, *Igfbp7* and NADPH oxidase 4 (*Nox4*), genes previously linked to oxidative stress and fibrosis, were significantly upregulated in vehicle-treated TAC mice (Supplementary Fig. 15a). In NC114-treated hearts, both genes were significantly downregulated at 1 week post-TAC, whereas at 4 weeks they exhibited downward trends that did not reach statistical significance (log₂FC = –1.99 and –3.18; adj. p = 0.0039 and 0.0179 at 1 week; log₂FC = –1.16 and –3.20; adj. p = 0.0722 and 0.0529 at 4 weeks; data from Supplementary Materials, RNAseq_NCVH_1wk.xlsx and RNAseq_NCVH_4wk.xlsx). These results collectively support a model in which NC114 mitigates pressure overload-induced cardiac remodeling by suppressing ECM-related genes and restoring mitochondrial gene expression in a coordinated fashion.

To identify molecular mechanisms underlying adaptive effects of NC114, we examined mRNA expression of key regulatory genes, including *Klf5*, *Nox4*, *PKCδ*, B-Cell Leukemia/Lymphoma 2 (*Bcl2*), Bcl2 Associated X (*Bax*), *Igfbp7*, Bcl2 Antagonist/Killer 1 (*Bak1*), Interleukin 6 (*Il6*), Eukaryotic translation initiation factor 5A-1 (*eIf5A*), Peroxisome proliferator-activated receptor alpha (*PPAR-α*), Peroxisome proliferator-activated receptor delta (*PPAR-δ*), Peroxisome proliferator-activated receptor gamma (*PPAR-γ*), Forkhead box protein M1 (*FoxM1*), and *Gstk1* in hearts of TAC mice, 1 week and 4 weeks post-TAC. Expression of *Klf5*, *Nox4*, *PKCδ*, *Bcl2*, *Bak1*, *Il6* and *Igfbp7* was significantly upregulated in untreated TAC mice, but was markedly downregulated in NC114-treated TAC mice at both 1 week and 4 weeks post-TAC. Expression of *PPAR-α*, *Gstk1*, and *Bax* was significantly downregulated in untreated TAC mice, but was markedly upregulated in NC114-treated TAC mice at both 1 week and 4 weeks post-TAC. Expression of *eIF5A* and *PPAR-δ* did not differ between NC114-treated TAC mice and untreated TAC mice in either the 1-week or 4-week group. *PPAR-γ* gene expression was significantly upregulated in NC114-treated TAC mice at 4 weeks post-TAC, whereas *FoxM1* gene expression was significantly downregulated in NC114-treated TAC mice at 1 week post-TAC (Fig. 4b; Supplementary Figs. 17,18).

Moreover, KLF5, phosphorylated PKCδ (pPKCδ) (S645), and PKCδ protein levels were significantly reduced in NC114-treated TAC mice both 1 week and 4 weeks post-surgery. Notably, the level of hypoxia-inducible factor 1-alpha (HIF-1α) was significantly reduced in NC114-treated TAC mice at 1 week, but not at 4 weeks. Conversely, PPAR-α levels were significantly elevated in NC114-treated TAC mice at 4 weeks, but not at 1 week (Fig. 4c; Supplementary Figs. 19,20).

These findings suggest that NC114 triggers a transcriptional response that mitigates pressure overload-induced stress by regulating stress-related and metabolic genes. NC114 likely helps preserve cardiac function and prevents maladaptive remodeling in TAC mice by modulating key pathways involved in cardiac adaptation and remodeling.

### NC114 enhances metabolic flexibility and modulates substrate metabolism in TAC mice

**Fig. 5:**
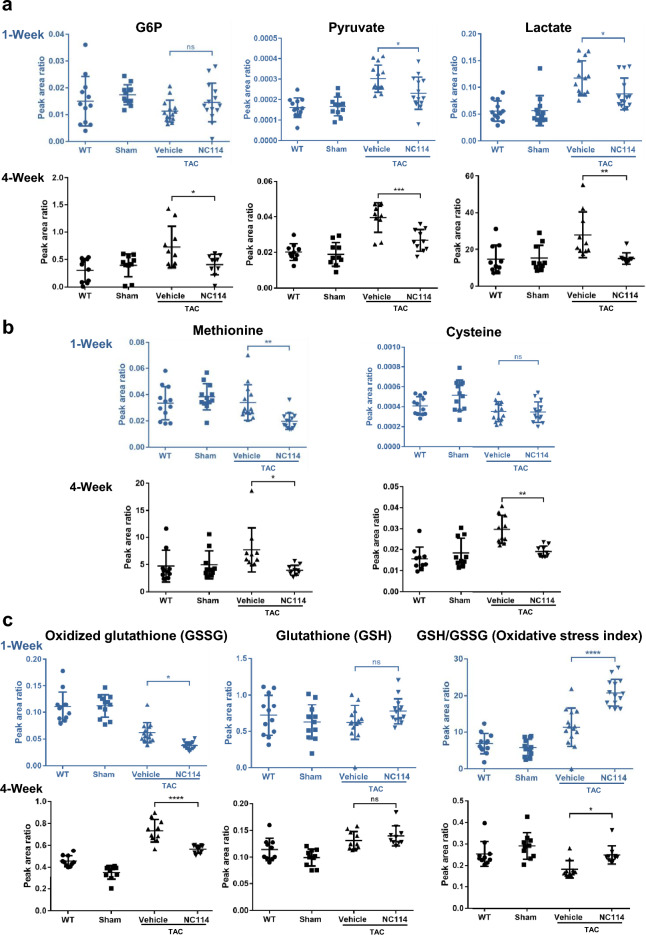
Substrate metabolism and metabolic flexibility in NC114-treated TAC mice (1 and 4 weeks post-TAC). Metabolic profiling of heart tissues from WT, sham, vehicle-treated TAC, and NC114-treated TAC mice was performed to analyze glycolysis pathway metabolites, methionine and cysteine pathway intermediates, and branched-chain amino acids (BCAAs) (n = 12–14 per group for 1-week TAC mice group, n = 10 per group for 4-week TAC mice). Data were normalized to an internal control, with units indicated as the area ratio where applicable. (**a**) Glucose-6-phosphate (G6P), pyruvate, and lactate levels were significantly lower in NC114-treated TAC mice compared to vehicle-treated TAC mice. (**b**) NC114 effectively lowered increased levels of methionine and cysteine that were observed in TAC mice. (**c**) The oxidative stress index, calculated from the ratio of reduced glutathione (GSH) to oxidized glutathione (GSSG) (GSH/GSSG), showed improvement in NC114-treated TAC mice. Data for 1 week are shown in blue. Statistical analyses were performed using one-way ANOVA followed by Tukey’s multiple comparisons test. ns = not significant; ****P < 0.0001, ***P < 0.001, **P < 0.01, *P < 0.05 vs. vehicle group.

To explore metabolic alterations in TAC mice and effects of NC114, we analyzed cardiac metabolism 1 week and 4 weeks post-TAC. TAC mice treated with NC114 showed reduced Glucose 6-phosphate (G6P) at 4 weeks, but not at 1 week. NC114-treated TAC mice exhibited significantly lower levels of pyruvate and lactate than untreated TAC mice, both 1 week and 4 weeks post-TAC (Fig. 5a), suggesting a shift in glycolytic activity. NC114 reduced elevated methionine levels observed in TAC mice both 1 week and 4 weeks post-TAC. It also decreased elevated cysteine levels at 4 weeks, but not at 1 week (Fig. 5b). A diagram illustrating metabolism of the glycolysis pathway, along with methionine and cysteine metabolism, is shown in Supplementary Fig. 23.

Oxidative stress was assessed by measuring the ratio of reduced glutathione (GSH) to oxidized glutathione (GSSG). The GSH/GSSG ratio was increased in NC114-treated TAC mice 1 week post-TAC, but was moderately reduced in NC114-treated TAC mice and further diminished in untreated TAC mice, 4 weeks post-TAC (Fig. 5c).

**Fig. 6:**
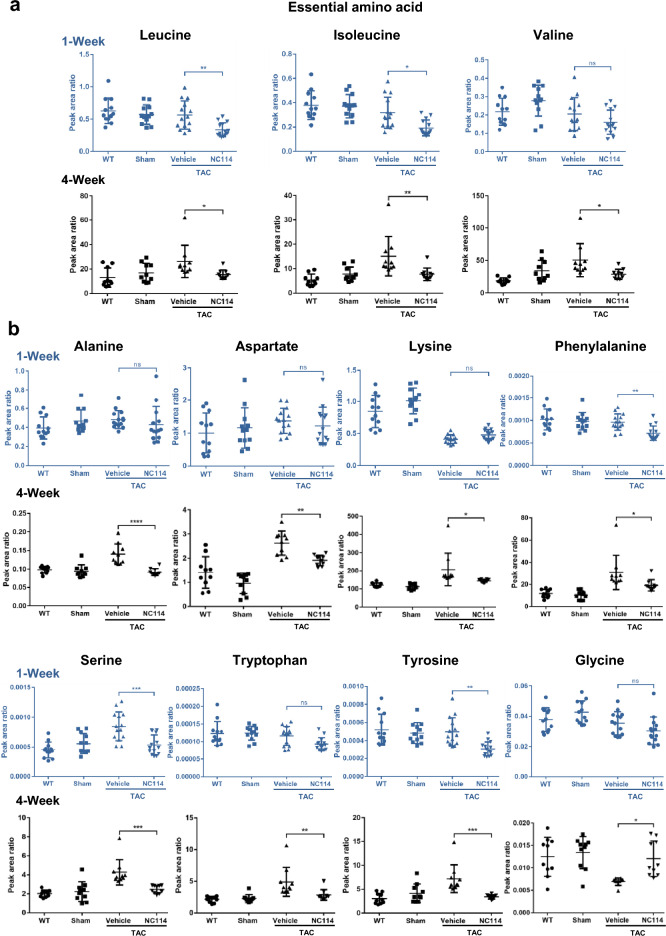
Levels of essential amino acids in NC114-treated TAC mice (1 and 4 weeks post-TAC). (**a**) Levels of essential amino acids, including leucine, isoleucine, valine (branched-chain amino acids, BCAAs) (n = 12–14 per group for 1-week TAC mice group, n = 10 per group for 4-week TAC mice). (**b**) Levels of multiple amino acids, including alanine, aspartate, lysine, phenylalanine, serine, tryptophan, tyrosine, and glycine were analyzed in heart tissues of all groups (n = 12–14 per group for 1-week TAC mice group, n = 10 per group for 4-week TAC mice). Data for 1 week are shown in blue. Statistical analyses were performed using one-way ANOVA followed by Tukey’s multiple comparisons test. ns = not significant; ****P < 0.0001, ***P < 0.001, **P < 0.01, *P < 0.05 vs. vehicle group.

Levels of various amino acids, including leucine, isoleucine, phenylalanine, serine, and tyrosine, were significantly elevated in both 1-week and 4-week TAC mice. However, NC114 treatment markedly decreased these amino acid levels. In contrast, glycine levels, which were significantly reduced in TAC mice, were preserved in NC114-treated TAC mice, 4 weeks post-TAC, but not 1 week post-TAC. Moreover, NC114 treatment significantly decreased levels of valine, alanine, aspartate, lysine, and tryptophan, 4 weeks post-TAC, but not 1 week post-TAC (Fig. 6a,b).

Metabolomic analysis revealed that TAC mice exhibited increased levels of metabolites associated with glycolysis and with methionine and cysteine metabolism, as well as amino acids (excluding glycine), compared with WT and sham controls. NC114 treatment effectively reduced these metabolite levels, though no significant alterations were observed in tricarboxylic acid (TCA) cycle intermediates (Supplementary Fig. 24).

Furthermore, analysis of acyl-carnitines indicated that short-, medium-, and long-chain acyl-carnitine levels were increased in NC114-treated TAC mice compared to untreated TAC mice (Supplementary Fig. 25a-c). However, short-chain acyl-carnitine (C0) and long-chain acyl-carnitine (C14 and C16) levels did not change between NC114-treated TAC mice and untreated TAC mice, 1 week post-TAC. Moreover, analysis of acyl-CoA and malonyl-CoA levels showed no change between NC114-treated and untreated TAC mice at either 1 week or 4 weeks (Supplementary Fig. 26).

These findings suggest that NC114 promotes metabolic flexibility and regulates substrate metabolism, potentially mitigating metabolic dysregulation induced by pressure overload in TAC mice.

## Discussion

Cardiotoxicity associated with anti-cancer drugs is increasingly recognized as critical in oncology and cardiovascular medicine. These drugs, while effective in treating various cancers^29–32^, often lead to severe cardiac complications, including HF. This dual challenge complicates patient management, as survivors of cancer are at heightened risk of cardiovascular diseases^30,33^. Certain compounds have shown promise not only in avoiding cardiotoxic effects, but also in providing cardioprotective benefits. NC114 effectively suppresses colorectal cancer cell proliferation without significant side effects^26,27^ and in this study, it has demonstrated the capacity to protect against pressure overload-induced HF in TAC mice. This dual efficacy positions NC114 as a unique therapeutic agent with potential applications in both oncology and cardiology.

KLF5 is essential in cardiovascular remodeling, acting as a transcriptional regulator that promotes cardiac hypertrophy and fibrosis under stress conditions such as pressure overload^9,15,34^. Its expression is significantly upregulated in TAC models, contributing to fibroblast proliferation and maladaptive cardiac remodeling^9,35^. Mylonas et al. recently demonstrated that cardiomyocyte-specific deletion of KLF5 reduced infarct size and improved cardiac function following ischemia–reperfusion injury in mice^19^. NC114 targets KLF5 directly to modulate HF mechanisms at the transcriptional level. Of note, ML264, a small molecule previously reported by Ruiz de Sabando et al., reduces KLF5 mRNA and protein expression^36^. However, ML264 acts primarily through suppression of early growth response 1 (EGR1), a transcriptional activator of KLF5; therefore, it is considered an inhibitor of the EGR1/KLF5 signaling axis. In contrast, NC114 represents a novel and more targeted approach to KLF5 inhibition. Unlike ML264, NC114 directly inhibits KLF5 by mimicking its helical motif, making it a more selective and effective therapeutic with a unique mechanism for targeting KLF5-related pathologies. In TAC mice, NC114 improved cardiac function (higher EF, lower HW/BW) and preserved LV structure, highlighting its potential to prevent early HF progression. RNA sequencing and mRNA expression analysis revealed downregulation of fibrosis-related genes, including *Tgfb1*, *Col1a1*, and *PAI-1*, in NC114-treated TAC mice. These genes are critical for ECM buildup, a hallmark of maladaptive remodeling^18,37,38^. NC114 reduced *β-MHC* and *Nppb* levels, key markers of cardiac hypertrophy and HF, indicating that it mitigates maladaptive transcription from pressure overload. By inhibiting KLF5, NC114 disrupts fibrosis, inflammation, hypertrophy, and oxidative stress pathways, possibly involving FoxM1 regulation. FoxM1 was increased in 1-week TAC mice and then reduced at 2 weeks^39^, consistent with our results, which show a significant difference at 1 week in NC114-treated TAC mice.

Inflammation is a central driver of HF progression^40^, with macrophages initiating and sustaining the inflammatory response^41,42^. KLF5 has been implicated in regulating cytokine expression and promoting macrophage infiltration into cardiac tissues under stress conditions^16^. Our study demonstrated that NC114 effectively reduced macrophage infiltration in TAC mice, including both CCR2⁺ and CCR2⁻ macrophage populations. These reductions likely reflect NC114’s ability to limit monocyte recruitment and macrophage proliferation during early stages of cardiac remodeling. In addition to its effects on macrophages, NC114 suppressed expression of key inflammatory mediators, including *Il10*, *Tgfb1*, *Tnfα*, and *Ccl2*. This broad anti-inflammatory effect may contribute to attenuation of HF by disrupting the inflammatory feedback loop that exacerbates cardiac dysfunction. Fibrosis, another critical component of HF pathology^43^, was significantly reduced in NC114-treated TAC mice. Histological analysis and mRNA expression showed decreased fibrotic areas and lower expression of *Fn1*, *Col1a1*, and *PAI-1*. *Tgfb1*, a downstream target of KLF5 and a major driver of ECM synthesis, was also suppressed in NC114-treated TAC mice. These findings suggest that NC114 mitigates fibrosis by targeting the upstream transcriptional regulator KLF5 and associated pathways.

Metabolic dysregulation is a hallmark of pressure overload-induced HF, characterized by shifts in glycolysis, amino acid metabolism, and oxidative stress^44^. NC114 treatment significantly altered these metabolic pathways, restoring metabolic homeostasis in TAC mice. Levels of pyruvate and lactate^45^, which are elevated in TAC mice due to increased glycolytic flux, were markedly reduced following NC114 treatment. This indicates that NC114 restores the balance between glycolysis and oxidative phosphorylation, improving energy efficiency in stressed myocardium. Amino acid metabolism was also profoundly affected by NC114. Elevated levels of branched-chain amino acids (BCAAs)^45,46^, as well as other amino acids such as alanine, lysine, and phenylalanine, were normalized in NC114-treated TAC mice. Notably, glycine levels, which were reduced in untreated TAC mice, were preserved by NC114 treatment. Glycine exerts cardioprotective effects by attenuating cardiac hypertrophy and fibrosis^47^, suggesting a potential mechanistic link between NC114’s metabolic effects and its protective role in HF. Oxidative stress^48^, a major contributor to HF progression, was also mitigated by NC114. The GSH/GSSG ratio^49,50^, an indicator of redox status, was significantly improved in NC114-treated TAC mice. Gstk1 is related to oxidative stress, and its expression is negatively correlated with obesity and hypertrophic cardiomyopathy. It is expressed in peroxisomal and mitochondrial fractions, localized to peroxisomes^51^. Notably, RNA sequencing revealed upregulation of Gstk1, a key regulator of oxidative stress^51^, in NC114-treated TAC mice. Nox4 is primarily responsible for increased mitochondrial O_2_^−^ (superoxide anion) production in response to pressure overload in the mouse heart^52^. Expression of *Nox4* and *Gstk1* also supports reduction of oxidative stress in TAC mice treated with NC114. These findings indicate that NC114 reduces oxidative stress by enhancing antioxidant defenses, thereby protecting the myocardium from further damage.

**Fig. 7:**
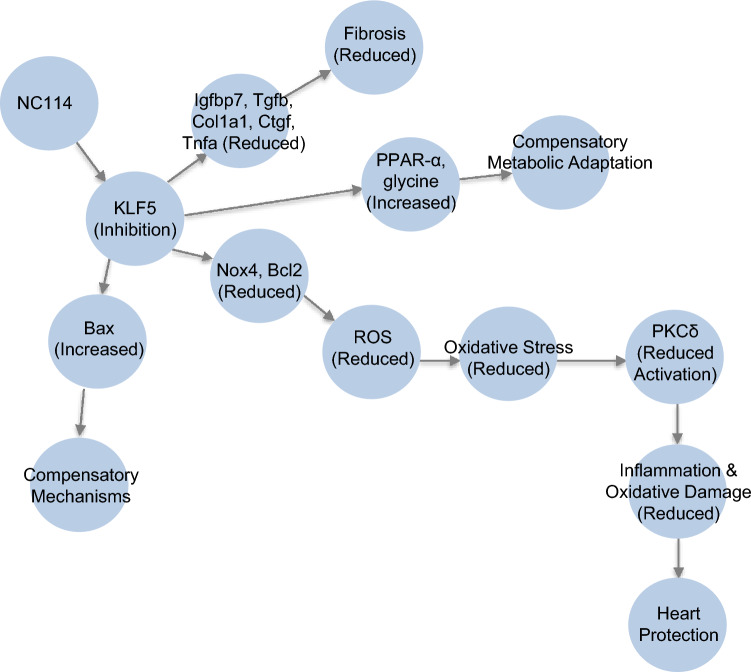
Mechanistic framework of NC114 efficacy in TAC–induced HF. Schematic representation showing how NC114 protects against HF from pressure overload. TAC upregulates KLF5 expression, which promotes fibrosis, oxidative stress, metabolic reprogramming, and production of abundant cytokines. These proinflammatory cytokines activate multiple molecular signaling pathways, leading to cardiomyocyte hypertrophy, apoptosis and fibroblast activation, ultimately resulting in cardiac remodeling and HF. However, NC114 tends to suppress these effects, highlighting the importance of early intervention in preventing maladaptive remodeling and HF progression. NC114 inhibits KLF5, leading to a reduction in *Igfbp7*, *Col1a1*, and cytokines such as *Tgfb*, *Ctgf*, and *Tnfα*, which decreases fibrosis. This, in turn, increases PPAR-α, glycine, and *Bax* levels, indicating a metabolic shift toward improved metabolism, compensatory metabolic adaptation, and additional compensatory mechanisms. Consequently, this shift results in a reduction of oxidative stress and apoptotic markers (*Nox4*, *Bcl2*), ultimately decreasing ROS production and PKCδ, reducing inflammation and oxidative damage, and providing heart protection.

By suppressing KLF5, NC114 reduces pro-fibrotic and pro-inflammatory cytokines such as *Tgfb1*, *Ctgf*, and *Tnfα*, thereby mitigating fibrosis, inflammation, and metabolic dysregulation. This results in increased levels of PPAR-α and glycine, indicating improved metabolism and adaptive responses. Consequently, oxidative stress and apoptotic markers, including ROS and PKCδ, decrease, protecting the heart from damage and preserving cardiac function under pressure overload (Fig. 7). Unlike conventional therapies focused on hemodynamic modulation, NC114 acts at the transcriptional level, addressing underlying causes of HF. Its cardioprotective properties are complemented by anti-cancer effects, highlighting its potential applications in both oncology and cardiology. Moreover, its ability to regulate oxidative stress and key metabolites further underscores its broad therapeutic scope. While the exact mechanism of KLF5 suppression remains unclear, one hypothesis under investigation is that NC114 promotes KLF5 degradation.

This study shows that NC114 significantly reduces phosphorylated and total PKCδ levels in TAC mice, suppressing PKCδ activation, which is a key driver of mitochondrial dysfunction, apoptosis, and adverse remodeling in HF. By suppressing PKCδ, NC114 may prevent progression from compensatory hypertrophy to maladaptive remodeling and HF. Moreover, inhibition of PKCδ leads to strong induction of *Ctgf* expression after Ang II treatment^53^. NC114 reduces PKCδ expression without increasing Ctgf, leading to decreased fibrosis. This suggests that NC114 can regulate PKCδ and fibrosis, supporting its potential as a therapeutic candidate to mitigate HF progression through targeted PKCδ modulation. KLF5 may indirectly influence PKCδ signaling through pathways that regulate oxidative stress, apoptosis, and inflammation^16^. PKCδ is often activated under pathological stress, including cardiac hypertrophy and cancer^54,55^. By inhibiting KLF5, NC114 may suppress PKCδ-related stress pathways, reducing inflammation and apoptosis in HF. It suppresses pro-inflammatory cytokine expression and PKCδ activation, which are often driven by oxidative stress, thereby limiting tissue damage and fibrosis. Further studies are needed to clarify its mechanisms and long-term efficacy in HF treatment.

Timing and dosage of NC114 administration are critical factors determining its efficacy. In our study, a dose of 50 mg/kg/day for 10 days effectively prevented HF when administered before onset of significant cardiac dysfunction. NC114-treated TAC mice exhibited prolonged survival, with lifespans extended to 12–14 weeks compared to 7 weeks in untreated TAC mice. Our data suggest that NC114 may also exert modest therapeutic effects when administered after HF onset, though further studies are needed to confirm this. These findings highlight the importance of early intervention to maximize cardioprotective benefits of NC114.

Comparison of 1-week and 4-week TAC mice highlights NC114’s dual benefits. Early treatment reduces acute remodeling, whereas long-term administration prevents fibrosis, hypertrophy, inflammation, and metabolic dysfunction. At 1 week, NC114 downregulates stress-related genes such as *Klf5* and *Nox4*, upregulates protective genes including *Gstk1* and *PPAR-α*, lowers HIF-1α levels, and prevents PKCδ activation. These actions enhance antioxidant defense and improve mitochondrial function. Even at 4 weeks, many benefits still persist, particularly in metabolic pathways, although some early effects, such as reduced HIF-1α, are more evident at the 1-week time point. NC114 continues to support redox balance and lipid metabolism, contributing to sustained cardiac function. These findings highlight NC114’s potential as a mechanism-driven therapy for HF, with early intervention providing lasting protection.

Despite promising results, this study has limitations. Heart rate differed between groups at both 1 and 4 weeks, and because EF measurements under isoflurane are sensitive to variations in HR, these differences may partly confound EF comparisons. Of note, our model makes it technically challenging to obtain EF or Doppler measurements immediately after TAC. Echocardiographic assessment at this early postoperative time point can adversely affect outcomes and may increase mortality in TAC-operated mice. This contrasts with the treatment group (Supplementary Fig. 29), in which EF or Doppler measurements can be safely performed at 4 weeks prior to initiating injections. Therefore, we were unable to perform EF or Doppler assessments before starting injections in the post-TAC mice. To assess the distribution of TAC severity between groups, mice were randomly assigned to either the untreated (vehicle) group or the NC114-treated group following TAC surgery. Transcriptomic and proteomic analyses provide molecular insights, but do not clarify mechanisms or confirm causality. While identifying therapeutic targets, functional studies are needed to understand how NC114 protects the heart. The mechanism by which NC114 suppresses KLF5 is unclear. It may promote KLF5 degradation, but this needs confirmation. Planned KLF5 knockout studies will attempt to clarify NC114’s role in cardiac remodeling, and further work is needed on related pathways. This study focused on macrophages in HF inflammation, without investigating functions of other immune cells, like T cells. NC114’s effects on lipid metabolism and other pathways also require investigation. Deaths in NC114-treated TAC mice may reflect model severity despite 4-week improvements. Long-term fibrosis progression and sustained benefits remain unknown. Moreover, this study was performed exclusively in male C57BL/6J mice to reduce variability; therefore, sex-specific differences in the response to TAC or NC114 treatment cannot be excluded. Future studies will include both male and female cohorts to evaluate potential sex-dependent effects. Lastly, NC114’s low solubility restricts clinical use, necessitating structural optimization. Future research should also explore its effects in established HF models.

In summary, NC114 is a novel KLF5 inhibitor with significant potential as a preventive agent against HF. By targeting KLF5, NC114 effectively reduces cardiac hypertrophy, fibrosis, inflammation, and oxidative stress while restoring metabolic balance. Its dual efficacy in cancer and HF highlights its utility as a therapeutic compound. While further research is needed to optimize its formulation and to explore its full therapeutic potential, NC114 shows promise as a novel treatment for both cardiovascular and oncological diseases.

## Methods

### Animal and pressure overload models

Eight-week-old male C57BL/6J mice, weighing between 18 and 22 g (Takasugi Experimental Animal Supply Co., Ltd., Japan), were housed under controlled conditions. All animal experiments were conducted in accordance with ARRIVE guidelines^56^ for ethical use and protection of animals in scientific research. The Institutional Animal Care and Use Committee of Jichi Medical University approved all mouse-related experimental procedures, which were performed following the committee’s guidelines. Mice were allowed food and water ad libitum under a 12-h light–dark cycle. Pressure overload was produced by transverse aortic constriction (TAC). Mice were randomly assigned to either the TAC group or a sham surgery group. Briefly, mice were anesthetized using an intraperitoneal injection of a mixture containing medetomidine (1 mg/mL), midazolam (5 mg/mL), and butorphanol tartrate (5 mg/mL) at a dosage of 0.3 mL per 100 g of body weight. This anesthetic combination was chosen for its complementary sedative and analgesic properties, which provided consistent and reliable anesthesia during experimental procedures. Anesthetic depth was evaluated by checking physical reflexes such as pedal withdrawal and corneal response, as well as monitoring respiratory patterns. Supplemental doses were administered as necessary to maintain anesthetic depth. Mice were placed in a supine position, and the skin was incised along the midline of the neck and chest, creating a longitudinal incision of approximately 1.5–2 cm. The aortic arch was then ligated with 6–0 silk, using an overlying 27-gauge needle to induce a narrowing in the vessel when the needle was removed. After the needle was withdrawn, a distinct constriction remained at the site. The suture was cut, and the skin was closed using 4–0 sutures in a continuous pattern. Post-surgery, mice were allowed to recover on a warming pad until fully awake. The sham group underwent the same procedure, except that the aortic arch was not ligated. A total of 280 mice were used for all experiments. Between 3 and 14 mice were utilized in each experiment, as detailed in the corresponding figure legend. Mice were euthanized under deep anesthesia to ensure unconsciousness and to prevent pain. Anesthetic depth was confirmed by the absence of reflexes such as pedal withdrawal and corneal response, along with stable respiratory patterns. Once surgical anesthesia was achieved, euthanasia was performed using an approved method such as cervical dislocation or transcardial perfusion, in accordance with ethical guidelines.

### Echocardiography

Echocardiography was performed on mice before TAC, 1 week after TAC, and 4 weeks after TAC or after sham surgery to assess heart function. Briefly, mice were placed on the heating table in a supine position with their extremities tied to the table with four electrocardiography leads. Chest fur was removed with a hair removal cream and ultrasound gel was applied to the thorax to improve visibility of cardiac chambers. After mice were anesthetized with 5% isoflurane for induction and 1–1.5% for maintenance, echocardiography was performed using a Vevo2100 equipped with a 30-MHz linear transducer (Vevo2100, FUJIFILM VisualSonics, Toronto, ON, Canada). The LV end-systolic diameter (LVESd), LV end-diastolic diameter (LVEDd), interventricular septal thickness at end-diastole (IVSd), interventricular septal thickness at end-systole (IVSs), LV posterior wall thickness at end-diastole (LVPWd), LV posterior wall thickness at end-systole (LVPWs), heart rate (HR) and ejection fraction (EF) were measured. EF was calculated and images were obtained to measure wall thickness. Measurements and analysis were performed by two individuals who were blinded to identities of experimental groups of mice. Then, mice were sacrificed to collect blood and heart tissue. Details are in the Supplementary Tables 2 and 3. To further clarify TAC severity, we provided additional postoperative metrics. We collected body-weight data from days 1–10 after surgery and observed a reduction in body weight in mice with moderate to severe TAC. These data were included in Supplementary Table 6, and the corresponding graph was presented in Supplementary Fig. 30. The graph of fractional shortening (FS, %) is presented in Supplementary Fig. 28.

### Treatments

Following TAC surgery, mice were randomly assigned to either the untreated group (vehicle) or the NC114-treated group. Daily NC114 administration was initiated on the day of surgery, and groups of mice received doses of either 12.5, 25, 50, or 100 mg/kg of NC114 for 10 days, administered intraperitoneally twice daily, to determine the optimal dose. That dose was 50 mg/kg/day and was subsequently used in all further experiments. Daily NC114 administration was initiated on the day of surgery and continued 7 days for the 1-week TAC mouse group and 10 days for the 4-week TAC mouse group. WT, sham, and vehicle groups were also injected with dimethyl sulfoxide (DMSO) and Solutol HS-15, continuing 7 days for the 1-week mouse group and 10 days for the 4-week mouse group. DMSO and Solutol HS-15 were used to dissolve NC114; therefore, they were also used for injections in control (WT and sham) and vehicle-treated mice. One week and four weeks post-TAC surgery, echocardiographic evaluations were performed, and hearts were excised for weighing to calculate the heart weight-to-body weight (HW/BW) ratio. Left ventricular (LV) tissues were collected for subsequent analyses.

### Histology

Heart tissues were harvested at designated times following TAC surgery and perfused with Phosphate Buffered Saline (PBS) through the left ventricle. After washing in cold PBS, hearts were fixed for 24 h in a rapid fixative solution (Sakura Finetek Japan Co., Ltd.), embedded in paraffin, and sectioned into 5-μm slices. These sections were mounted onto microscope slides and subjected to H&E staining and MT staining to assess histological and fibrotic changes. For H&E staining, paraffin sections were deparaffinized and rehydrated, and then stained using Carrazi’s hematoxylin solution (Muto Pure Chemicals, Ltd., Tokyo, Japan) for 5 min, followed by washing in tap water. Sections were differentiated in 1% acid alcohol, dehydrated in 95% ethanol, stained with eosin solution (Wako Pure Chemical Industries, Ltd.) for 3 min, and subsequently dehydrated using graded ethanol solutions (95% and 100%). Finally, sections were cleared in xylene and mounted using VectaMountTM (Vector). Histopathological analyses were conducted using a Keyence BZ-9000 microscope to evaluate global changes in heart size and fibrosis. CSA^57^ and fibrosis were quantified from five fields using ImageJ (10 cells/field). Briefly, images of cardiac slices were captured under a microscope at 40 × magnification. Five random fields from the left ventricle of each mouse were selected, and at least ten cells per field were analyzed to calculate CSA. CSA and fibrotic areas were quantified with ImageJ software.

### Immunohistochemistry

Paraffin-embedded heart tissues were sectioned into 5-μm slices and mounted on microscope slides. Sections were deparaffinized with xylene and rehydrated with graded alcohol solutions. After washing in tap water for 5 min, antigen retrieval was performed by heating sections in citric acid using a microwave for 8 min. Tissues were then quenched with BLOXALL® blocking solution for 10 min and washed with PBS buffer. For immunohistochemical staining, sections were processed using a Vectastain® Elite ABC-HRP Kit (Rabbit IgG) (Vector Labs, Cat. PK-6101) and ImmPACTTM DAB peroxidase substrate (Vector), following the manufacturer’s protocol. Briefly, sections were blocked with diluted normal goat serum for 20 min to prevent non-specific binding. They were then incubated overnight with an anti-CD68 antibody (Abcam, Cat. Ab125212) at a 1:1,000 dilution to detect macrophage accumulation. After washing with PBS, sections were incubated with biotinylated secondary goat anti-rabbit IgG for 30 min, followed by incubation with Vectastain Elite ABC reagent for 30 min. ImmPACTTM DAB substrate was applied for 2 min, and sections were rinsed with distilled water. Sections were counterstained with hematoxylin for 4 min, washed with distilled water, dehydrated with graded ethanol solutions (70%, 80%, 90%, and 100%), cleared in xylene, and mounted with VectaMountTM (Vector). Images were captured using a Keyence BZ-9000 microscope. Intensity of CD68 staining was quantified with ImageJ software.

### RNA extraction and quantitative real-time polymerase chain reaction (qRT-PCR)

Heart tissues were collected after PBS perfusion through the left ventricle, and washing in cold PBS. RNA was extracted from mouse LV tissues using an RNeasy Mini Kit (QIAGEN, Cat. 74106) and DNA contamination was removed with RNase-Free DNase digestion (QIAGEN, Cat. 79254) following the manufacturer’s protocol. RNA was washed and eluted. RNA concentration and purification were assessed with a Nanodrop1000 (Thermo Fisher Scientific). Then, cDNA was synthesized with ReverTra Ace® *reverse transcriptase* (*Toyobo*), according to the manufacturer’s protocol. Quantitative real-time PCR was conducted using an SYBR Premix Ex Taq II Kit (RR820A; Takara Biotechnology) and was performed using the Stratagene Mx3005P QPCR System (Agilent Technologies, Santa Clara, California, USA). First, 25 µL of the total reaction system was added to 2 SYBR Premix Ex Taq II (12.5 µl), 10 µmol/L forward primer (1 µL), 10 mmol/L reverse primer (1 µL), cDNA (2 µL), and RNase-free water (8.5 µL), and then the mixture was denatured at 95 ºC for 30 s. Next, the mixture was subjected to 40 cycles of amplification at 95 ºC for 5 s and annealing at 60 ºC for 30 s. Quantitative real-time PCR was run in 96-well plates and relative expression levels of target genes were determined after normalizing against GAPDH gene expression and quantified using the comparative threshold cycle method. Specific gene primer sequences used in the study are listed in Supplementary Table 1.

### RNA-sequencing and data analysis

Heart tissues were collected at specified time points from WT (0-week, 1-week, 4-week), sham (1-week, 4-week), vehicle-treated TAC mice (1-week, 4-week), and NC114-treated TAC mice (1-week, 4-week), with RNA-sequencing. Briefly, total RNA was extracted from heart tissue using an RNeasy Plus Universal Mini Kit (QIAGEN). cDNA libraries were generated from total RNA samples using a TruSeq Stranded mRNA Library Prep Kit, following the manufacturer’s instructions (Illumina, Cat. 20020595). cDNA libraries were sequenced using a 100-bp, paired-end read format on a NovaSeq 6000 (Illumina). Reads were mapped to the mouse reference genome GRCm39 using STAR aligner (version 2.7.11b) with gencode annotation vM34. Read quantification was carried out with the featureCounts function from the Rsubread package (v2.19.1) in R (v4.4.2). Differentially expressed genes (DEGs) were identified using the edgeR (v4.4.2) and limma (v3.62.2) packages. Raw read counts were normalized and transformed using the voom method in the limma package after filtering slightly expressed genes with filterByExpr. Differential expression analysis was performed using a linear model with lmFit, followed by empirical Bayes moderation with eBayes. DEGs were defined as genes with an absolute log₂ fold-change (|logFC|) ≥ 1 and an adjusted p-value (FDR) < 0.05. The list of DEGs was used for functional enrichment analysis with STRING (v12.0, http://string-db.org/). Reactome pathway and subcellular localization enrichment analyses were conducted using STRING, and significant terms were selected with an FDR ≤ 0.05. Hierarchical clustering heatmaps of the top 100 upregulated and downregulated DEGs were generated using pheatmap (v1.0.12) in R. For heatmap visualization, expression values were normalized using CPM (counts per million) and scaled by row (gene-wise z-score). Hierarchical clustering was performed using Euclidean distance and complete linkage.

### Western blotting

Proteins were extracted from cardiac tissues using T-PER® tissue protein extraction reagent (Thermo Scientific, Cat. 78510) supplemented with Halt Protease Inhibitor Cocktail (Thermo Scientific) and EDTA-Free protease inhibitors. T-PER reagent was added to tissue samples, which were then homogenized. Homogenates were centrifuged at 10,000 × g for 5 min to pellet cellular debris, and the resulting supernatant was collected. Protein concentrations were determined using a PierceTM BCA Protein Assay Kit (Thermo Scientific, Cat. 23225) following the manufacturer’s protocol. Protein samples were mixed with 5X loading buffer, denatured in a Thermo Alumi Bath (Iwaki, ABL-121) at 95°C for 10 min, cooled, and stored at -20°C until analysis. Equal amounts of protein from each sample, along with Precision Plus ProteinTM Dual Color Standards (Bio-Rad Laboratories, Cat. 1610374), were separated by sodium dodecyl sulfate polyacrylamide gel electrophoresis (SDS-PAGE) and transferred onto membranes using a gel transfer device. Membranes were incubated with primary antibodies at room temperature for 1 h, followed by incubation with secondary antibodies under the same conditions. Blots were visualized and analyzed using an infrared imaging system, and protein expression levels were normalized against GAPDH (Thermo Scientific, Cat. AM4300) as a loading control. Complete blot membranes are presented in the Supplementary Figs. 21, 22 and 27.

### Flow cytometry

Heart tissues were minced into small pieces and enzymatically digested using 2 mg/mL collagenase type IV (Worthington Biochemical Corporation) and 1.2 U/mL dispase II (Sigma-Aldrich) in Dulbecco’s phosphate-buffered saline (DPBS) supplemented with 0.9 mmol/L CaCl_2_. Tissues were incubated at 37°C for 15 min with gentle rocking. Partially digested tissues were triturated 10 times using a 10-mL serological pipette and incubated again at 37°C for another 15 min. This digestion process was repeated twice, and suspensions were placed on ice. Cell suspensions were filtered through a 40-µm cell strainer and transferred to 50-mL tubes containing 30 mL of DPBS. These suspensions were centrifuged at 250 × g for 5 min to collect cells and resulting cell pellets were resuspended in 250 µL of 2% fetal calf serum (FCS) in Hank’s Balanced Salt Solution (HBSS). To block non-specific binding, cells were incubated with CD16/32 antibody (BioLegend, TrueStain FcXTM) at 4°C for 1 h. Cells were then stained with primary antibodies in FACS buffer: anti-mouse CD45 (BioLegend, Alexa Fluor® 700), anti-mouse/human CD11b (BioLegend, Pacific BlueTM), anti-mouse F4/80 (BioLegend, PE/Cyanine7), anti-mouse Ly-6C (BioLegend, FITC), and anti-mouse CCR2 (R&D Systems, APC-conjugate). Stained cells were incubated in the dark at 4°C for 30 min. Flow cytometry analysis was performed using a Sony SH800 flow cytometer, and data were analyzed with FlowJo software (FlowJo, LLC). Gating strategies employed for analysis are presented in the Supplementary Fig. 2.

### Metabolomic analysis

Heart samples were randomly selected from each group for metabolomic analysis. Hearts were excised, immediately frozen in liquid nitrogen, and stored at -80°C until analysis. For each sample, 30–50 mg of heart tissue were homogenized in 500 µL of an internal standard solution for Liquid Chromatography Mass Spectrometry (LCMS), followed by addition of 250 µL ultrapure water. Homogenates were thoroughly mixed with 400 µL of chloroform, and mixtures were centrifuged at 15,000 rpm for 15 min at 4°C. Supernatant was collected, filtered through 0.5-mL Amicon® Ultra Centrifugal Filters (3K), and centrifuged again at 15,000 rpm for 90 min at 4°C. Resulting filtrates were lyophilized overnight using a TAITEC Ve-125 Centrifugal Concentrator. Dried samples were reconstituted in 100 µL ultrapure water and processed for analysis using LCMS. LCMS was performed with Shimadzu LCMS-8030 and LCMS-8050 systems.

### Statistical analysis

Statistical significance was determined by one- or two-way ANOVA followed by Tukey’s multiple comparisons test, as detailed in the figure legends. Analyses were performed using GraphPad Prism 7, with P < 0.05 considered significant.

## Supplementary Information

Supplementary Information 1.

Supplementary Information 2.

Supplementary Information 3.

Supplementary Information 4.

Supplementary Information 5.

Supplementary Information 6.

## Data Availability

Datasets generated and/or analyzed during the current study have been deposited in the DDBJ Sequence Read Archive under BioProject accession number PRJDB20375. Data that support the findings of this study are available from the corresponding author upon request.
