# Supplementary material for: Targeting the undruggable transcription factor, KLF5, with a peptidomimetic small molecule, NC114, attenuates pressure overload-induced cardiac remodeling and fibrosis
Source: Sci Rep. 2026 Jan 12;16:2367. doi: 10.1038/s41598-025-32155-y (PMC12816732; doi:10.1038/s41598-025-32155-y)
Supplement: Supplementary file 1 — Supplementary Information 1. [file 41598_2025_32155_MOESM1_ESM.pptx]

## Slide 1
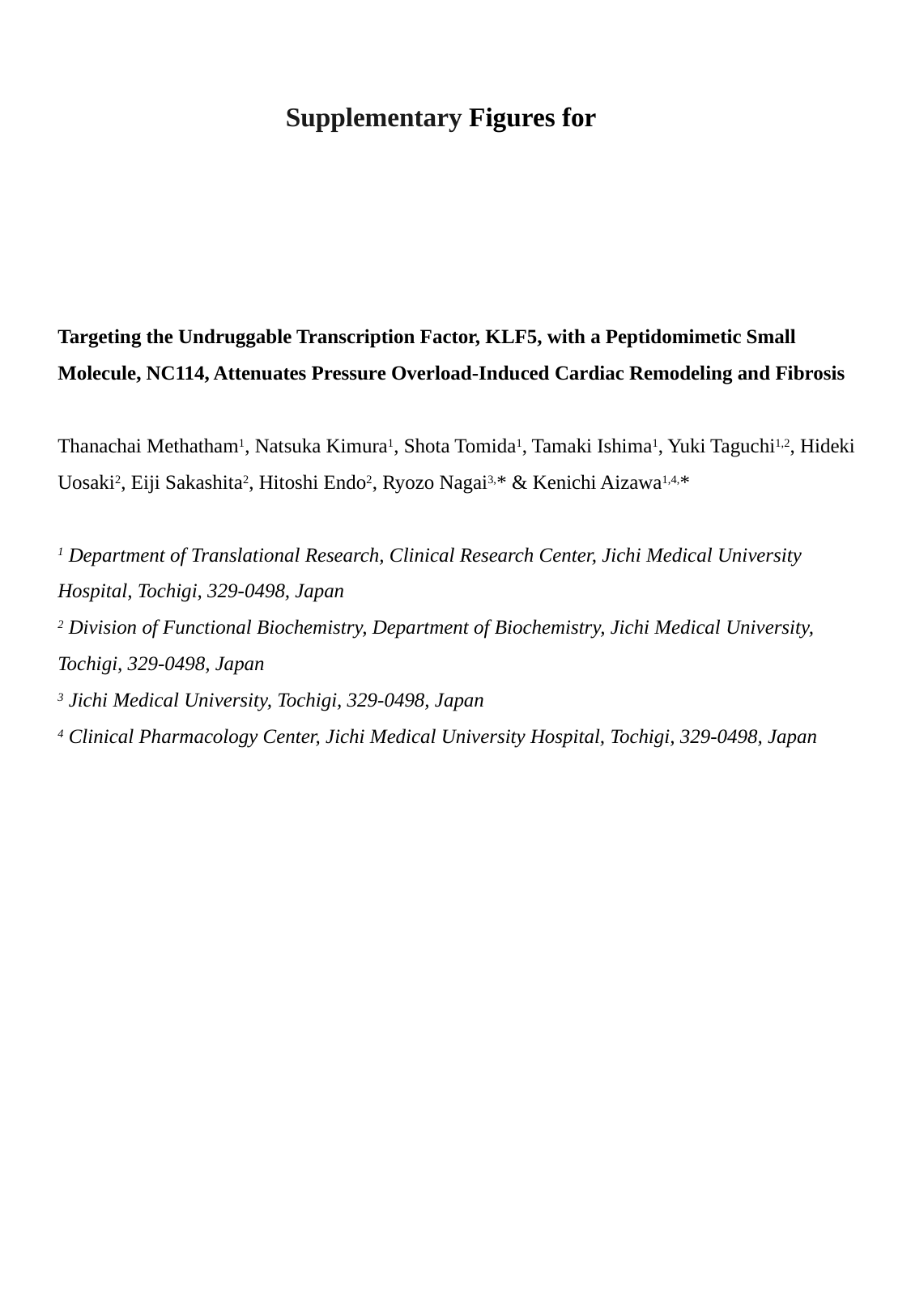

Supplementary Figures for
# Targeting the Undruggable Transcription Factor, KLF5, with a Peptidomimetic Small Molecule, NC114, Attenuates Pressure Overload-Induced Cardiac Remodeling and Fibrosis Thanachai Methatham1, Natsuka Kimura1, Shota Tomida1, Tamaki Ishima1, Yuki Taguchi1,2, Hideki Uosaki2, Eiji Sakashita2, Hitoshi Endo2, Ryozo Nagai3,* & Kenichi Aizawa1,4,*   1 Department of Translational Research, Clinical Research Center, Jichi Medical University Hospital, Tochigi, 329-0498, Japan 2 Division of Functional Biochemistry, Department of Biochemistry, Jichi Medical University, Tochigi, 329-0498, Japan 3 Jichi Medical University, Tochigi, 329-0498, Japan 4 Clinical Pharmacology Center, Jichi Medical University Hospital, Tochigi, 329-0498, Japan

## Slide 2
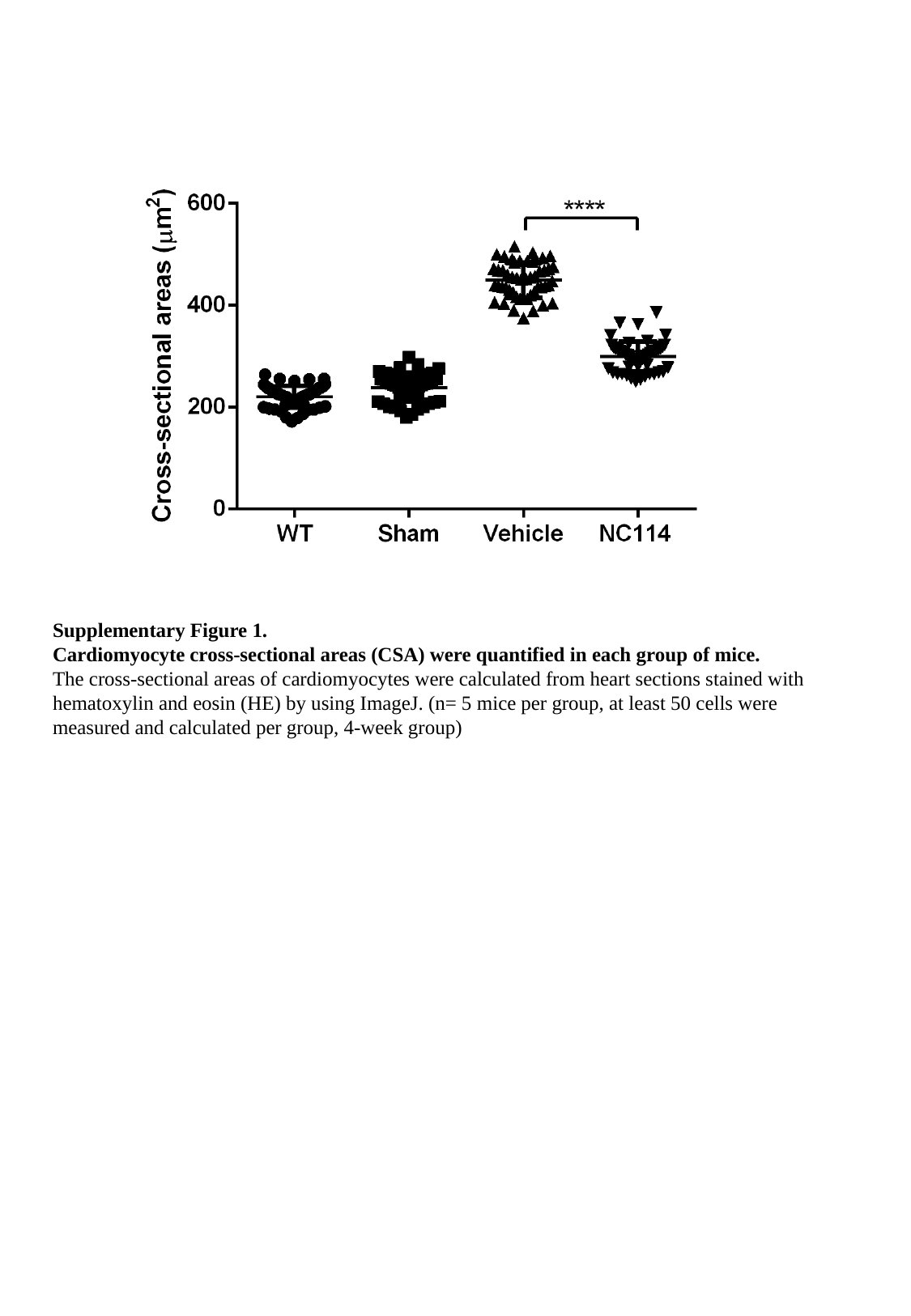

Supplementary Figure 1.
Cardiomyocyte cross-sectional areas (CSA) were quantified in each group of mice.
The cross-sectional areas of cardiomyocytes were calculated from heart sections stained with hematoxylin and eosin (HE) by using ImageJ. (n= 5 mice per group, at least 50 cells were measured and calculated per group, 4-week group)

## Slide 3
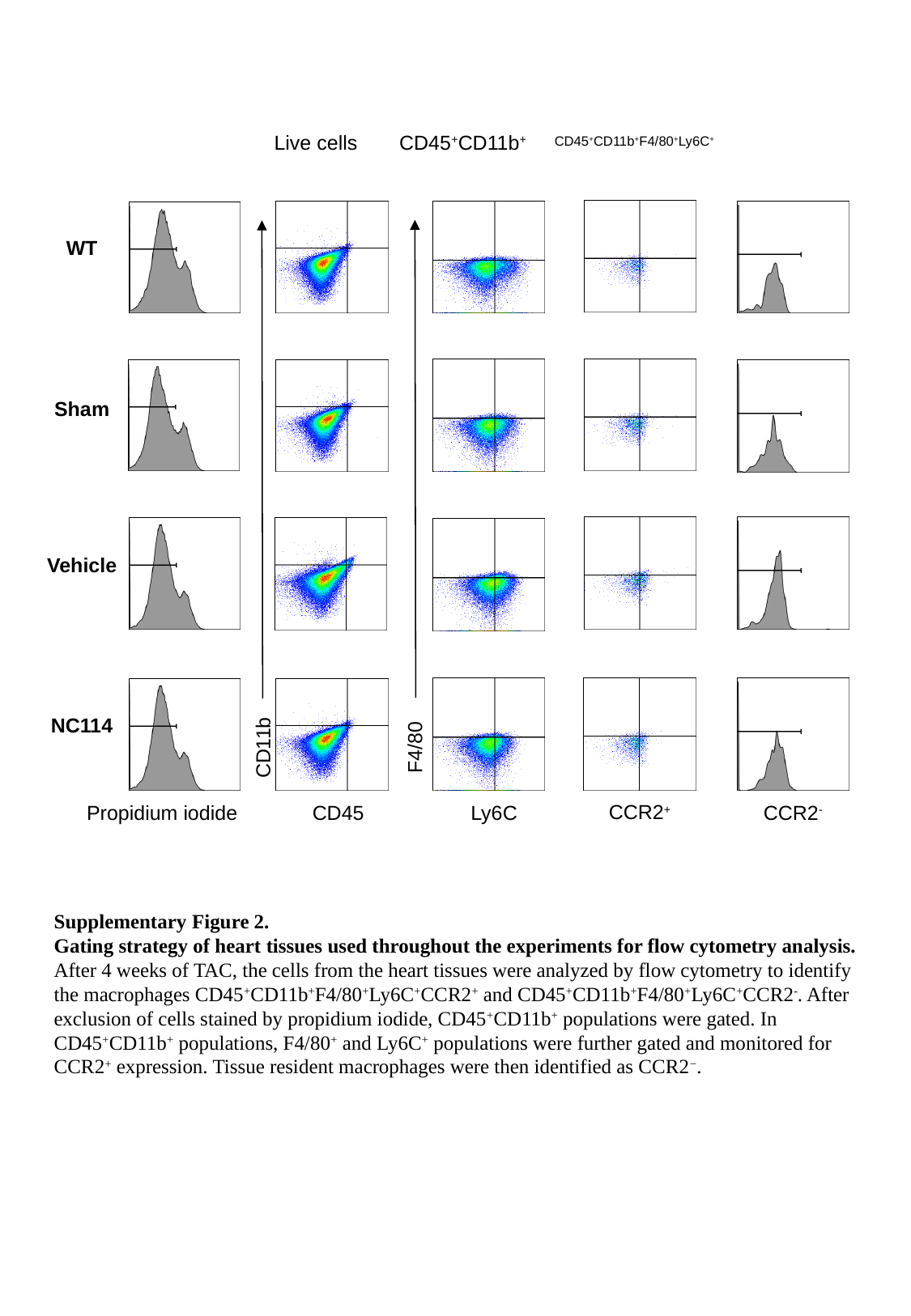

Live cells
CD45+CD11b+
CD45+CD11b+F4/80+Ly6C+
WT
Sham
Vehicle
NC114
CD11b
F4/80
CCR2+
Propidium iodide
CCR2-
CD45
Ly6C
Supplementary Figure 2.
Gating strategy of heart tissues used throughout the experiments for flow cytometry analysis. After 4 weeks of TAC, the cells from the heart tissues were analyzed by flow cytometry to identify the macrophages CD45+CD11b+F4/80+Ly6C+CCR2+ and CD45+CD11b+F4/80+Ly6C+CCR2-. After exclusion of cells stained by propidium iodide, CD45+CD11b+ populations were gated. In CD45+CD11b+ populations, F4/80+ and Ly6C+ populations were further gated and monitored for CCR2+ expression. Tissue resident macrophages were then identified as CCR2−.

## Slide 4
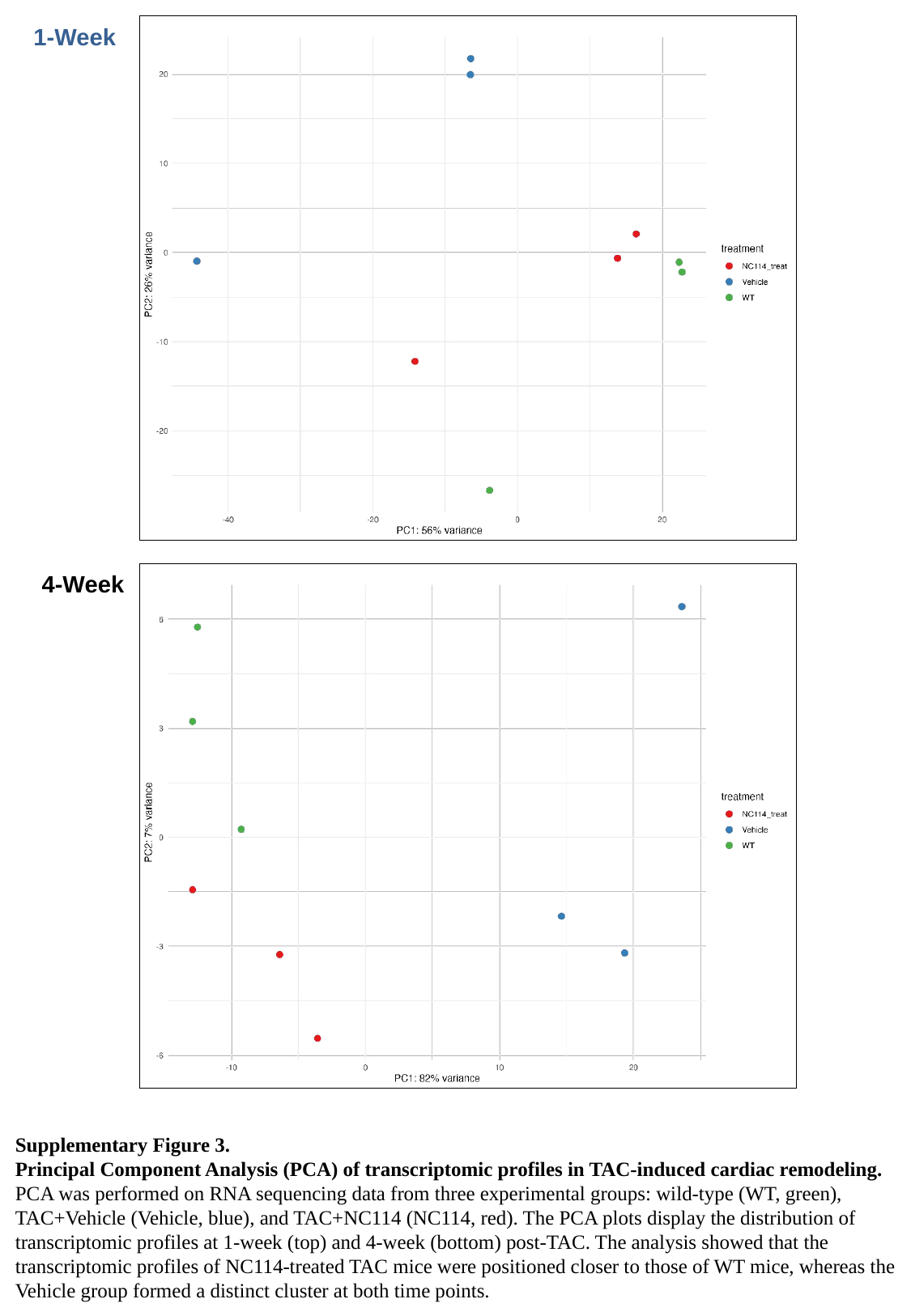

1-Week
4-Week
Supplementary Figure 3.
Principal Component Analysis (PCA) of transcriptomic profiles in TAC-induced cardiac remodeling.
PCA was performed on RNA sequencing data from three experimental groups: wild-type (WT, green), TAC+Vehicle (Vehicle, blue), and TAC+NC114 (NC114, red). The PCA plots display the distribution of transcriptomic profiles at 1-week (top) and 4-week (bottom) post-TAC. The analysis showed that the transcriptomic profiles of NC114-treated TAC mice were positioned closer to those of WT mice, whereas the Vehicle group formed a distinct cluster at both time points.

## Slide 5
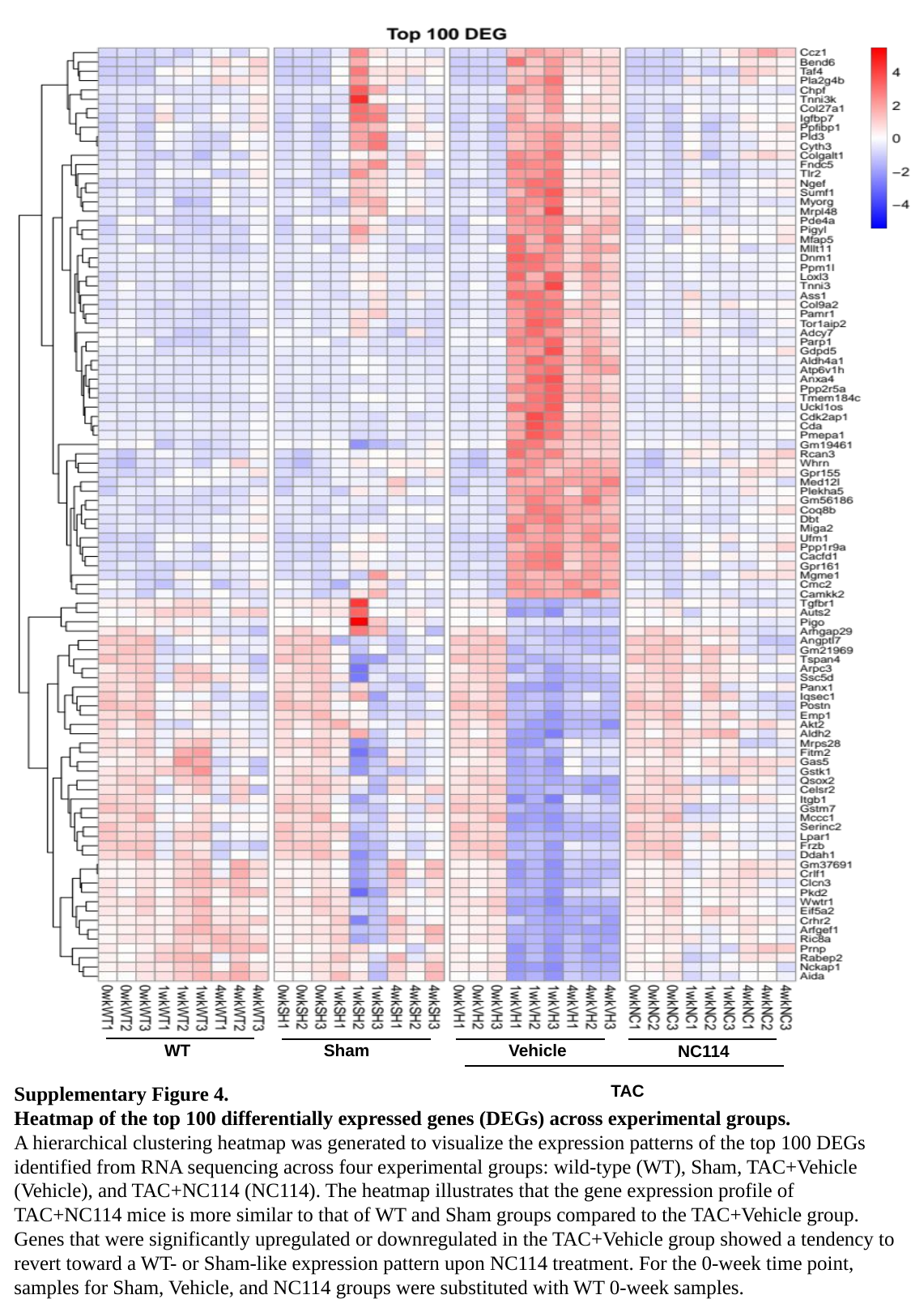

WT
Sham
Vehicle
NC114
TAC
Supplementary Figure 4.
Heatmap of the top 100 differentially expressed genes (DEGs) across experimental groups.
A hierarchical clustering heatmap was generated to visualize the expression patterns of the top 100 DEGs identified from RNA sequencing across four experimental groups: wild-type (WT), Sham, TAC+Vehicle (Vehicle), and TAC+NC114 (NC114). The heatmap illustrates that the gene expression profile of TAC+NC114 mice is more similar to that of WT and Sham groups compared to the TAC+Vehicle group. Genes that were significantly upregulated or downregulated in the TAC+Vehicle group showed a tendency to revert toward a WT- or Sham-like expression pattern upon NC114 treatment. For the 0-week time point, samples for Sham, Vehicle, and NC114 groups were substituted with WT 0-week samples.

## Slide 6
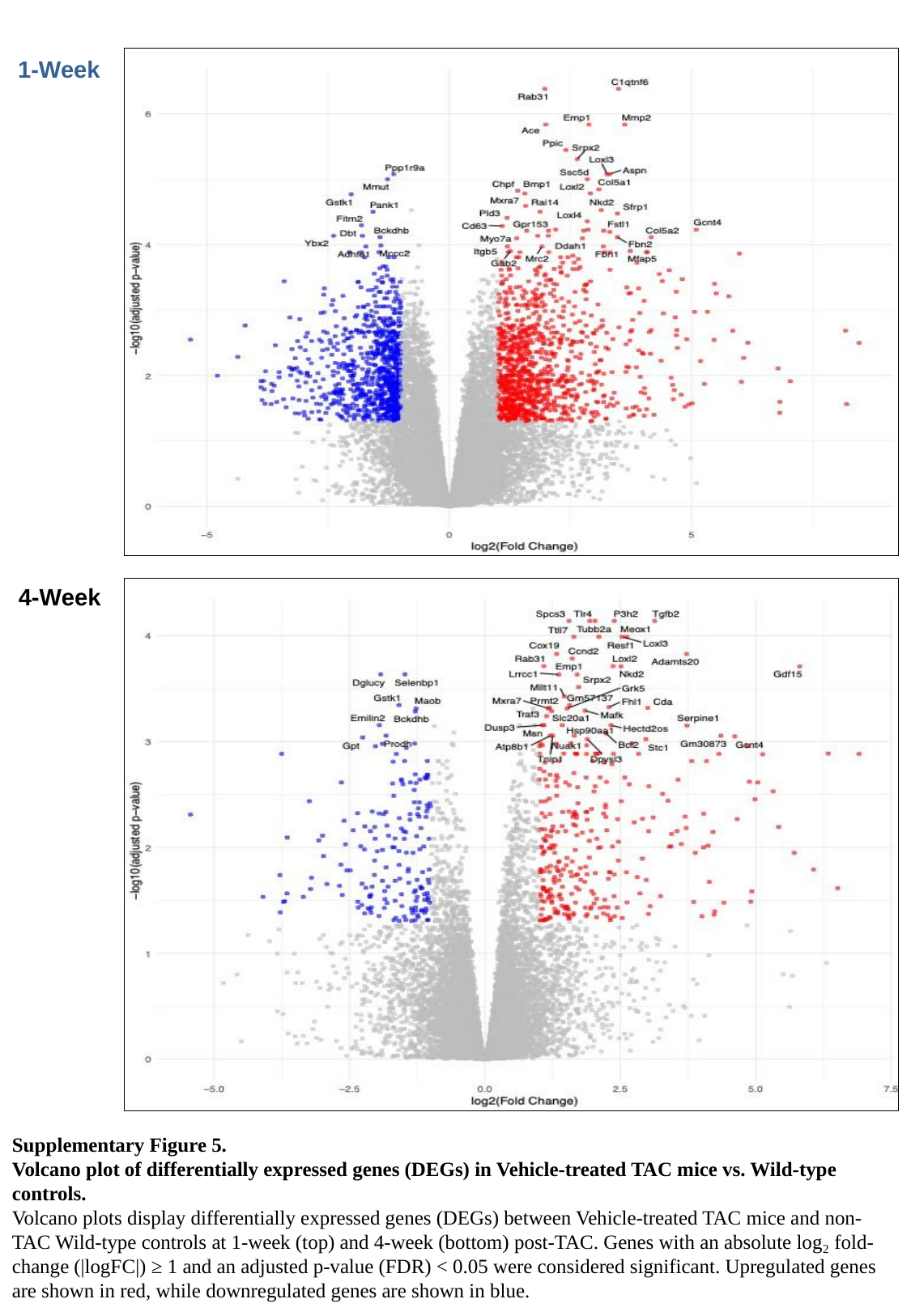

1-Week
4-Week
Supplementary Figure 5.
Volcano plot of differentially expressed genes (DEGs) in Vehicle-treated TAC mice vs. Wild-type controls.
Volcano plots display differentially expressed genes (DEGs) between Vehicle-treated TAC mice and non-TAC Wild-type controls at 1-week (top) and 4-week (bottom) post-TAC. Genes with an absolute log₂ fold-change (|logFC|) ≥ 1 and an adjusted p-value (FDR) < 0.05 were considered significant. Upregulated genes are shown in red, while downregulated genes are shown in blue.

## Slide 7
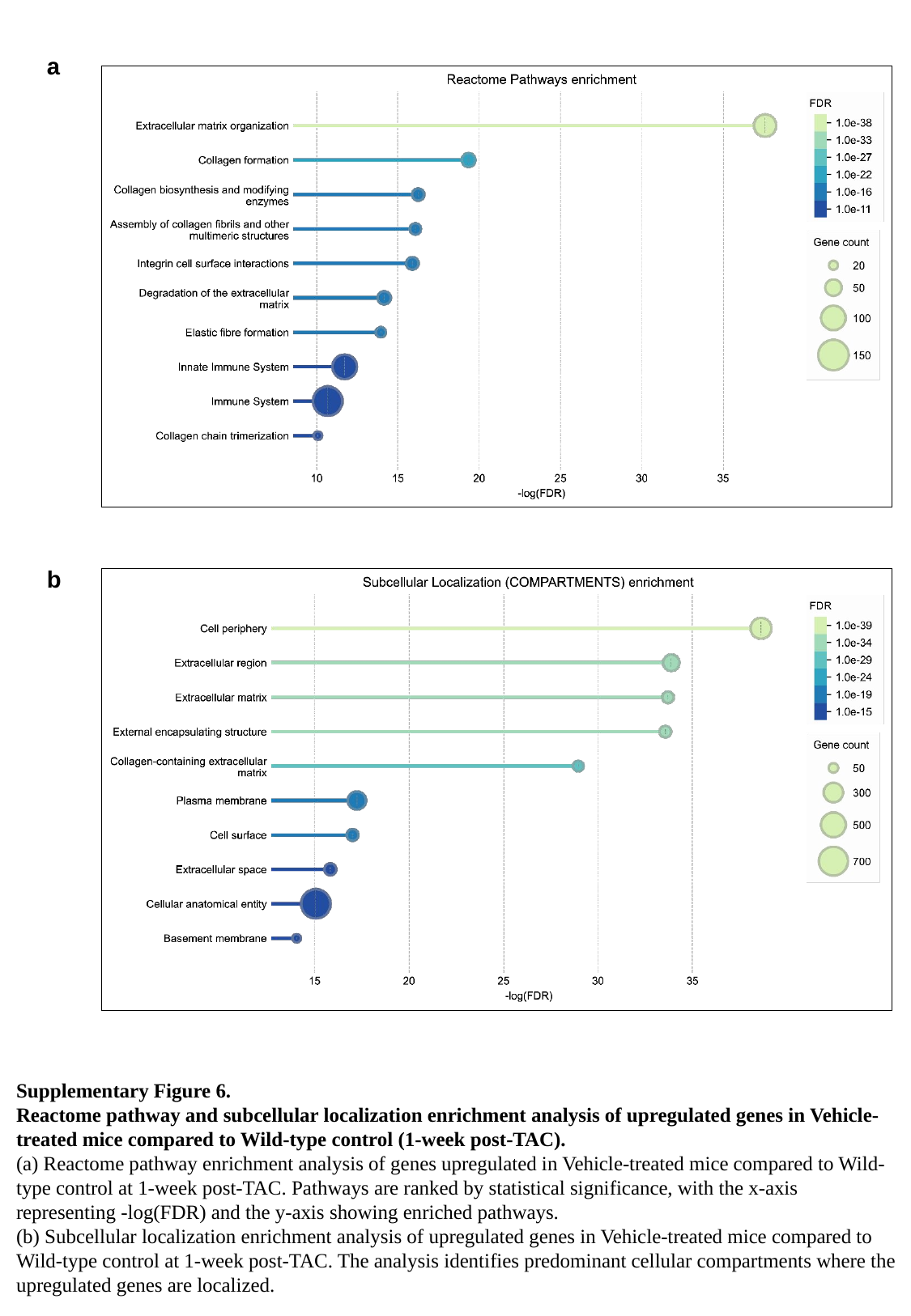

a
b
Supplementary Figure 6.
Reactome pathway and subcellular localization enrichment analysis of upregulated genes in Vehicle-treated mice compared to Wild-type control (1-week post-TAC).
(a) Reactome pathway enrichment analysis of genes upregulated in Vehicle-treated mice compared to Wild-type control at 1-week post-TAC. Pathways are ranked by statistical significance, with the x-axis representing -log(FDR) and the y-axis showing enriched pathways.
(b) Subcellular localization enrichment analysis of upregulated genes in Vehicle-treated mice compared to Wild-type control at 1-week post-TAC. The analysis identifies predominant cellular compartments where the upregulated genes are localized.

## Slide 8
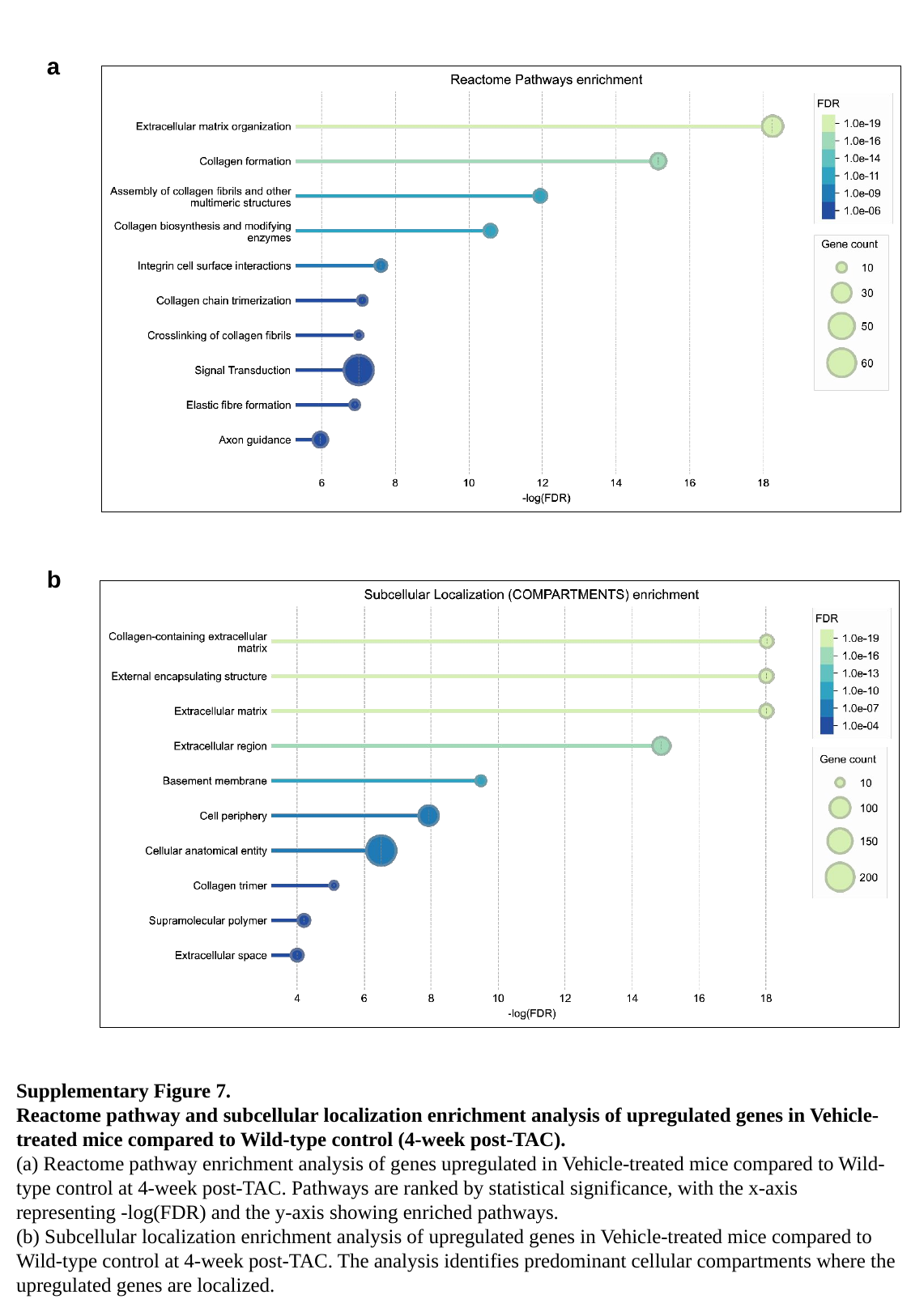

a
b
Supplementary Figure 7.
Reactome pathway and subcellular localization enrichment analysis of upregulated genes in Vehicle-treated mice compared to Wild-type control (4-week post-TAC).
(a) Reactome pathway enrichment analysis of genes upregulated in Vehicle-treated mice compared to Wild-type control at 4-week post-TAC. Pathways are ranked by statistical significance, with the x-axis representing -log(FDR) and the y-axis showing enriched pathways.
(b) Subcellular localization enrichment analysis of upregulated genes in Vehicle-treated mice compared to Wild-type control at 4-week post-TAC. The analysis identifies predominant cellular compartments where the upregulated genes are localized.

## Slide 9
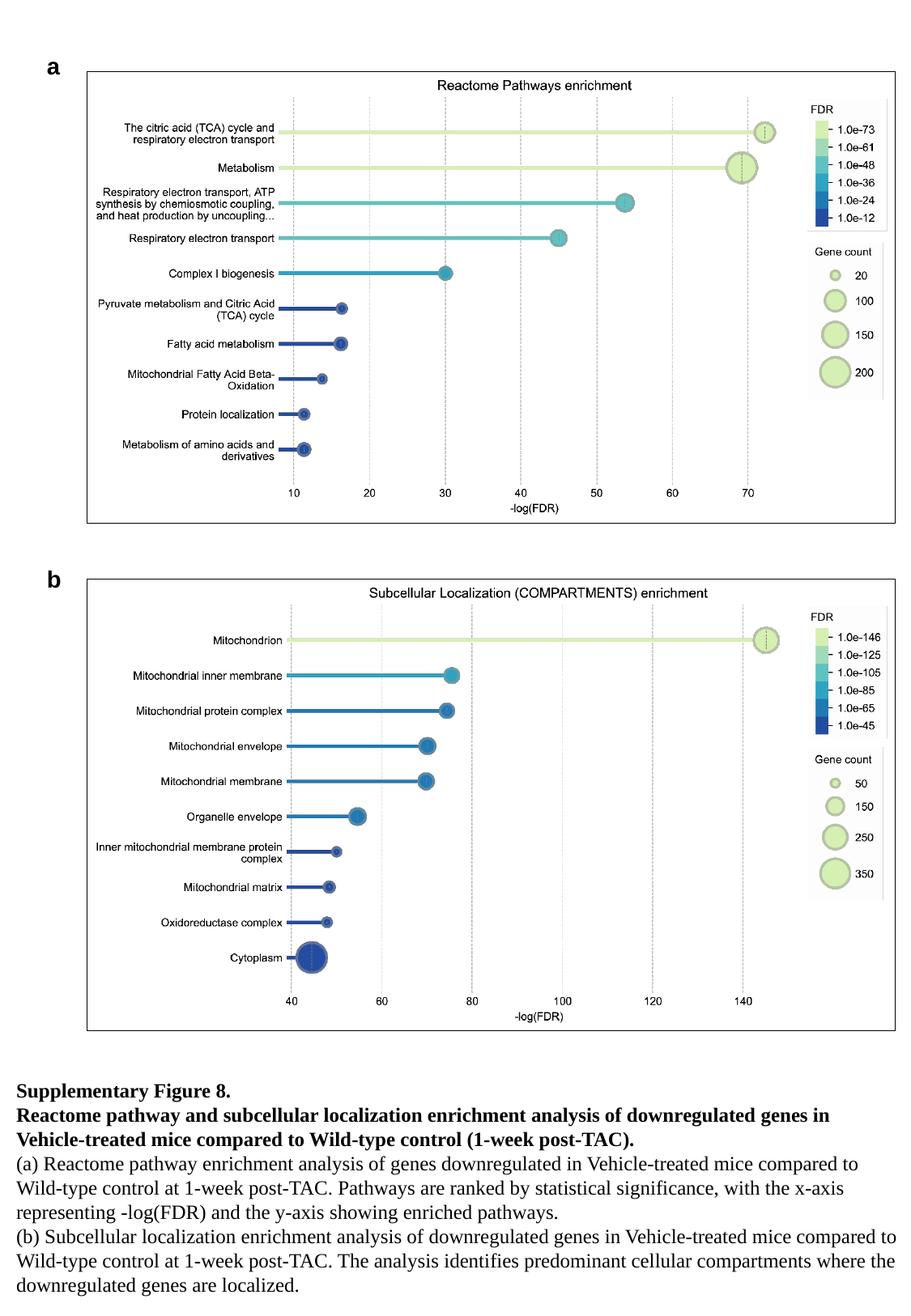

a
b
Supplementary Figure 8.
Reactome pathway and subcellular localization enrichment analysis of downregulated genes in Vehicle-treated mice compared to Wild-type control (1-week post-TAC).
(a) Reactome pathway enrichment analysis of genes downregulated in Vehicle-treated mice compared to Wild-type control at 1-week post-TAC. Pathways are ranked by statistical significance, with the x-axis representing -log(FDR) and the y-axis showing enriched pathways.
(b) Subcellular localization enrichment analysis of downregulated genes in Vehicle-treated mice compared to Wild-type control at 1-week post-TAC. The analysis identifies predominant cellular compartments where the downregulated genes are localized.

## Slide 10
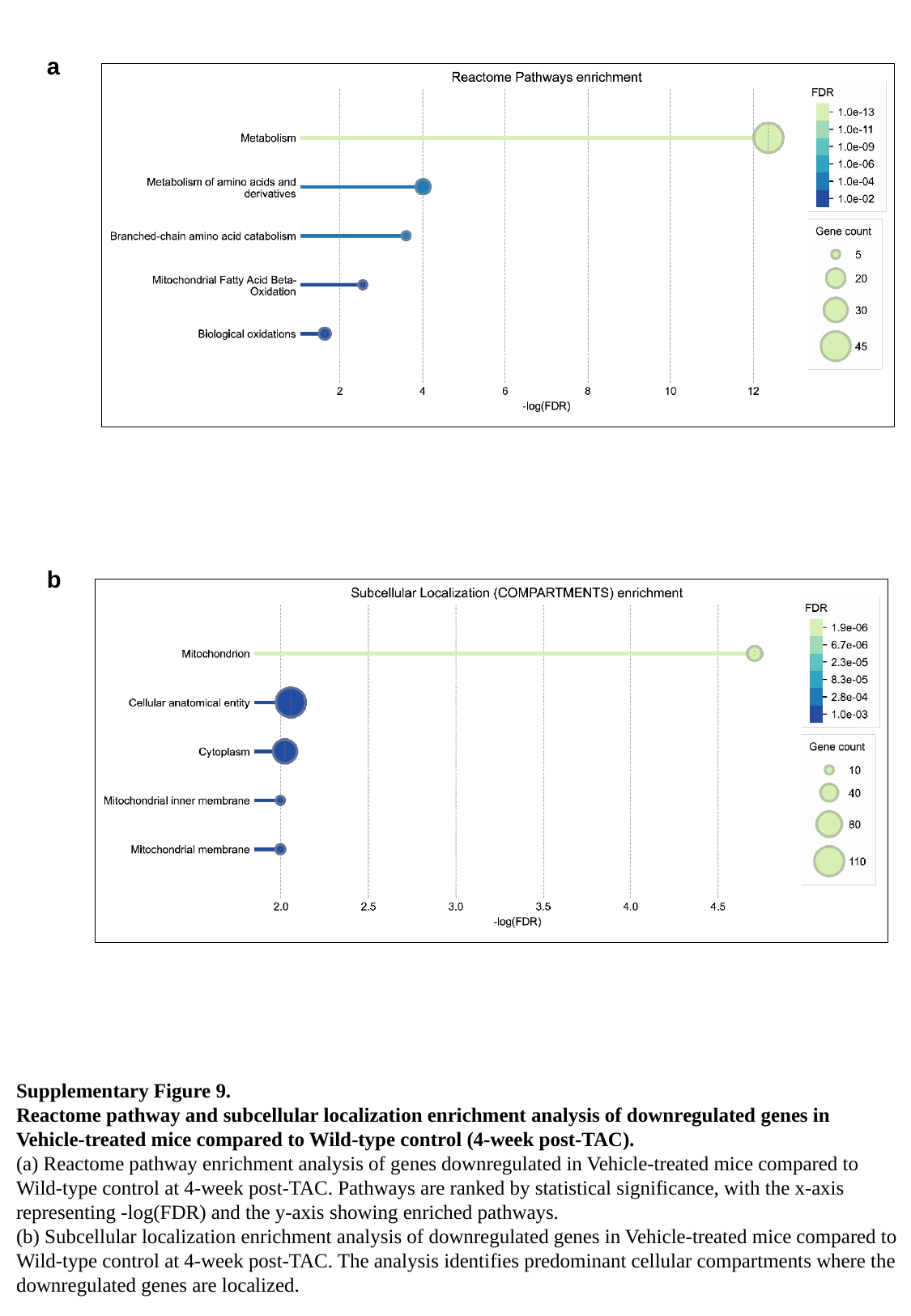

a
b
Supplementary Figure 9.
Reactome pathway and subcellular localization enrichment analysis of downregulated genes in Vehicle-treated mice compared to Wild-type control (4-week post-TAC).
(a) Reactome pathway enrichment analysis of genes downregulated in Vehicle-treated mice compared to Wild-type control at 4-week post-TAC. Pathways are ranked by statistical significance, with the x-axis representing -log(FDR) and the y-axis showing enriched pathways.
(b) Subcellular localization enrichment analysis of downregulated genes in Vehicle-treated mice compared to Wild-type control at 4-week post-TAC. The analysis identifies predominant cellular compartments where the downregulated genes are localized.

## Slide 11
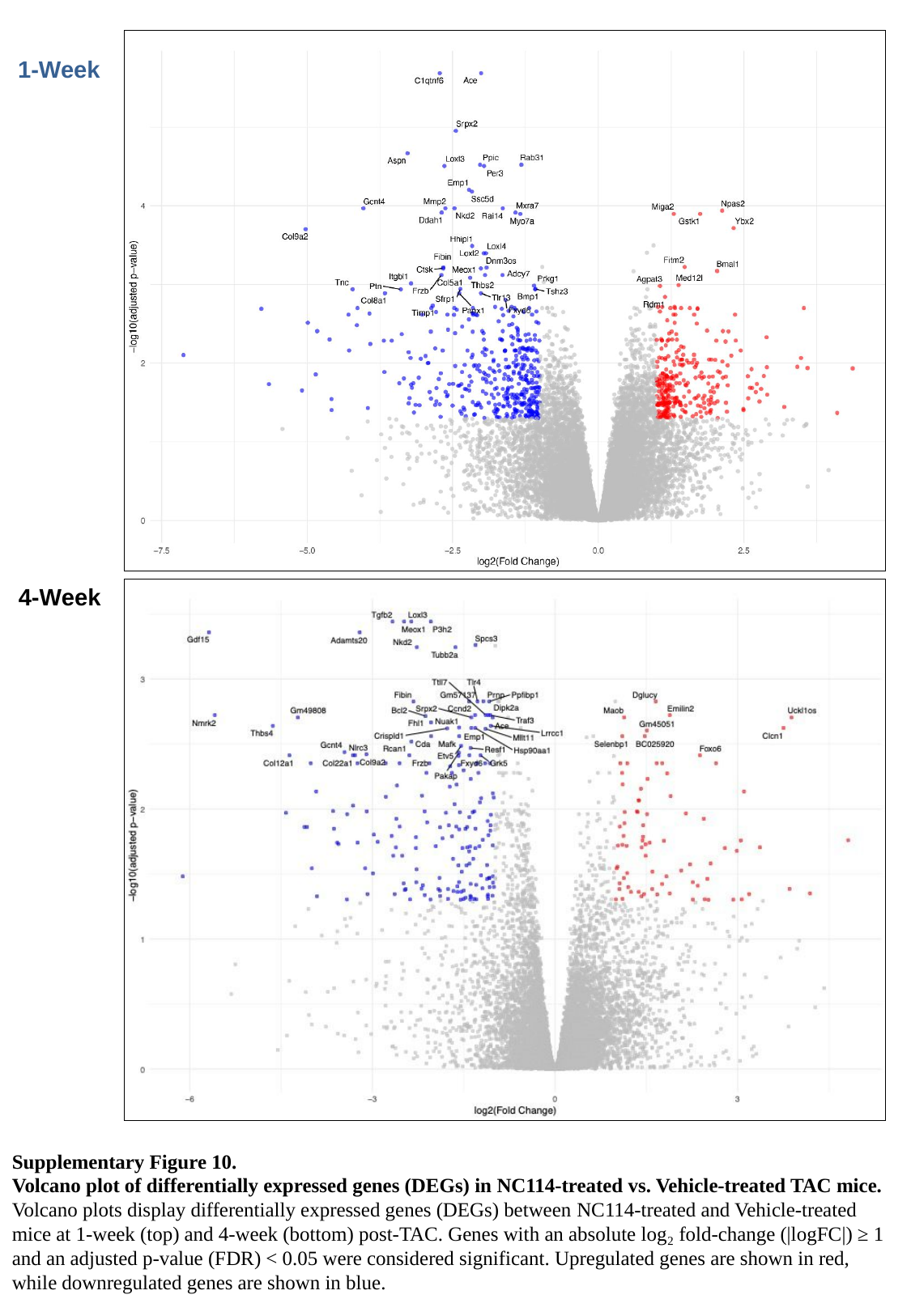

1-Week
4-Week
Supplementary Figure 10.
Volcano plot of differentially expressed genes (DEGs) in NC114-treated vs. Vehicle-treated TAC mice.
Volcano plots display differentially expressed genes (DEGs) between NC114-treated and Vehicle-treated mice at 1-week (top) and 4-week (bottom) post-TAC. Genes with an absolute log₂ fold-change (|logFC|) ≥ 1 and an adjusted p-value (FDR) < 0.05 were considered significant. Upregulated genes are shown in red, while downregulated genes are shown in blue.

## Slide 12
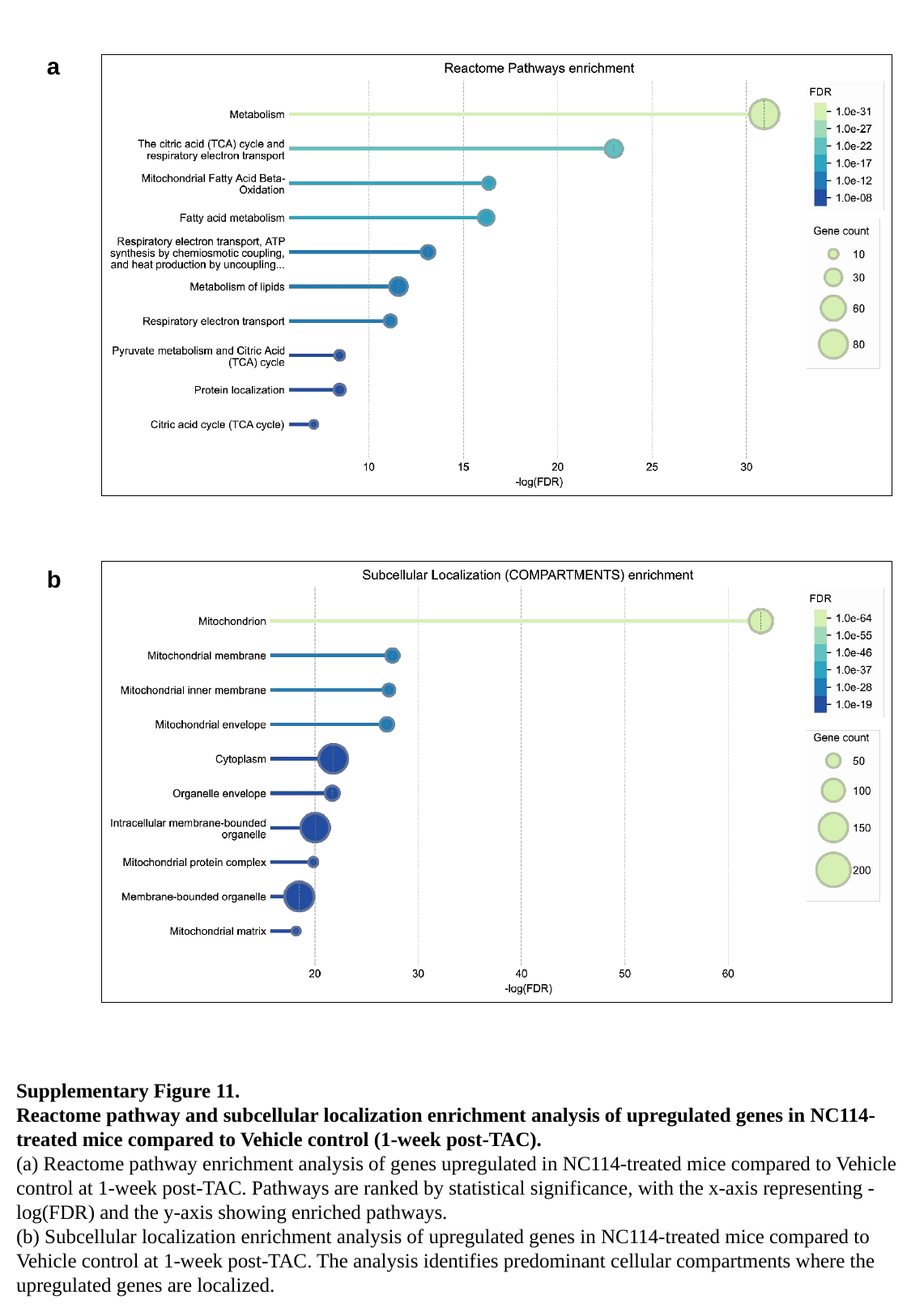

a
b
Supplementary Figure 11.
Reactome pathway and subcellular localization enrichment analysis of upregulated genes in NC114-treated mice compared to Vehicle control (1-week post-TAC).
(a) Reactome pathway enrichment analysis of genes upregulated in NC114-treated mice compared to Vehicle control at 1-week post-TAC. Pathways are ranked by statistical significance, with the x-axis representing -log(FDR) and the y-axis showing enriched pathways.
(b) Subcellular localization enrichment analysis of upregulated genes in NC114-treated mice compared to Vehicle control at 1-week post-TAC. The analysis identifies predominant cellular compartments where the upregulated genes are localized.

## Slide 13
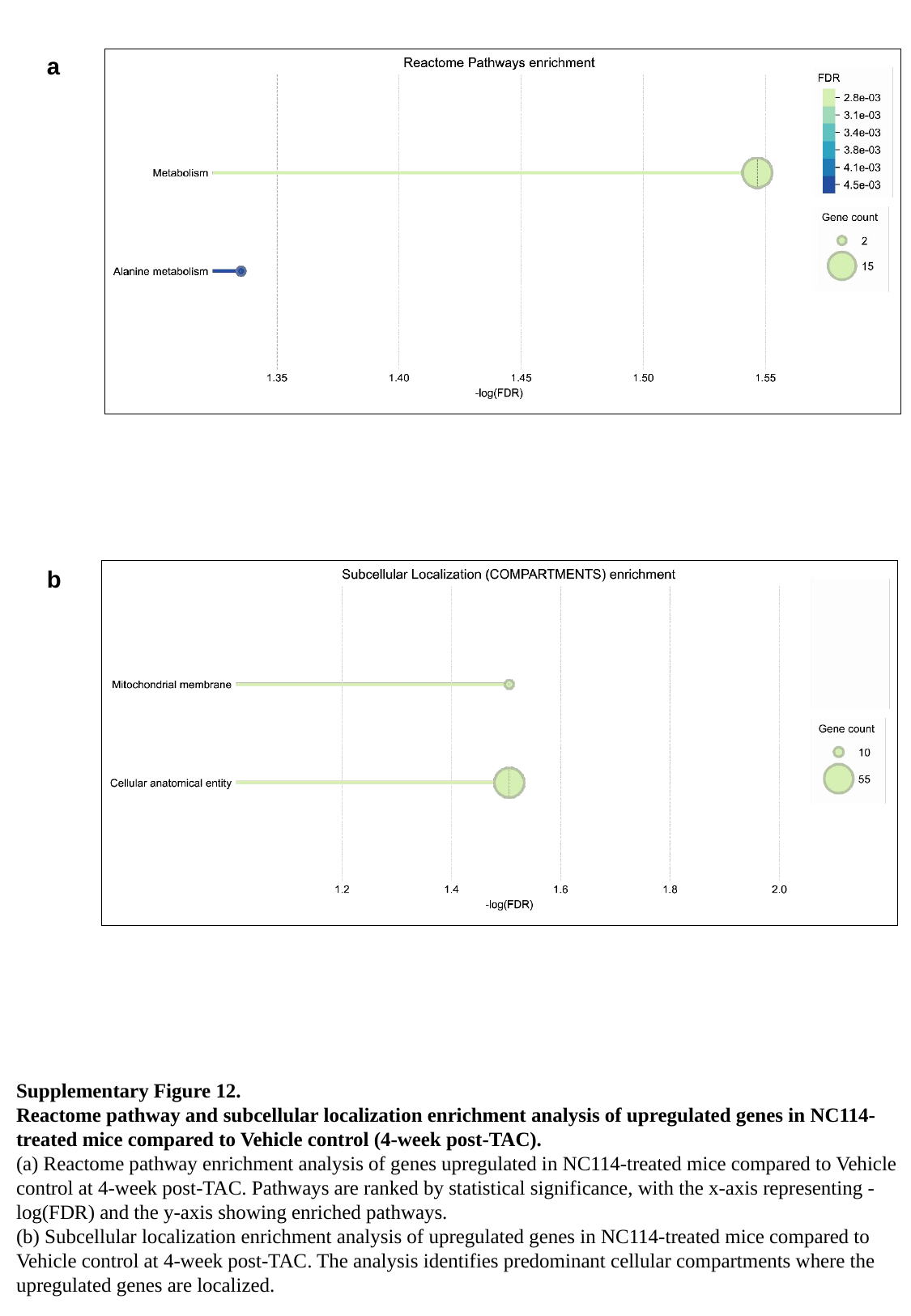

a
b
Supplementary Figure 12.
Reactome pathway and subcellular localization enrichment analysis of upregulated genes in NC114-treated mice compared to Vehicle control (4-week post-TAC).
(a) Reactome pathway enrichment analysis of genes upregulated in NC114-treated mice compared to Vehicle control at 4-week post-TAC. Pathways are ranked by statistical significance, with the x-axis representing -log(FDR) and the y-axis showing enriched pathways.
(b) Subcellular localization enrichment analysis of upregulated genes in NC114-treated mice compared to Vehicle control at 4-week post-TAC. The analysis identifies predominant cellular compartments where the upregulated genes are localized.

## Slide 14
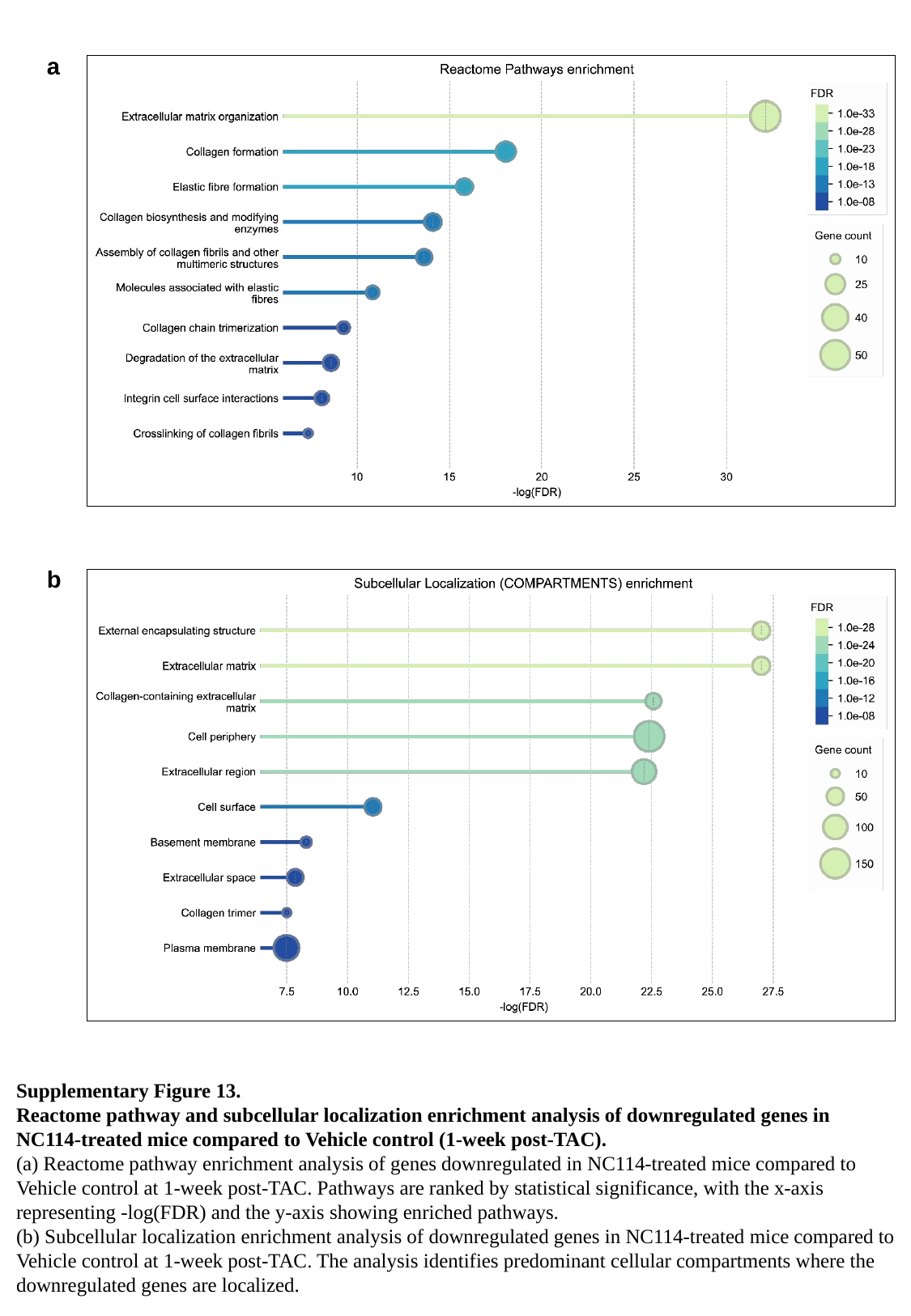

a
b
Supplementary Figure 13.
Reactome pathway and subcellular localization enrichment analysis of downregulated genes in NC114-treated mice compared to Vehicle control (1-week post-TAC).
(a) Reactome pathway enrichment analysis of genes downregulated in NC114-treated mice compared to Vehicle control at 1-week post-TAC. Pathways are ranked by statistical significance, with the x-axis representing -log(FDR) and the y-axis showing enriched pathways.
(b) Subcellular localization enrichment analysis of downregulated genes in NC114-treated mice compared to Vehicle control at 1-week post-TAC. The analysis identifies predominant cellular compartments where the downregulated genes are localized.

## Slide 15
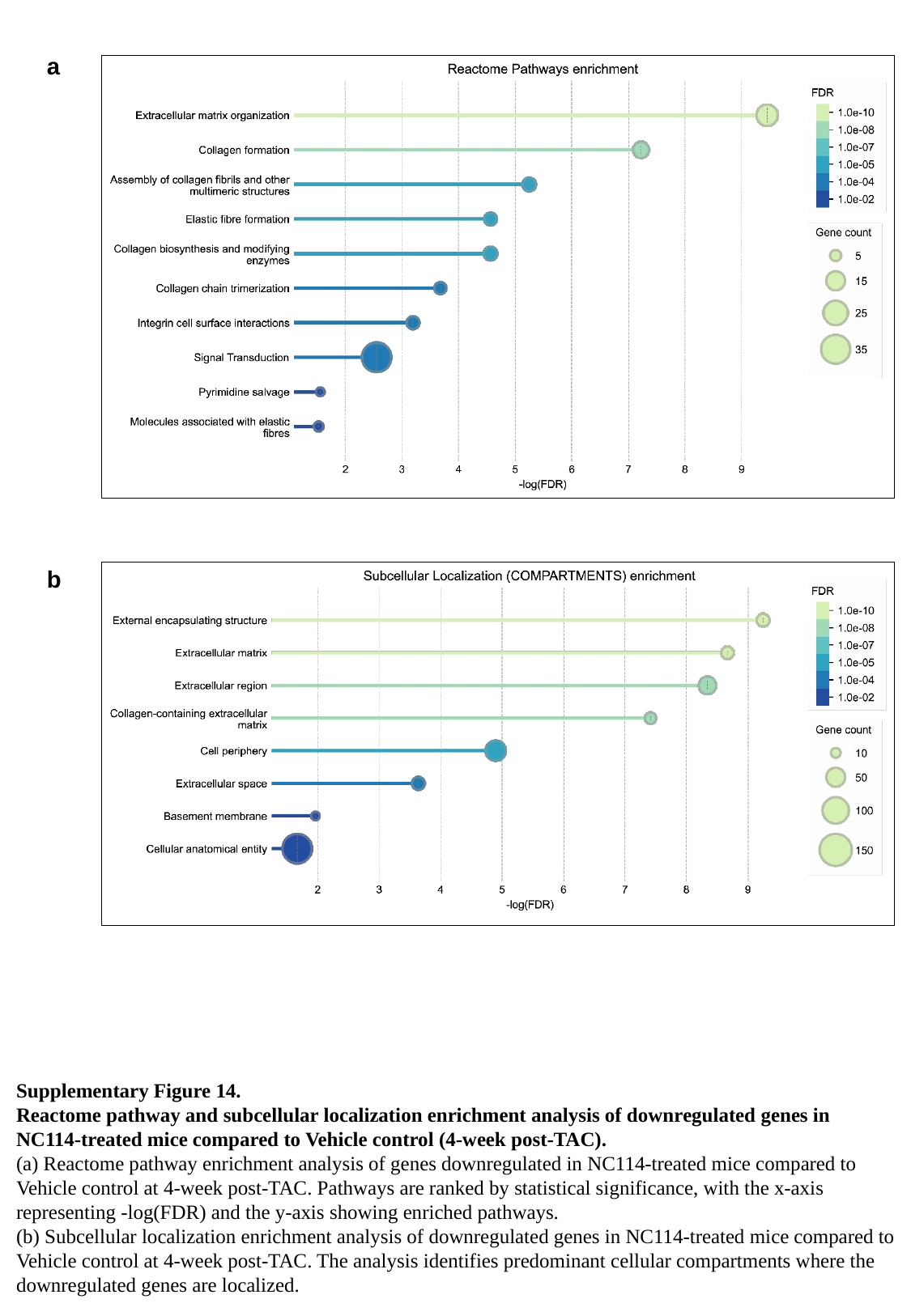

a
b
Supplementary Figure 14.
Reactome pathway and subcellular localization enrichment analysis of downregulated genes in NC114-treated mice compared to Vehicle control (4-week post-TAC).
(a) Reactome pathway enrichment analysis of genes downregulated in NC114-treated mice compared to Vehicle control at 4-week post-TAC. Pathways are ranked by statistical significance, with the x-axis representing -log(FDR) and the y-axis showing enriched pathways.
(b) Subcellular localization enrichment analysis of downregulated genes in NC114-treated mice compared to Vehicle control at 4-week post-TAC. The analysis identifies predominant cellular compartments where the downregulated genes are localized.

## Slide 16
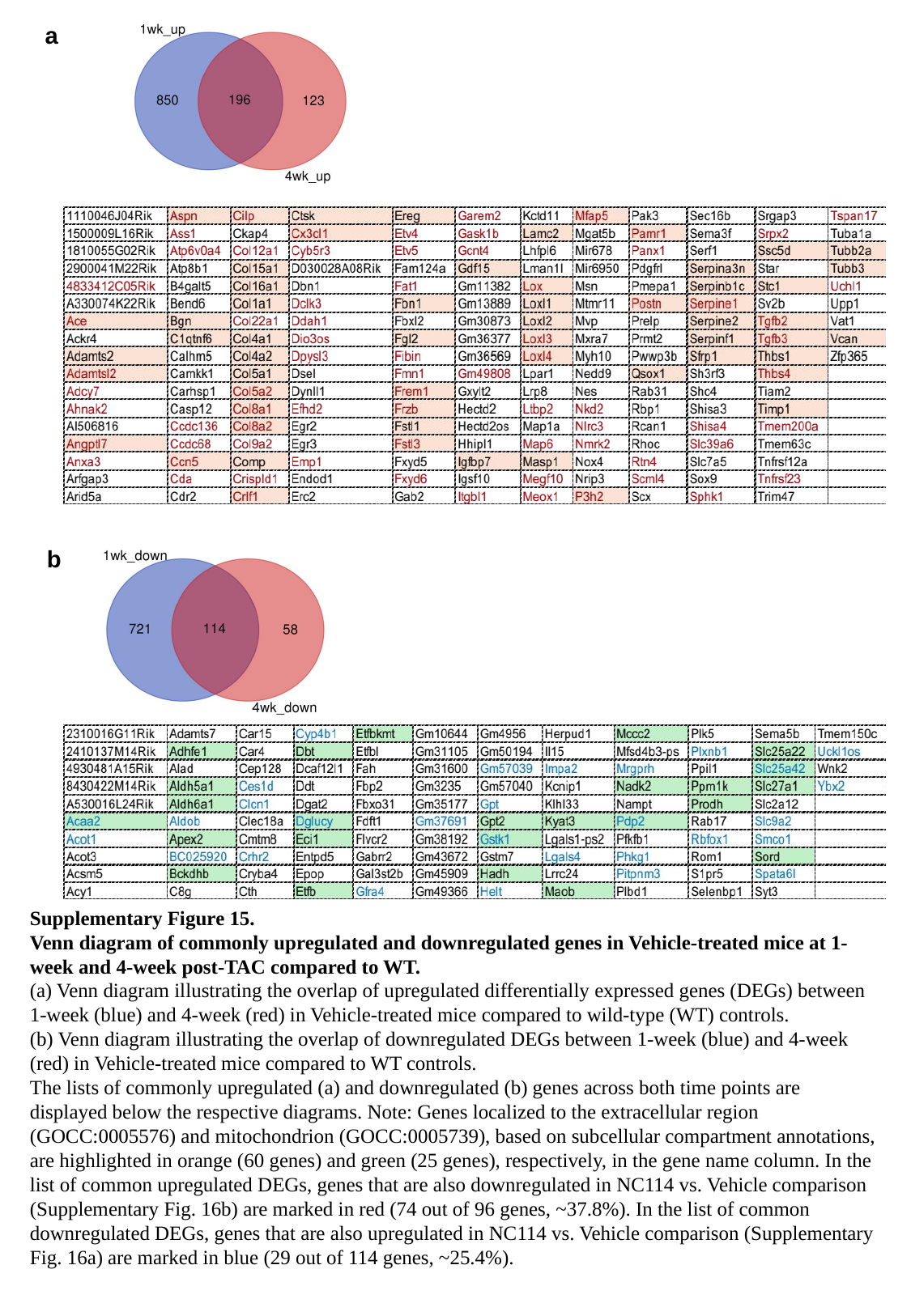

a
b
Supplementary Figure 15.
Venn diagram of commonly upregulated and downregulated genes in Vehicle-treated mice at 1-week and 4-week post-TAC compared to WT.
(a) Venn diagram illustrating the overlap of upregulated differentially expressed genes (DEGs) between 1-week (blue) and 4-week (red) in Vehicle-treated mice compared to wild-type (WT) controls.
(b) Venn diagram illustrating the overlap of downregulated DEGs between 1-week (blue) and 4-week (red) in Vehicle-treated mice compared to WT controls.
The lists of commonly upregulated (a) and downregulated (b) genes across both time points are displayed below the respective diagrams. Note: Genes localized to the extracellular region (GOCC:0005576) and mitochondrion (GOCC:0005739), based on subcellular compartment annotations, are highlighted in orange (60 genes) and green (25 genes), respectively, in the gene name column. In the list of common upregulated DEGs, genes that are also downregulated in NC114 vs. Vehicle comparison (Supplementary Fig. 16b) are marked in red (74 out of 96 genes, ~37.8%). In the list of common downregulated DEGs, genes that are also upregulated in NC114 vs. Vehicle comparison (Supplementary Fig. 16a) are marked in blue (29 out of 114 genes, ~25.4%).

## Slide 17
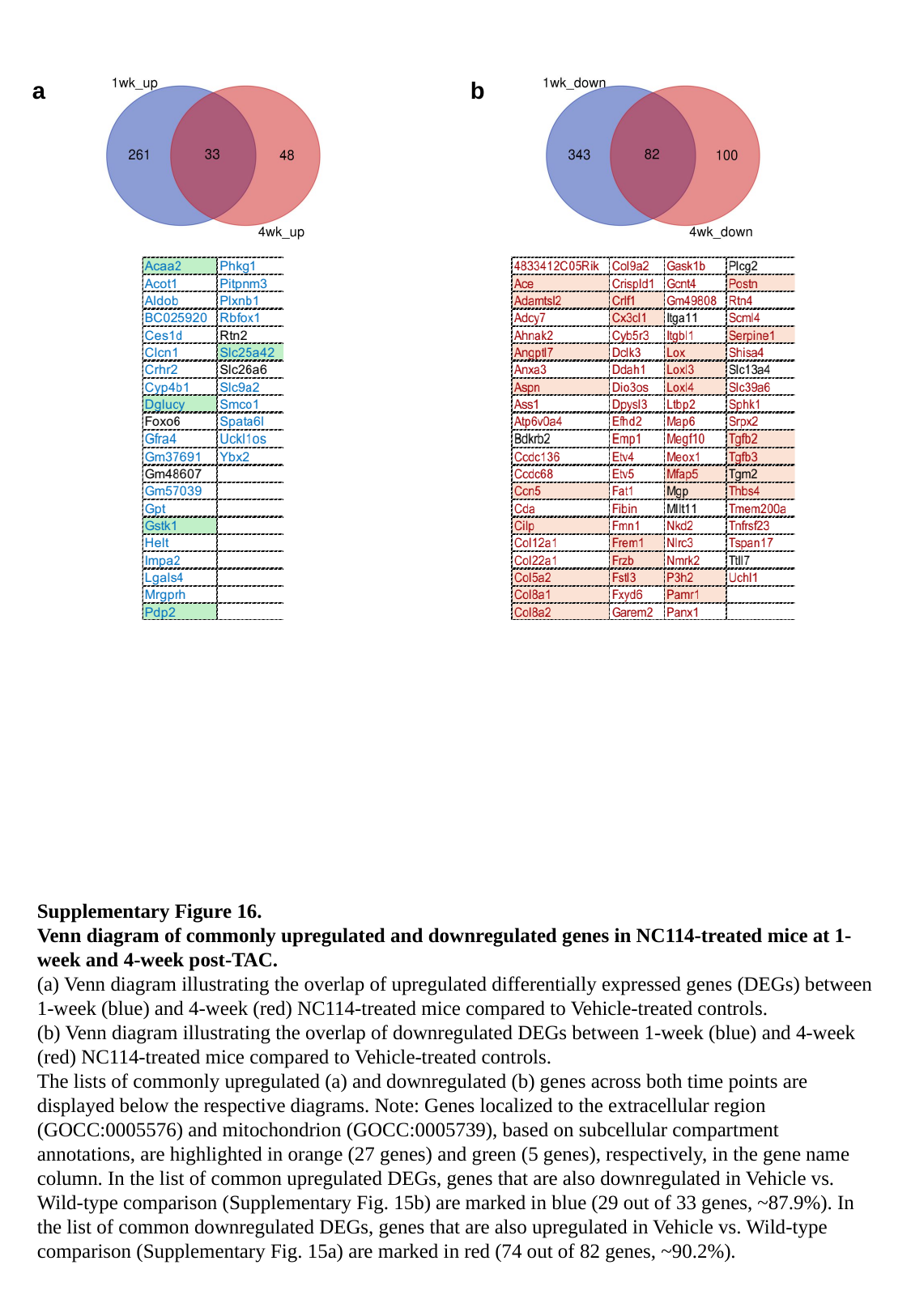

b
a
Supplementary Figure 16.
Venn diagram of commonly upregulated and downregulated genes in NC114-treated mice at 1-week and 4-week post-TAC.
(a) Venn diagram illustrating the overlap of upregulated differentially expressed genes (DEGs) between 1-week (blue) and 4-week (red) NC114-treated mice compared to Vehicle-treated controls.
(b) Venn diagram illustrating the overlap of downregulated DEGs between 1-week (blue) and 4-week (red) NC114-treated mice compared to Vehicle-treated controls.
The lists of commonly upregulated (a) and downregulated (b) genes across both time points are displayed below the respective diagrams. Note: Genes localized to the extracellular region (GOCC:0005576) and mitochondrion (GOCC:0005739), based on subcellular compartment annotations, are highlighted in orange (27 genes) and green (5 genes), respectively, in the gene name column. In the list of common upregulated DEGs, genes that are also downregulated in Vehicle vs. Wild-type comparison (Supplementary Fig. 15b) are marked in blue (29 out of 33 genes, ~87.9%). In the list of common downregulated DEGs, genes that are also upregulated in Vehicle vs. Wild-type comparison (Supplementary Fig. 15a) are marked in red (74 out of 82 genes, ~90.2%).

## Slide 18
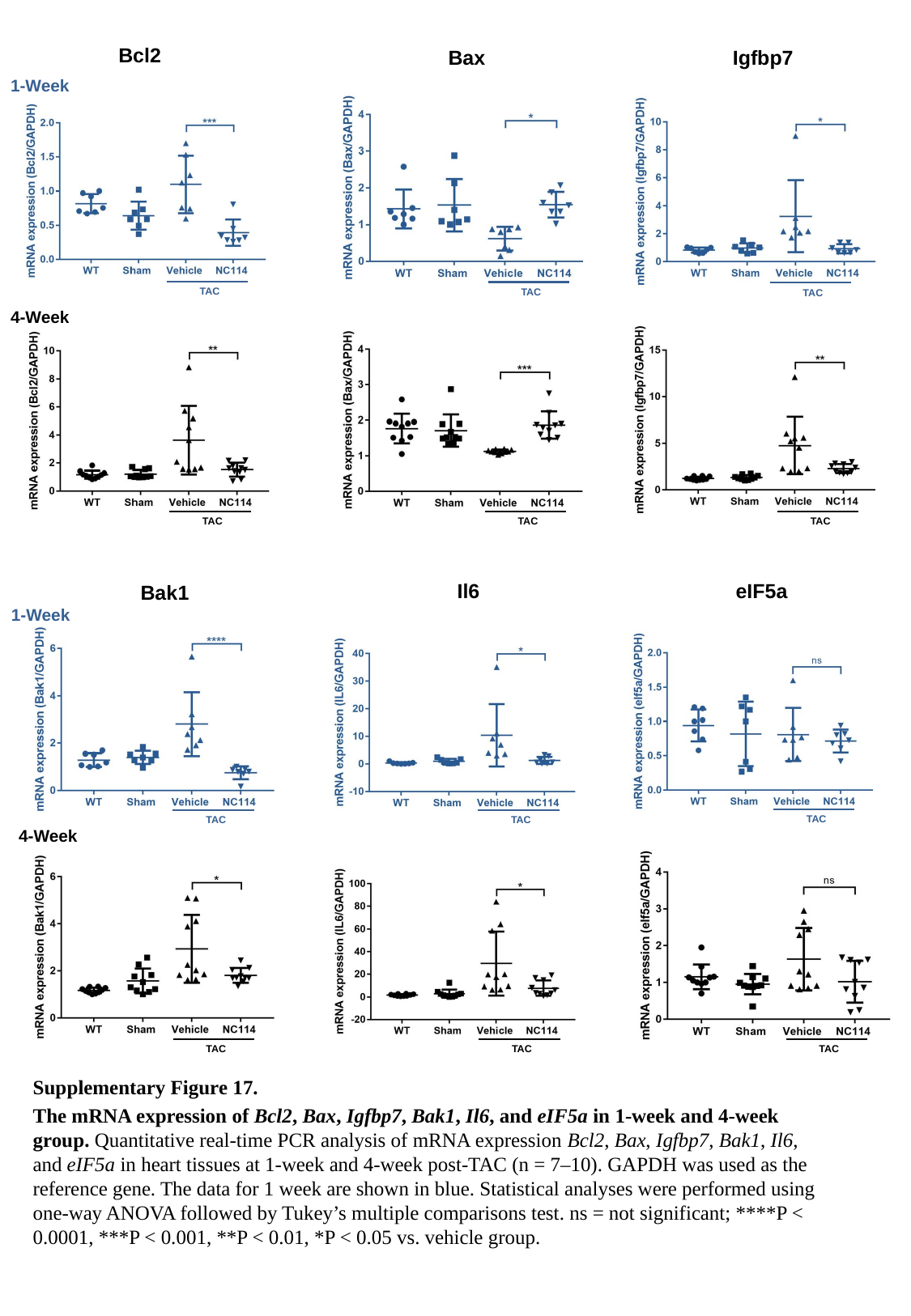

Bcl2
Igfbp7
Bax
1-Week
TAC
TAC
TAC
4-Week
TAC
TAC
TAC
Il6
eIF5a
Bak1
1-Week
TAC
TAC
TAC
4-Week
TAC
TAC
TAC
Supplementary Figure 17.
The mRNA expression of Bcl2, Bax, Igfbp7, Bak1, Il6, and eIF5a in 1-week and 4-week group. Quantitative real-time PCR analysis of mRNA expression Bcl2, Bax, Igfbp7, Bak1, Il6, and eIF5a in heart tissues at 1-week and 4-week post-TAC (n = 7–10). GAPDH was used as the reference gene. The data for 1 week are shown in blue. Statistical analyses were performed using one-way ANOVA followed by Tukey’s multiple comparisons test. ns = not significant; ****P < 0.0001, ***P < 0.001, **P < 0.01, *P < 0.05 vs. vehicle group.

## Slide 19
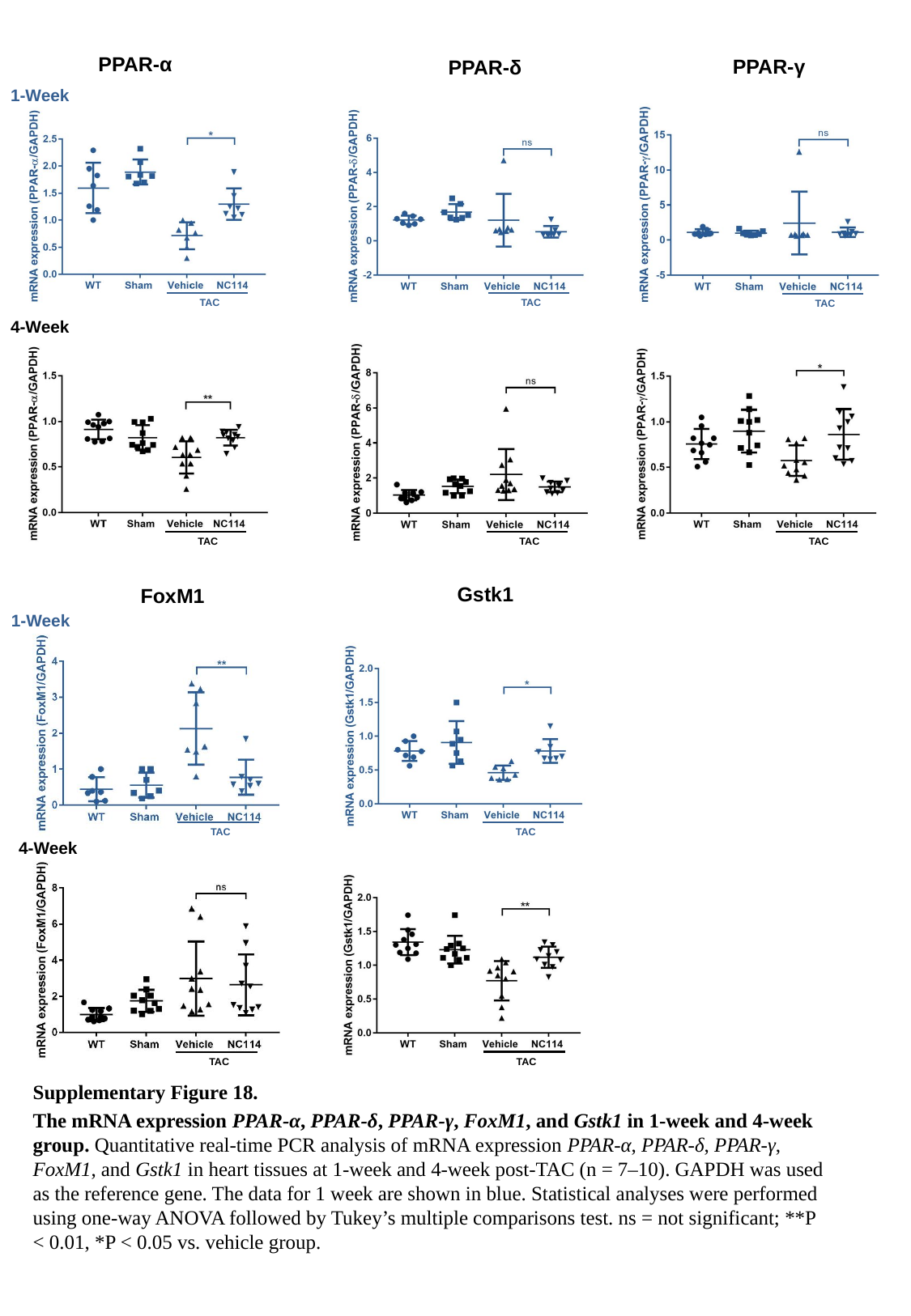

PPAR-α
PPAR-γ
PPAR-δ
1-Week
TAC
TAC
TAC
4-Week
TAC
TAC
TAC
Gstk1
FoxM1
1-Week
TAC
TAC
4-Week
TAC
TAC
Supplementary Figure 18.
The mRNA expression PPAR-α, PPAR-δ, PPAR-γ, FoxM1, and Gstk1 in 1-week and 4-week group. Quantitative real-time PCR analysis of mRNA expression PPAR-α, PPAR-δ, PPAR-γ, FoxM1, and Gstk1 in heart tissues at 1-week and 4-week post-TAC (n = 7–10). GAPDH was used as the reference gene. The data for 1 week are shown in blue. Statistical analyses were performed using one-way ANOVA followed by Tukey’s multiple comparisons test. ns = not significant; **P < 0.01, *P < 0.05 vs. vehicle group.

## Slide 20
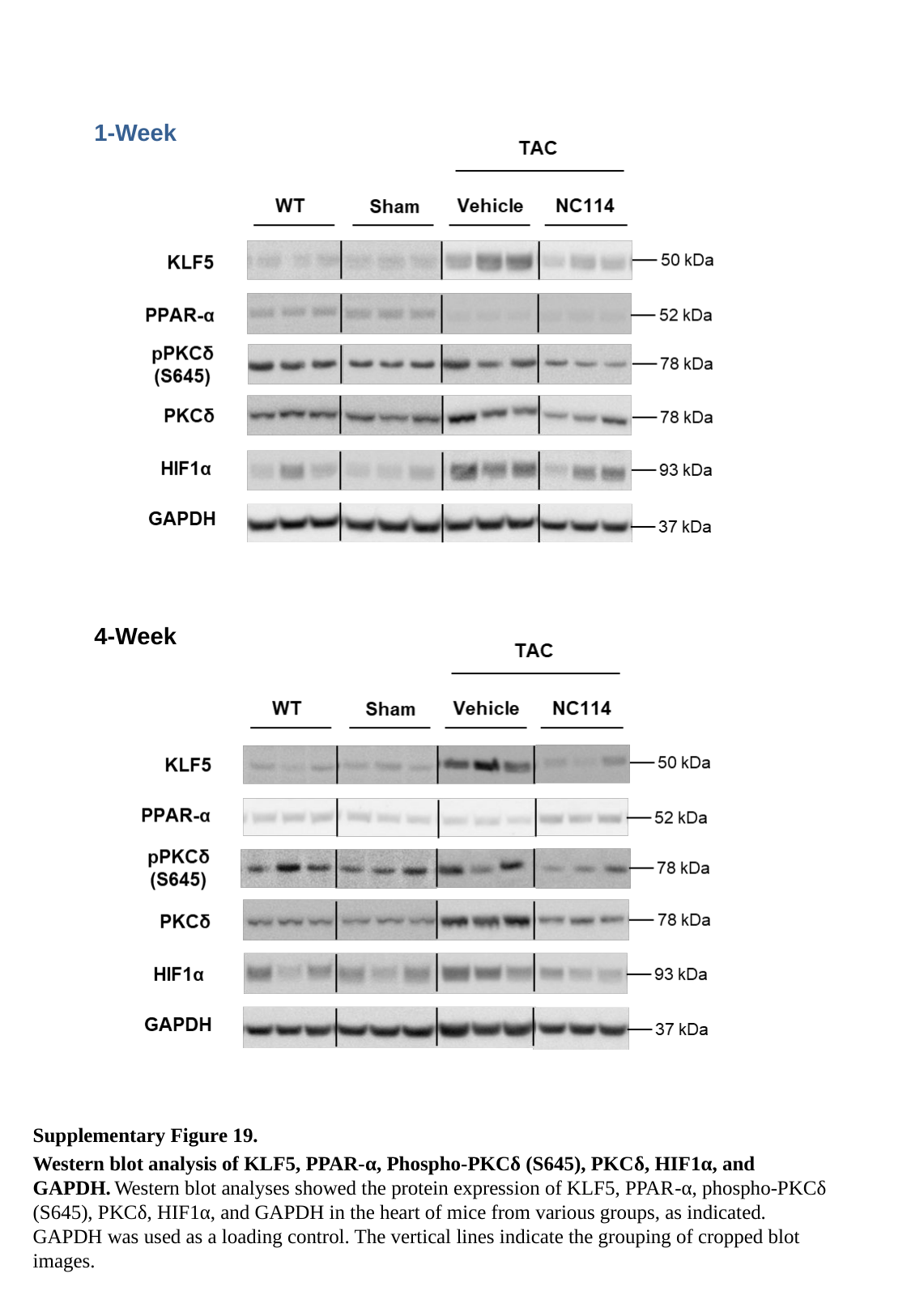

1-Week
4-Week
Supplementary Figure 19.
Western blot analysis of KLF5, PPAR-α, Phospho-PKCδ (S645), PKCδ, HIF1α, and GAPDH. Western blot analyses showed the protein expression of KLF5, PPAR-α, phospho-PKCδ (S645), PKCδ, HIF1α, and GAPDH in the heart of mice from various groups, as indicated. GAPDH was used as a loading control. The vertical lines indicate the grouping of cropped blot images.

## Slide 21
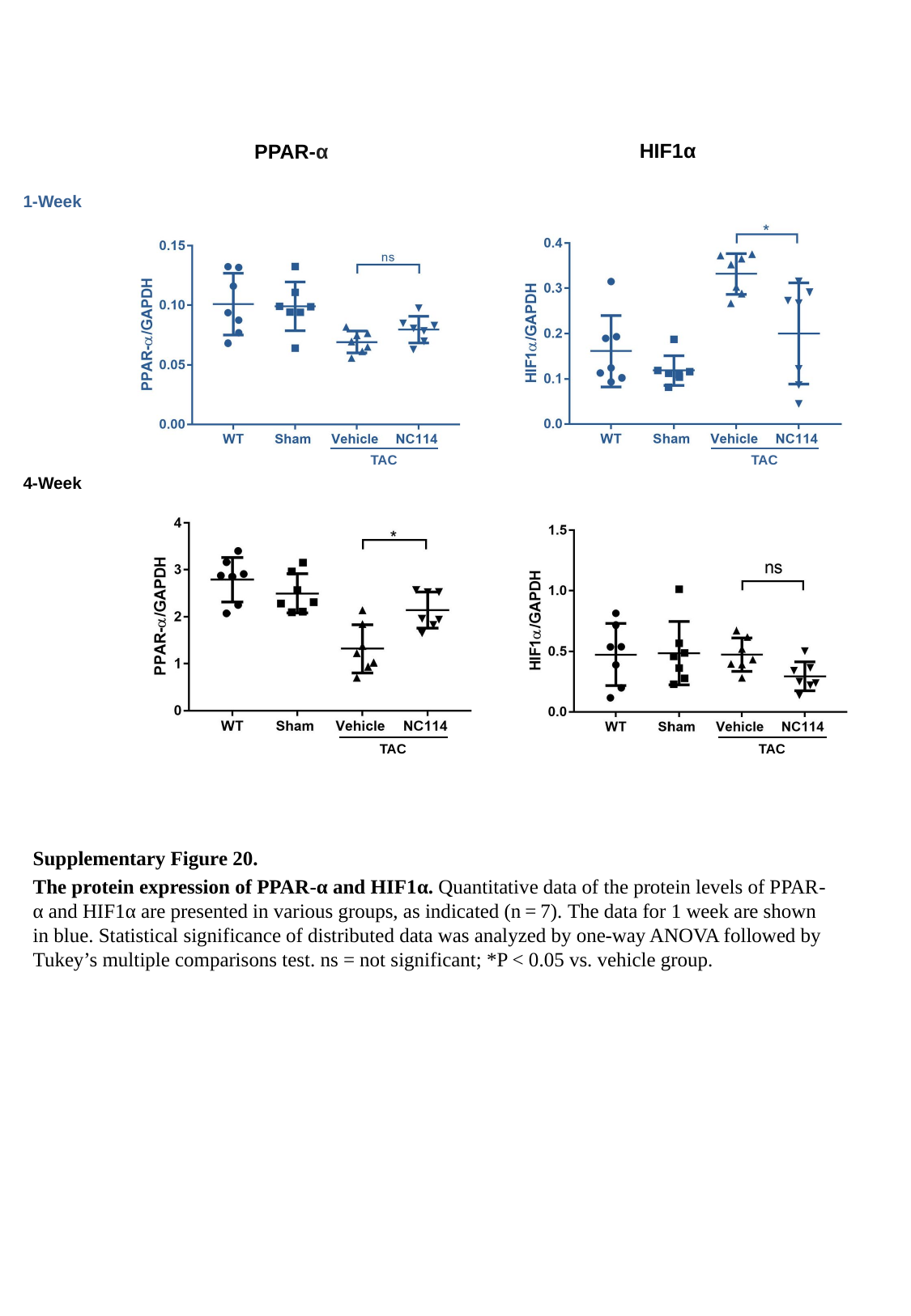

HIF1α
PPAR-α
1-Week
TAC
TAC
4-Week
TAC
TAC
Supplementary Figure 20.
The protein expression of PPAR-α and HIF1α. Quantitative data of the protein levels of PPAR-α and HIF1α are presented in various groups, as indicated (n = 7). The data for 1 week are shown in blue. Statistical significance of distributed data was analyzed by one-way ANOVA followed by Tukey’s multiple comparisons test. ns = not significant; *P < 0.05 vs. vehicle group.

## Slide 22
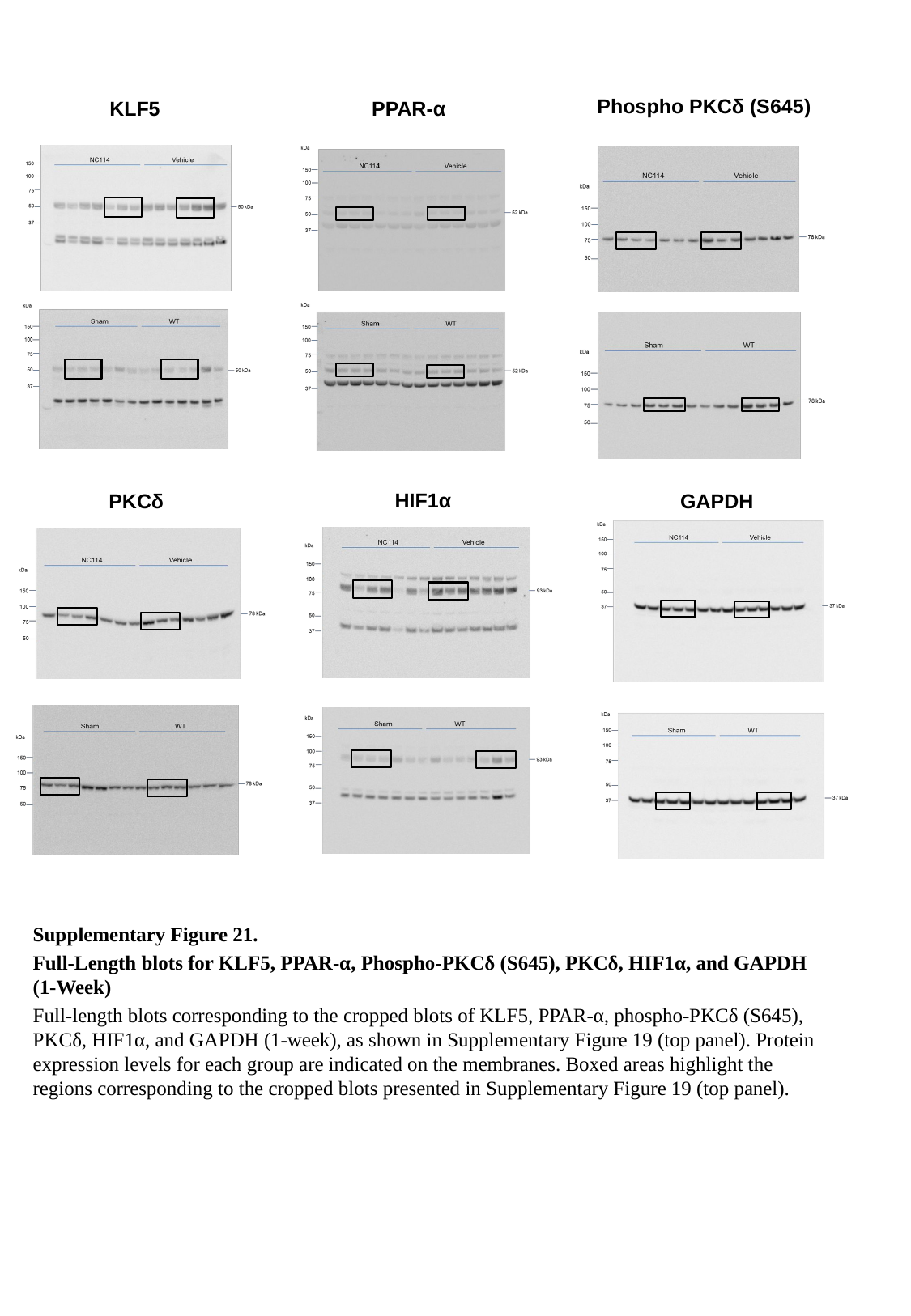

Phospho PKCδ (S645)
KLF5
PPAR-α
HIF1α
PKCδ
GAPDH
Supplementary Figure 21.
Full-Length blots for KLF5, PPAR-α, Phospho-PKCδ (S645), PKCδ, HIF1α, and GAPDH (1-Week)
Full-length blots corresponding to the cropped blots of KLF5, PPAR-α, phospho-PKCδ (S645), PKCδ, HIF1α, and GAPDH (1-week), as shown in Supplementary Figure 19 (top panel). Protein expression levels for each group are indicated on the membranes. Boxed areas highlight the regions corresponding to the cropped blots presented in Supplementary Figure 19 (top panel).

## Slide 23
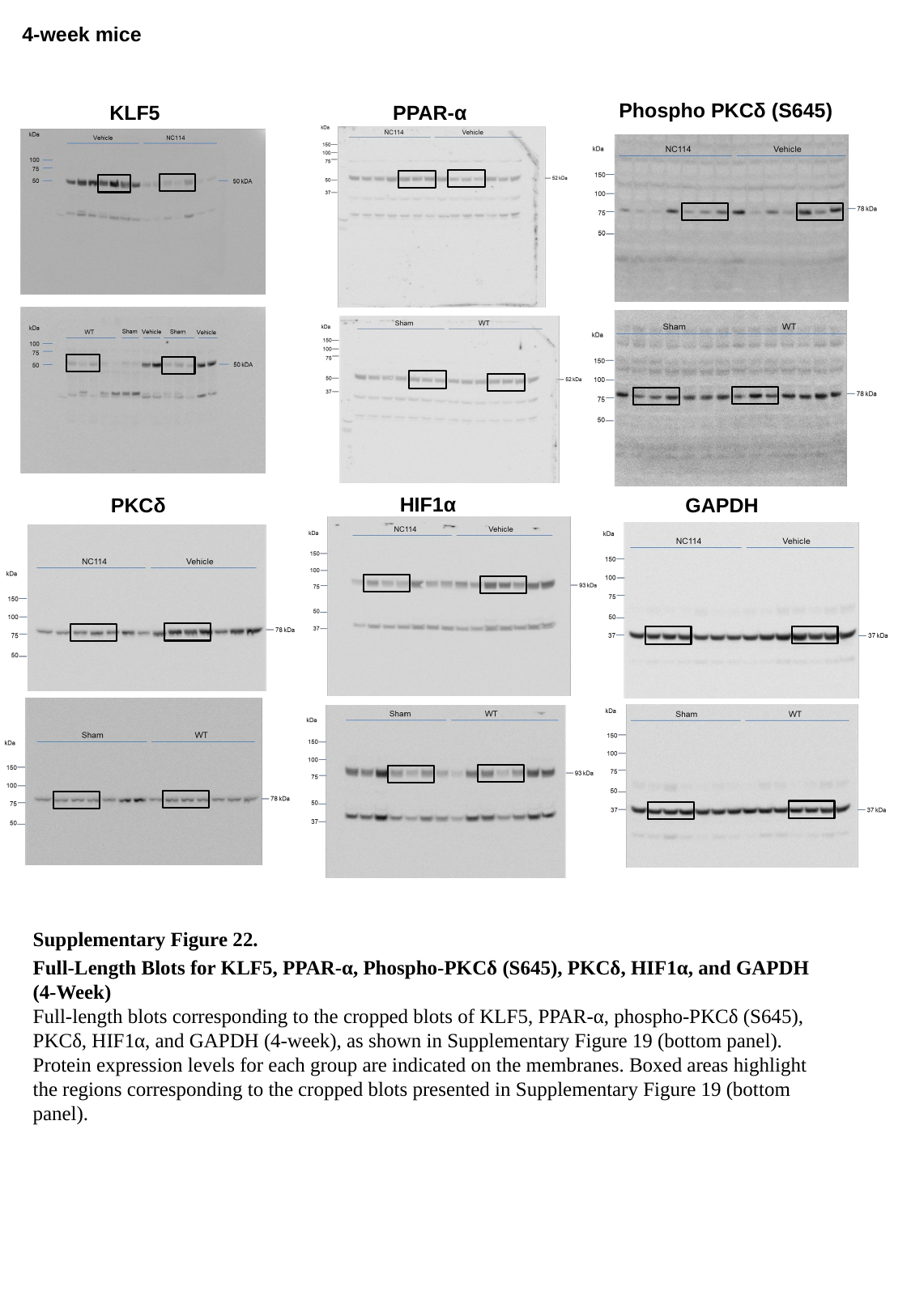

4-week mice
Phospho PKCδ (S645)
KLF5
PPAR-α
HIF1α
PKCδ
GAPDH
Supplementary Figure 22.
Full-Length Blots for KLF5, PPAR-α, Phospho-PKCδ (S645), PKCδ, HIF1α, and GAPDH (4-Week)
Full-length blots corresponding to the cropped blots of KLF5, PPAR-α, phospho-PKCδ (S645), PKCδ, HIF1α, and GAPDH (4-week), as shown in Supplementary Figure 19 (bottom panel). Protein expression levels for each group are indicated on the membranes. Boxed areas highlight the regions corresponding to the cropped blots presented in Supplementary Figure 19 (bottom panel).

## Slide 24
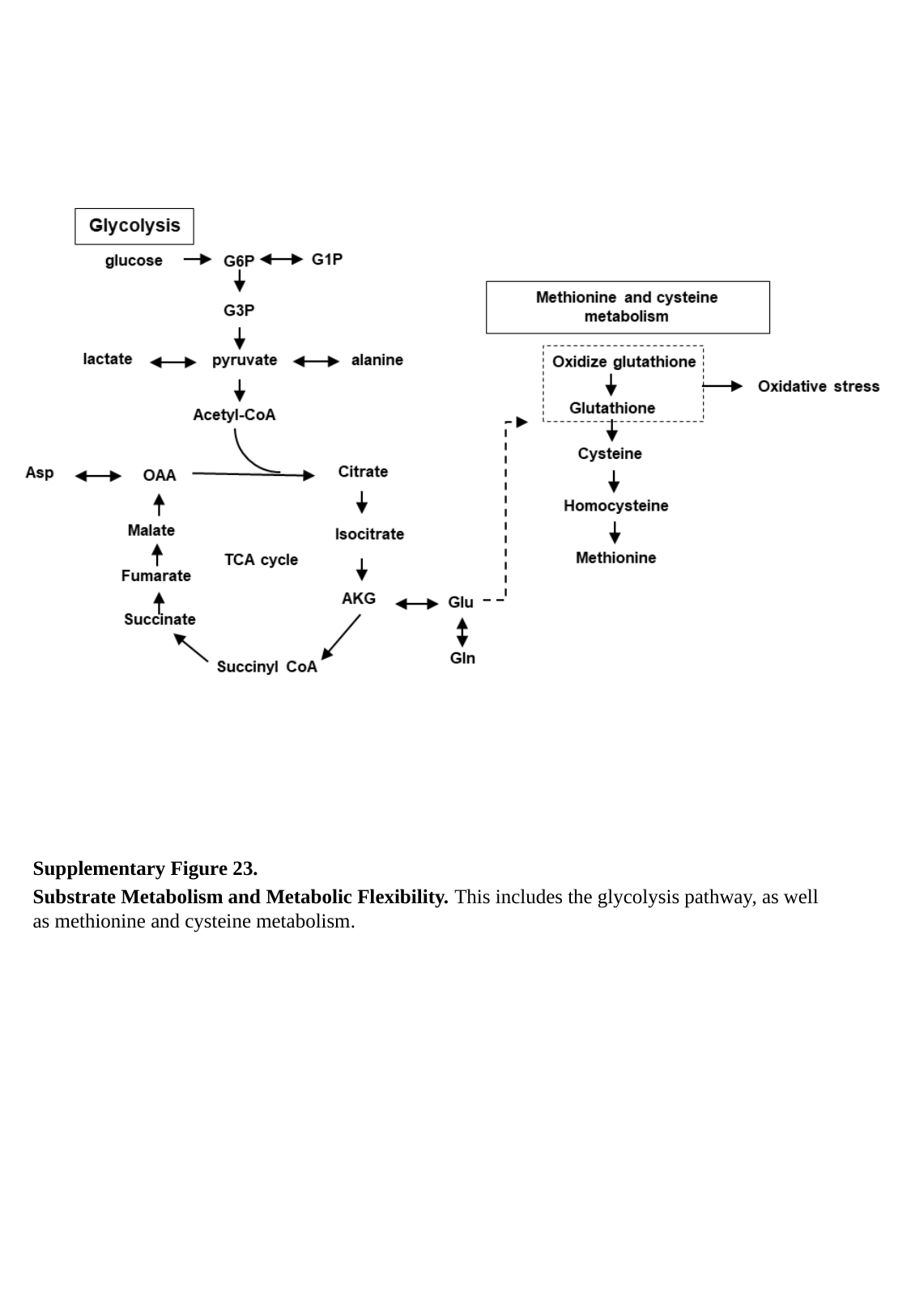

Supplementary Figure 23.
Substrate Metabolism and Metabolic Flexibility. This includes the glycolysis pathway, as well as methionine and cysteine metabolism.

## Slide 25
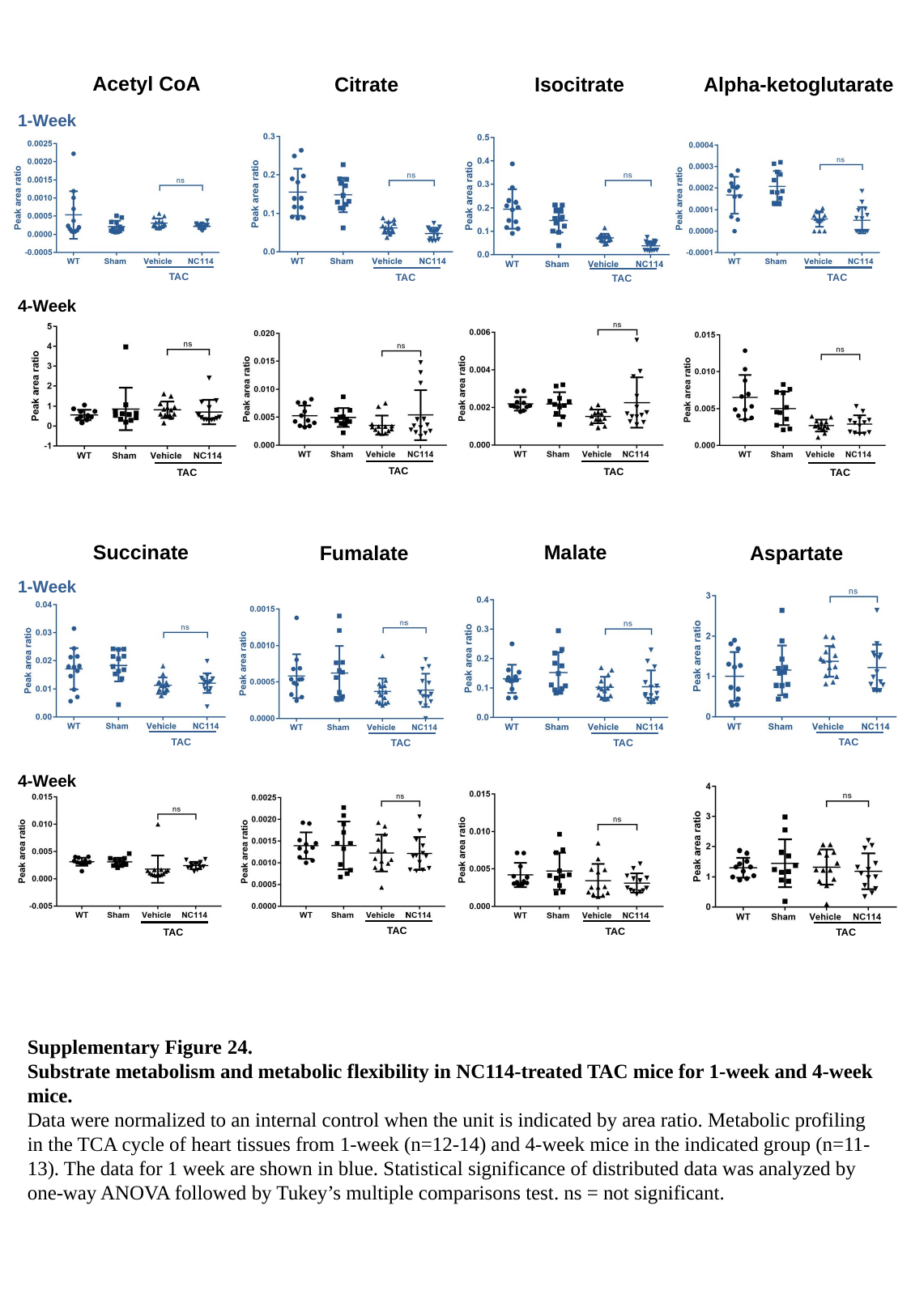

Acetyl CoA
Isocitrate
Alpha-ketoglutarate
Citrate
1-Week
TAC
TAC
TAC
TAC
4-Week
TAC
TAC
TAC
TAC
Malate
Succinate
Aspartate
Fumalate
1-Week
TAC
TAC
TAC
TAC
4-Week
TAC
TAC
TAC
TAC
Supplementary Figure 24.
Substrate metabolism and metabolic flexibility in NC114-treated TAC mice for 1-week and 4-week mice.
Data were normalized to an internal control when the unit is indicated by area ratio. Metabolic profiling in the TCA cycle of heart tissues from 1-week (n=12-14) and 4-week mice in the indicated group (n=11-13). The data for 1 week are shown in blue. Statistical significance of distributed data was analyzed by one-way ANOVA followed by Tukey’s multiple comparisons test. ns = not significant.

## Slide 26
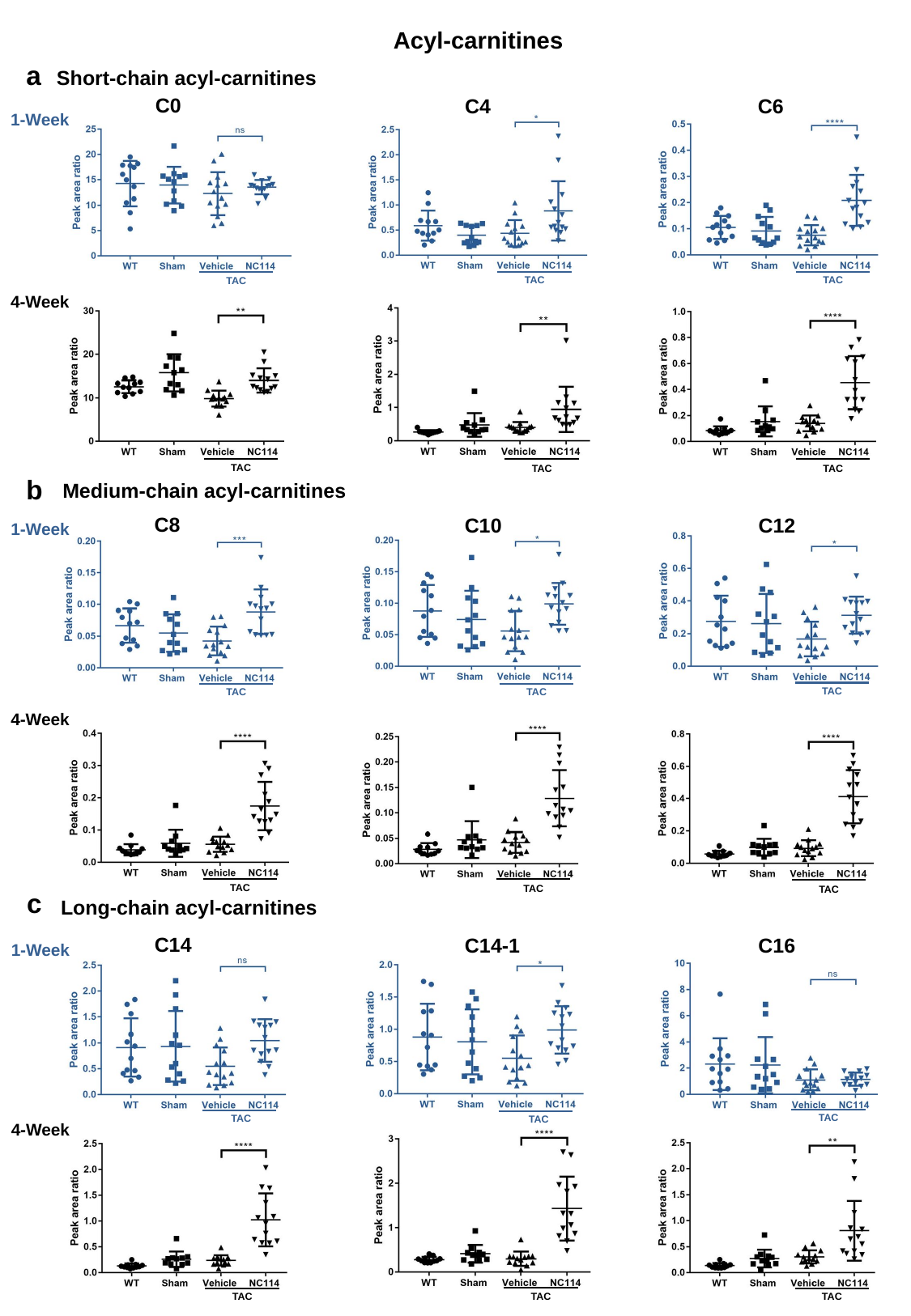

Acyl-carnitines
a
Short-chain acyl-carnitines
C0
C4
C6
1-Week
TAC
TAC
TAC
4-Week
TAC
TAC
TAC
b
Medium-chain acyl-carnitines
C8
C10
C12
1-Week
TAC
TAC
TAC
4-Week
TAC
TAC
TAC
c
Long-chain acyl-carnitines
C14
C14-1
C16
1-Week
TAC
TAC
TAC
4-Week
TAC
TAC
TAC

## Slide 27
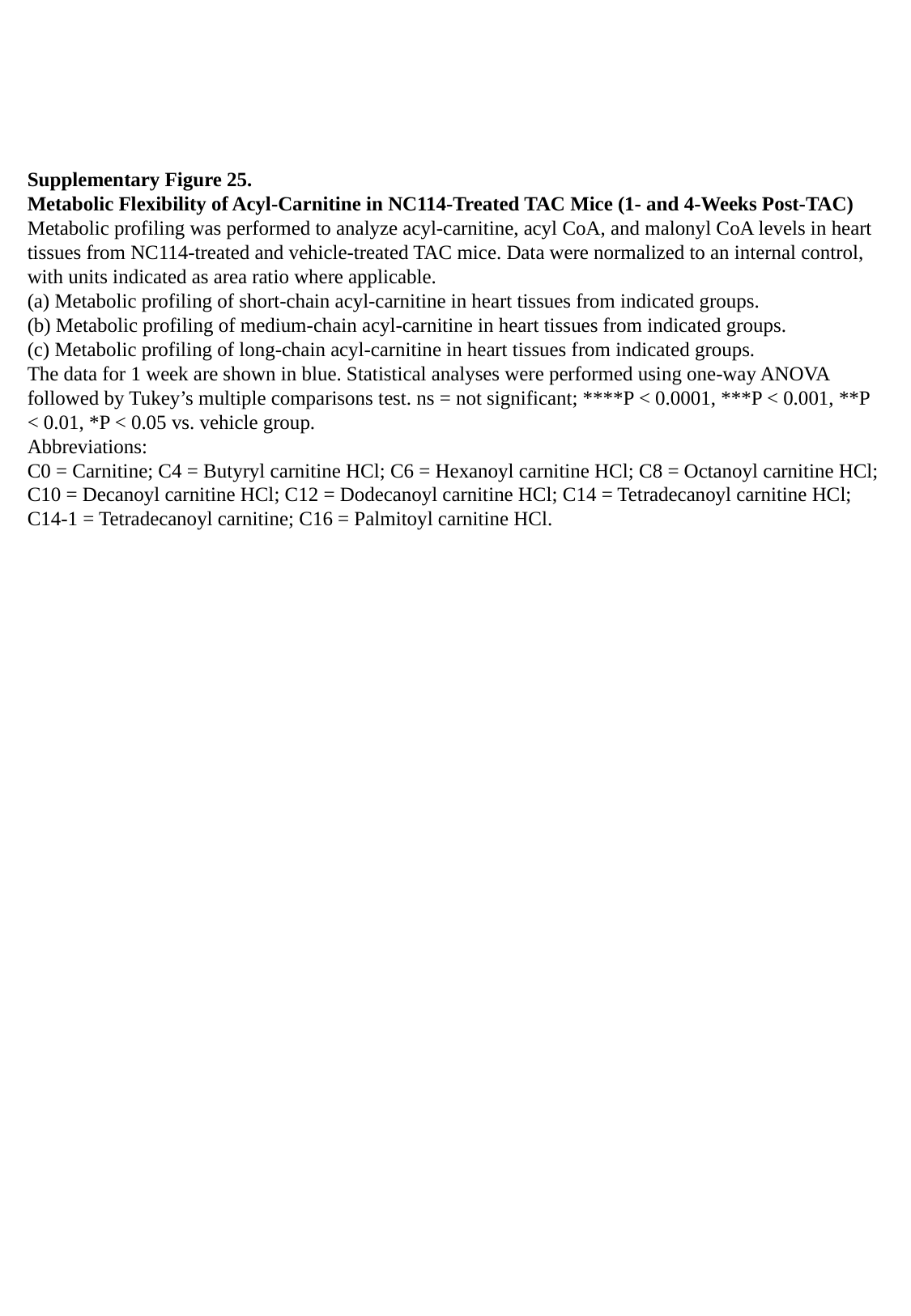

Supplementary Figure 25.
Metabolic Flexibility of Acyl-Carnitine in NC114-Treated TAC Mice (1- and 4-Weeks Post-TAC)
Metabolic profiling was performed to analyze acyl-carnitine, acyl CoA, and malonyl CoA levels in heart tissues from NC114-treated and vehicle-treated TAC mice. Data were normalized to an internal control, with units indicated as area ratio where applicable.
(a) Metabolic profiling of short-chain acyl-carnitine in heart tissues from indicated groups.
(b) Metabolic profiling of medium-chain acyl-carnitine in heart tissues from indicated groups.
(c) Metabolic profiling of long-chain acyl-carnitine in heart tissues from indicated groups.
The data for 1 week are shown in blue. Statistical analyses were performed using one-way ANOVA followed by Tukey’s multiple comparisons test. ns = not significant; ****P < 0.0001, ***P < 0.001, **P < 0.01, *P < 0.05 vs. vehicle group.
Abbreviations:
C0 = Carnitine; C4 = Butyryl carnitine HCl; C6 = Hexanoyl carnitine HCl; C8 = Octanoyl carnitine HCl; C10 = Decanoyl carnitine HCl; C12 = Dodecanoyl carnitine HCl; C14 = Tetradecanoyl carnitine HCl; C14-1 = Tetradecanoyl carnitine; C16 = Palmitoyl carnitine HCl.

## Slide 28
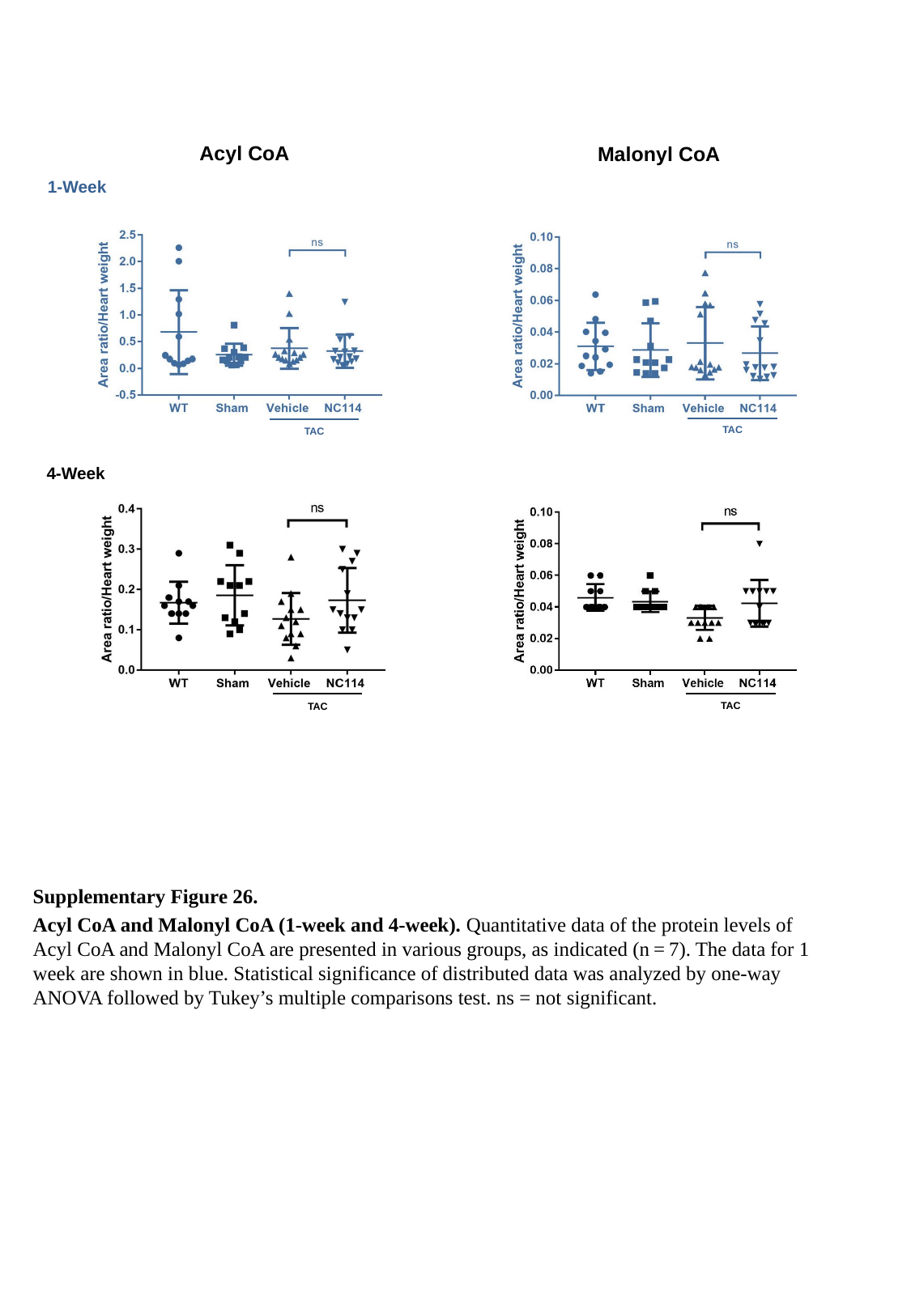

Acyl CoA
Malonyl CoA
1-Week
TAC
TAC
4-Week
TAC
TAC
Supplementary Figure 26.
Acyl CoA and Malonyl CoA (1-week and 4-week). Quantitative data of the protein levels of Acyl CoA and Malonyl CoA are presented in various groups, as indicated (n = 7). The data for 1 week are shown in blue. Statistical significance of distributed data was analyzed by one-way ANOVA followed by Tukey’s multiple comparisons test. ns = not significant.

## Slide 29
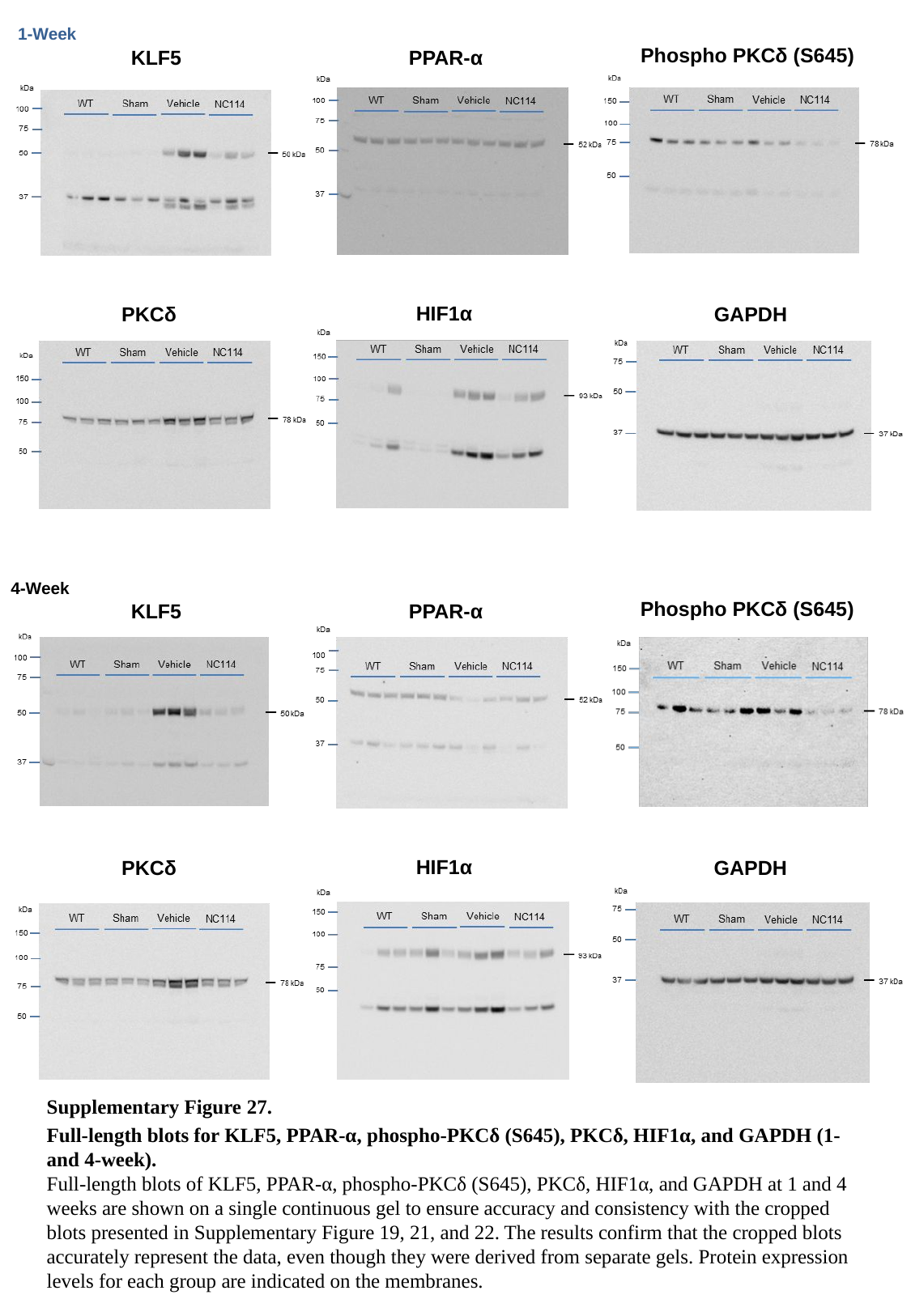

1-Week
Phospho PKCδ (S645)
KLF5
PPAR-α
HIF1α
PKCδ
GAPDH
4-Week
Phospho PKCδ (S645)
KLF5
PPAR-α
HIF1α
PKCδ
GAPDH
Supplementary Figure 27.
Full-length blots for KLF5, PPAR-α, phospho-PKCδ (S645), PKCδ, HIF1α, and GAPDH (1- and 4-week).
Full-length blots of KLF5, PPAR-α, phospho-PKCδ (S645), PKCδ, HIF1α, and GAPDH at 1 and 4 weeks are shown on a single continuous gel to ensure accuracy and consistency with the cropped blots presented in Supplementary Figure 19, 21, and 22. The results confirm that the cropped blots accurately represent the data, even though they were derived from separate gels. Protein expression levels for each group are indicated on the membranes.

## Slide 30
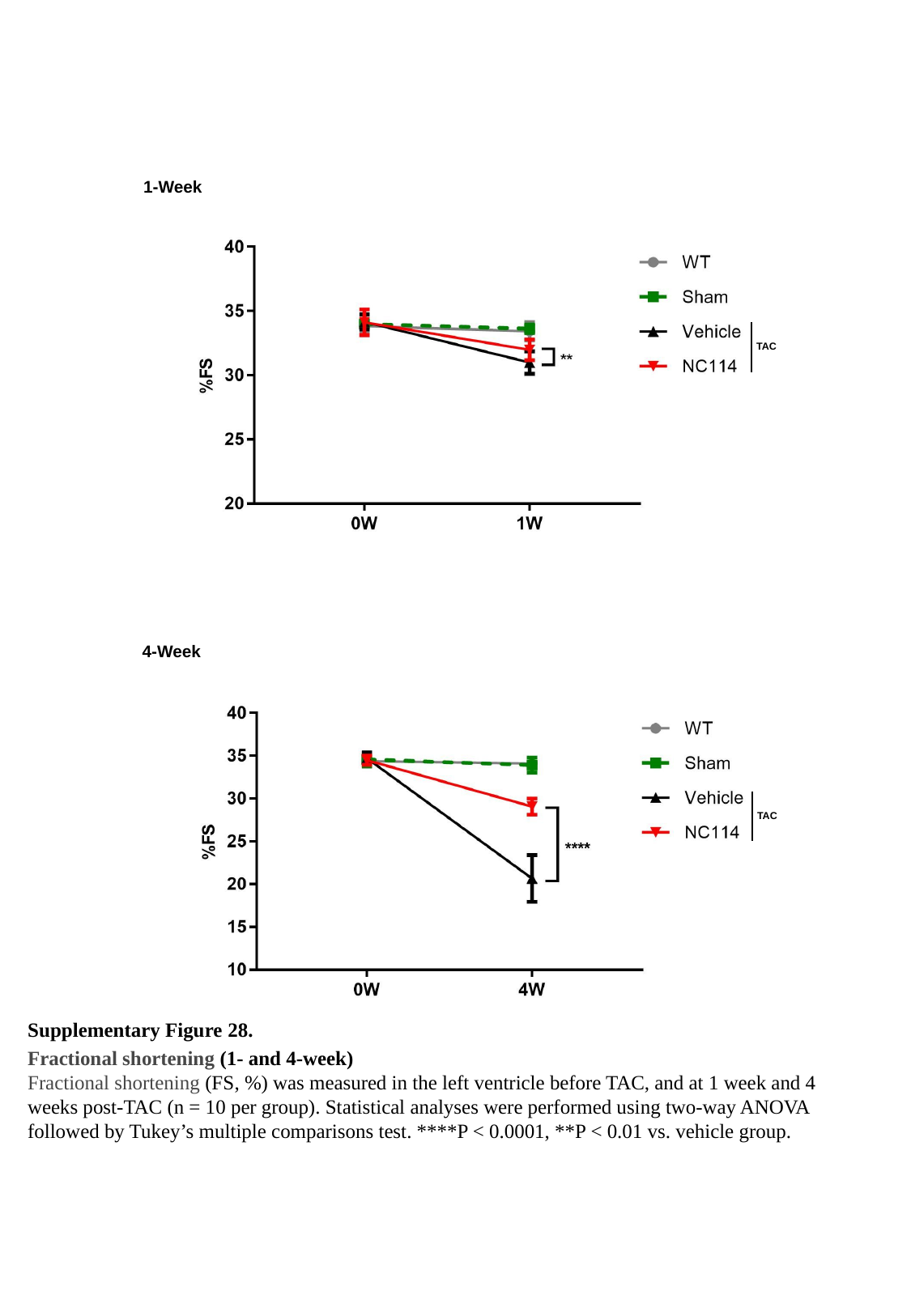

1-Week
TAC
4-Week
TAC
Supplementary Figure 28.
Fractional shortening (1- and 4-week)
Fractional shortening (FS, %) was measured in the left ventricle before TAC, and at 1 week and 4 weeks post-TAC (n = 10 per group). Statistical analyses were performed using two-way ANOVA followed by Tukey’s multiple comparisons test. ****P < 0.0001, **P < 0.01 vs. vehicle group.

## Slide 31
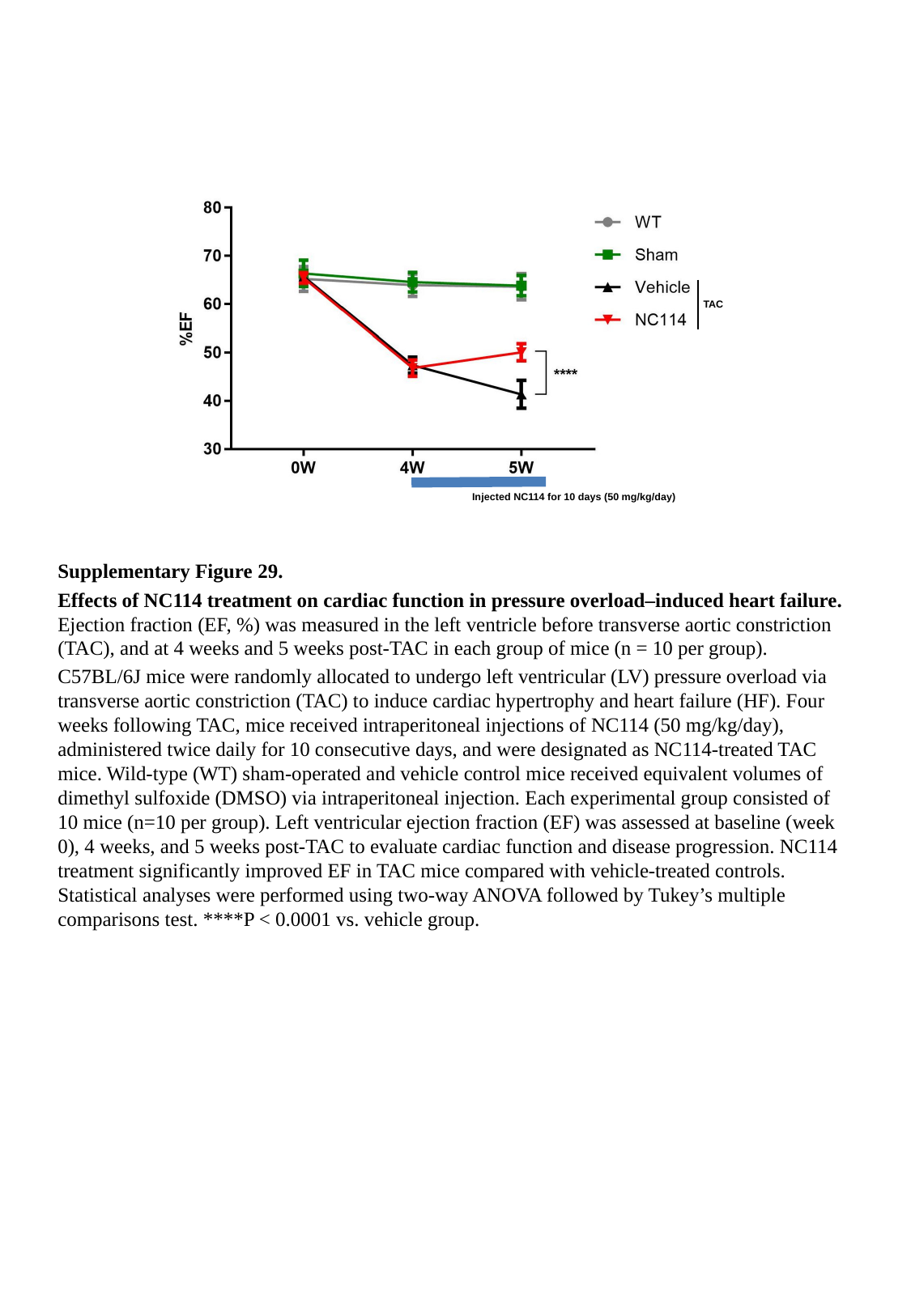

Injected NC114 for 10 days (50 mg/kg/day)
TAC
Supplementary Figure 29.
Effects of NC114 treatment on cardiac function in pressure overload–induced heart failure.
Ejection fraction (EF, %) was measured in the left ventricle before transverse aortic constriction (TAC), and at 4 weeks and 5 weeks post-TAC in each group of mice (n = 10 per group).
C57BL/6J mice were randomly allocated to undergo left ventricular (LV) pressure overload via transverse aortic constriction (TAC) to induce cardiac hypertrophy and heart failure (HF). Four weeks following TAC, mice received intraperitoneal injections of NC114 (50 mg/kg/day), administered twice daily for 10 consecutive days, and were designated as NC114-treated TAC mice. Wild-type (WT) sham-operated and vehicle control mice received equivalent volumes of dimethyl sulfoxide (DMSO) via intraperitoneal injection. Each experimental group consisted of 10 mice (n=10 per group). Left ventricular ejection fraction (EF) was assessed at baseline (week 0), 4 weeks, and 5 weeks post-TAC to evaluate cardiac function and disease progression. NC114 treatment significantly improved EF in TAC mice compared with vehicle-treated controls. Statistical analyses were performed using two-way ANOVA followed by Tukey’s multiple comparisons test. ****P < 0.0001 vs. vehicle group.

## Slide 32
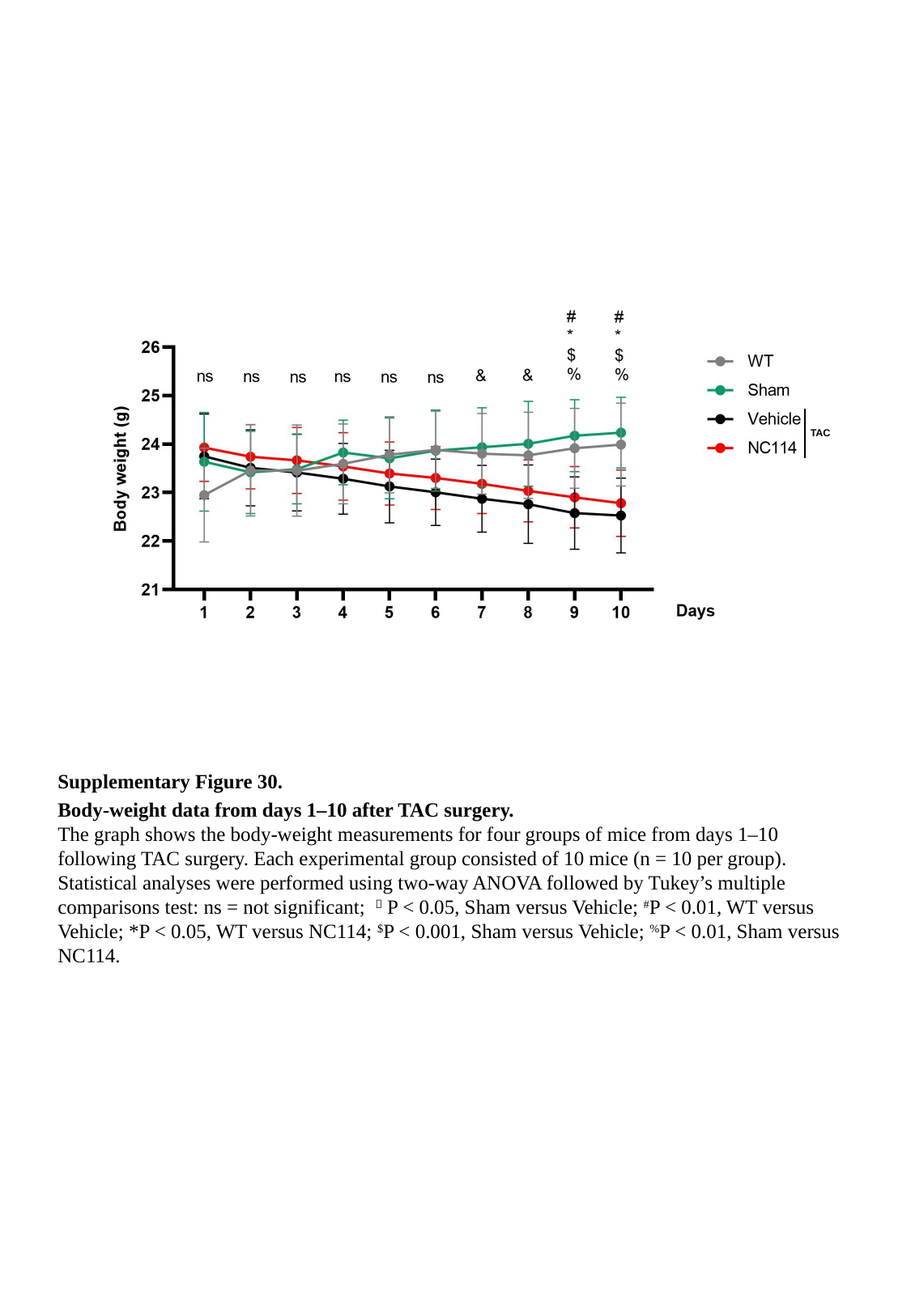

TAC
Supplementary Figure 30.
Body-weight data from days 1–10 after TAC surgery.
The graph shows the body-weight measurements for four groups of mice from days 1–10 following TAC surgery. Each experimental group consisted of 10 mice (n = 10 per group). Statistical analyses were performed using two-way ANOVA followed by Tukey’s multiple comparisons test: ns = not significant; ＆P < 0.05, Sham versus Vehicle; #P < 0.01, WT versus Vehicle; *P < 0.05, WT versus NC114; $P < 0.001, Sham versus Vehicle; %P < 0.01, Sham versus NC114.
