# Supplementary material for: Targeting the undruggable transcription factor, KLF5, with a peptidomimetic small molecule, NC114, attenuates pressure overload-induced cardiac remodeling and fibrosis
Source: Sci Rep. 2026 Jan 12;16:2367. doi: 10.1038/s41598-025-32155-y (PMC12816732; doi:10.1038/s41598-025-32155-y)
Supplement: Supplementary file 2 — Supplementary Information 2. [file 41598_2025_32155_MOESM2_ESM.pptx]

## Slide 1
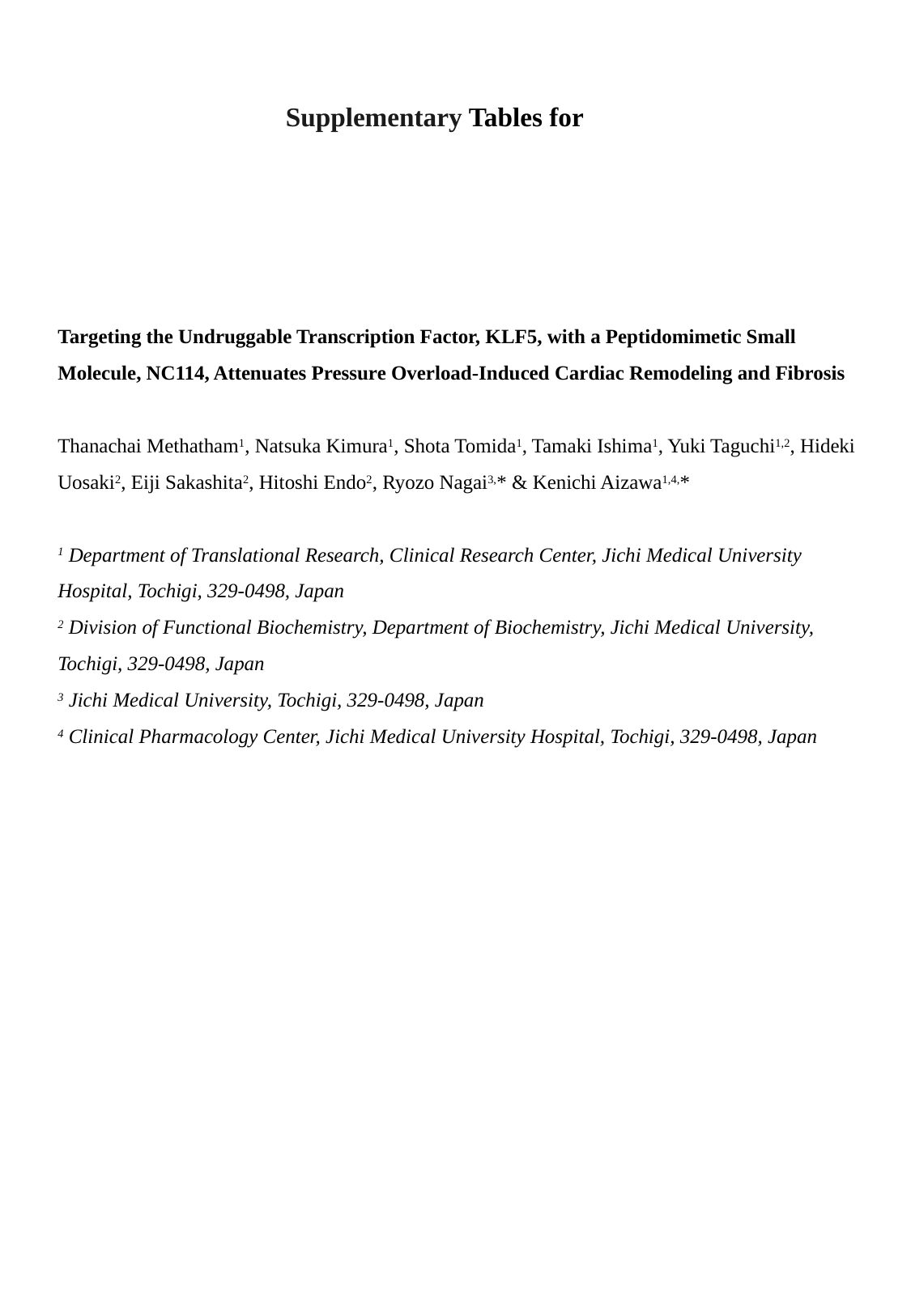

Supplementary Tables for
# Targeting the Undruggable Transcription Factor, KLF5, with a Peptidomimetic Small Molecule, NC114, Attenuates Pressure Overload-Induced Cardiac Remodeling and Fibrosis Thanachai Methatham1, Natsuka Kimura1, Shota Tomida1, Tamaki Ishima1, Yuki Taguchi1,2, Hideki Uosaki2, Eiji Sakashita2, Hitoshi Endo2, Ryozo Nagai3,* & Kenichi Aizawa1,4,*   1 Department of Translational Research, Clinical Research Center, Jichi Medical University Hospital, Tochigi, 329-0498, Japan 2 Division of Functional Biochemistry, Department of Biochemistry, Jichi Medical University, Tochigi, 329-0498, Japan 3 Jichi Medical University, Tochigi, 329-0498, Japan 4 Clinical Pharmacology Center, Jichi Medical University Hospital, Tochigi, 329-0498, Japan

## Slide 2
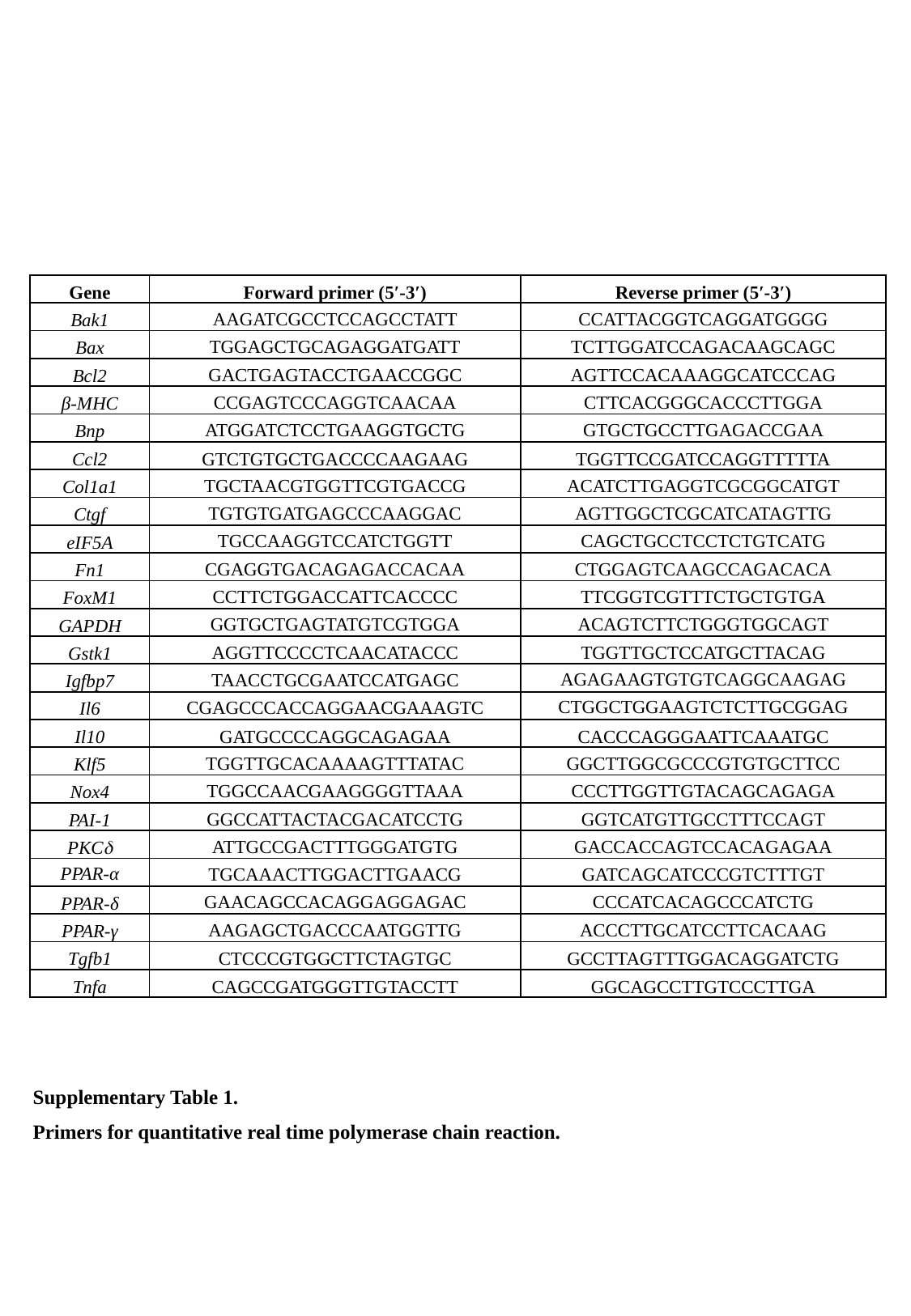

| Gene | Forward primer (5′-3′) | Reverse primer (5′-3′) |
| --- | --- | --- |
| Bak1 | AAGATCGCCTCCAGCCTATT | CCATTACGGTCAGGATGGGG |
| Bax | TGGAGCTGCAGAGGATGATT | TCTTGGATCCAGACAAGCAGC |
| Bcl2 | GACTGAGTACCTGAACCGGC | AGTTCCACAAAGGCATCCCAG |
| β-MHC | CCGAGTCCCAGGTCAACAA | CTTCACGGGCACCCTTGGA |
| Bnp | ATGGATCTCCTGAAGGTGCTG | GTGCTGCCTTGAGACCGAA |
| Ccl2 | GTCTGTGCTGACCCCAAGAAG | TGGTTCCGATCCAGGTTTTTA |
| Col1a1 | TGCTAACGTGGTTCGTGACCG | ACATCTTGAGGTCGCGGCATGT |
| Ctgf | TGTGTGATGAGCCCAAGGAC | AGTTGGCTCGCATCATAGTTG |
| eIF5A | TGCCAAGGTCCATCTGGTT | CAGCTGCCTCCTCTGTCATG |
| Fn1 | CGAGGTGACAGAGACCACAA | CTGGAGTCAAGCCAGACACA |
| FoxM1 | CCTTCTGGACCATTCACCCC | TTCGGTCGTTTCTGCTGTGA |
| GAPDH | GGTGCTGAGTATGTCGTGGA | ACAGTCTTCTGGGTGGCAGT |
| Gstk1 | AGGTTCCCCTCAACATACCC | TGGTTGCTCCATGCTTACAG |
| Igfbp7 | TAACCTGCGAATCCATGAGC | AGAGAAGTGTGTCAGGCAAGAG |
| Il6 | CGAGCCCACCAGGAACGAAAGTC | CTGGCTGGAAGTCTCTTGCGGAG |
| Il10 | GATGCCCCAGGCAGAGAA | CACCCAGGGAATTCAAATGC |
| Klf5 | TGGTTGCACAAAAGTTTATAC | GGCTTGGCGCCCGTGTGCTTCC |
| Nox4 | TGGCCAACGAAGGGGTTAAA | CCCTTGGTTGTACAGCAGAGA |
| PAI-1 | GGCCATTACTACGACATCCTG | GGTCATGTTGCCTTTCCAGT |
| PKCδ | ATTGCCGACTTTGGGATGTG | GACCACCAGTCCACAGAGAA |
| PPAR-α | TGCAAACTTGGACTTGAACG | GATCAGCATCCCGTCTTTGT |
| PPAR-δ | GAACAGCCACAGGAGGAGAC | CCCATCACAGCCCATCTG |
| PPAR-γ | AAGAGCTGACCCAATGGTTG | ACCCTTGCATCCTTCACAAG |
| Tgfb1 | CTCCCGTGGCTTCTAGTGC | GCCTTAGTTTGGACAGGATCTG |
| Tnfa | CAGCCGATGGGTTGTACCTT | GGCAGCCTTGTCCCTTGA |
Supplementary Table 1.
Primers for quantitative real time polymerase chain reaction.

## Slide 3
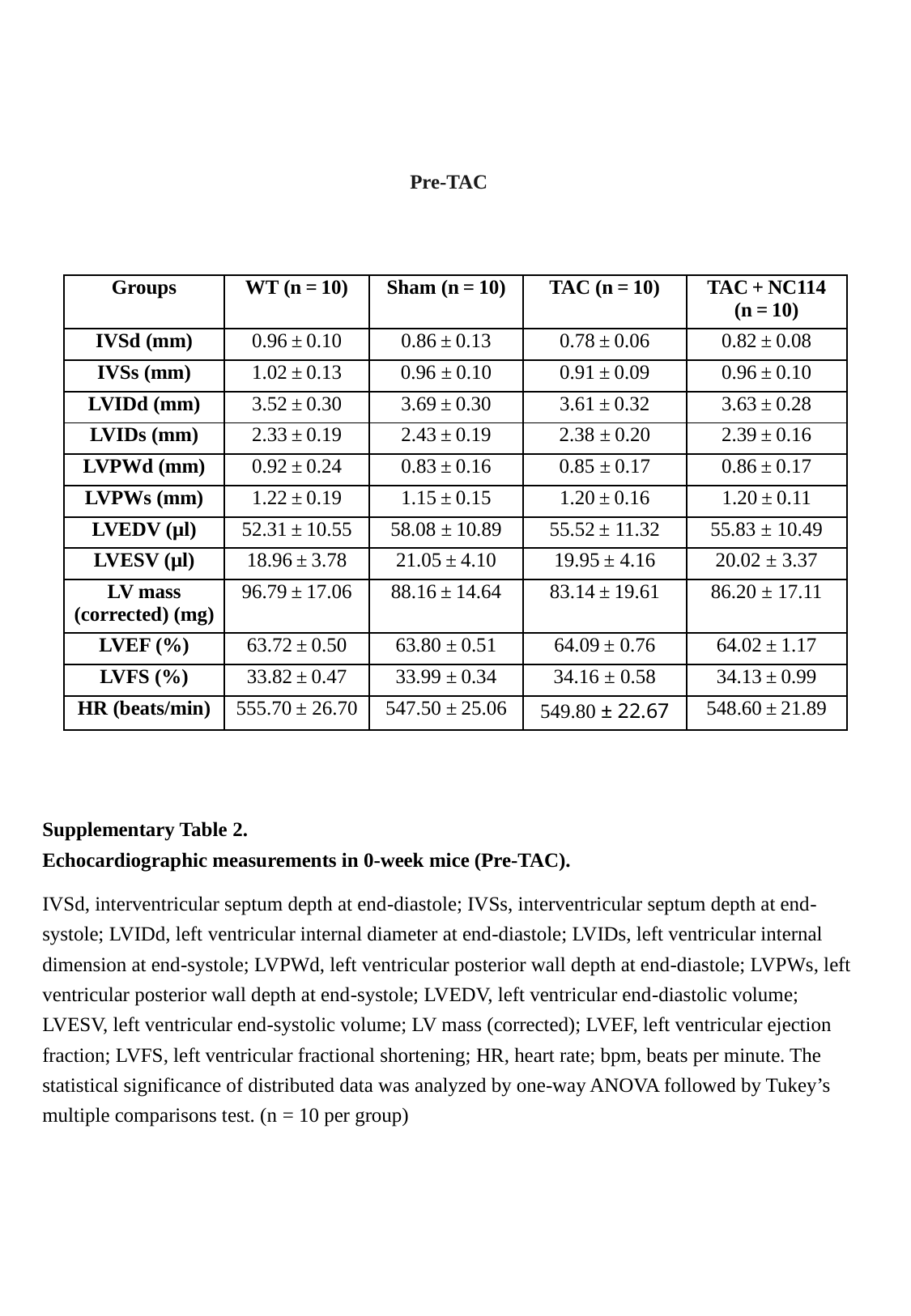

Pre-TAC
| Groups | WT (n = 10) | Sham (n = 10) | TAC (n = 10) | TAC + NC114 (n = 10) |
| --- | --- | --- | --- | --- |
| IVSd (mm) | 0.96 ± 0.10 | 0.86 ± 0.13 | 0.78 ± 0.06 | 0.82 ± 0.08 |
| IVSs (mm) | 1.02 ± 0.13 | 0.96 ± 0.10 | 0.91 ± 0.09 | 0.96 ± 0.10 |
| LVIDd (mm) | 3.52 ± 0.30 | 3.69 ± 0.30 | 3.61 ± 0.32 | 3.63 ± 0.28 |
| LVIDs (mm) | 2.33 ± 0.19 | 2.43 ± 0.19 | 2.38 ± 0.20 | 2.39 ± 0.16 |
| LVPWd (mm) | 0.92 ± 0.24 | 0.83 ± 0.16 | 0.85 ± 0.17 | 0.86 ± 0.17 |
| LVPWs (mm) | 1.22 ± 0.19 | 1.15 ± 0.15 | 1.20 ± 0.16 | 1.20 ± 0.11 |
| LVEDV (μl) | 52.31 ± 10.55 | 58.08 ± 10.89 | 55.52 ± 11.32 | 55.83 ± 10.49 |
| LVESV (μl) | 18.96 ± 3.78 | 21.05 ± 4.10 | 19.95 ± 4.16 | 20.02 ± 3.37 |
| LV mass (corrected) (mg) | 96.79 ± 17.06 | 88.16 ± 14.64 | 83.14 ± 19.61 | 86.20 ± 17.11 |
| LVEF (%) | 63.72 ± 0.50 | 63.80 ± 0.51 | 64.09 ± 0.76 | 64.02 ± 1.17 |
| LVFS (%) | 33.82 ± 0.47 | 33.99 ± 0.34 | 34.16 ± 0.58 | 34.13 ± 0.99 |
| HR (beats/min) | 555.70 ± 26.70 | 547.50 ± 25.06 | 549.80 ± 22.67 | 548.60 ± 21.89 |
Supplementary Table 2.
Echocardiographic measurements in 0-week mice (Pre-TAC).
IVSd, interventricular septum depth at end-diastole; IVSs, interventricular septum depth at end-systole; LVIDd, left ventricular internal diameter at end-diastole; LVIDs, left ventricular internal dimension at end-systole; LVPWd, left ventricular posterior wall depth at end-diastole; LVPWs, left ventricular posterior wall depth at end-systole; LVEDV, left ventricular end-diastolic volume; LVESV, left ventricular end-systolic volume; LV mass (corrected); LVEF, left ventricular ejection fraction; LVFS, left ventricular fractional shortening; HR, heart rate; bpm, beats per minute. The statistical significance of distributed data was analyzed by one-way ANOVA followed by Tukey’s multiple comparisons test. (n = 10 per group)

## Slide 4
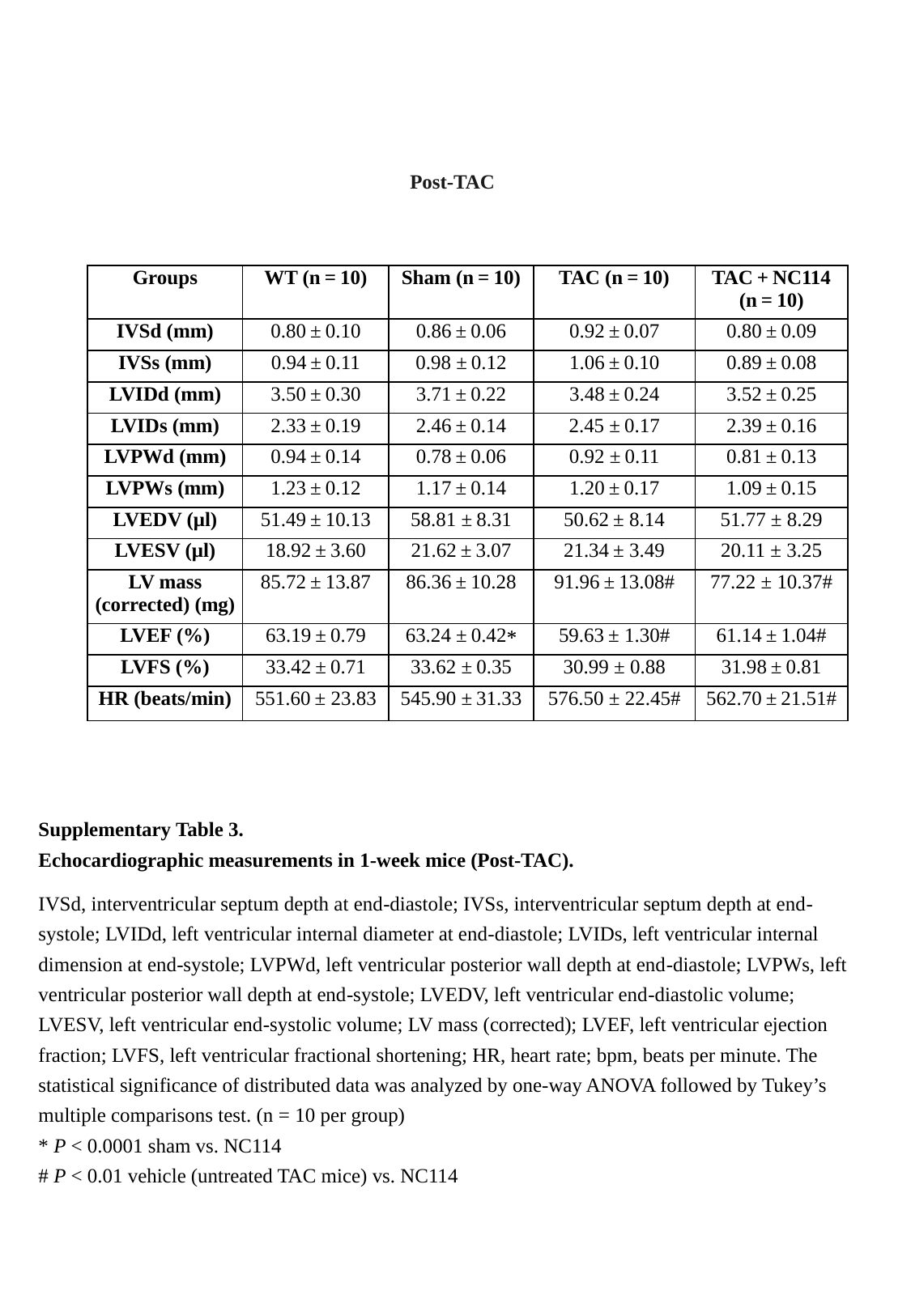

Post-TAC
| Groups | WT (n = 10) | Sham (n = 10) | TAC (n = 10) | TAC + NC114 (n = 10) |
| --- | --- | --- | --- | --- |
| IVSd (mm) | 0.80 ± 0.10 | 0.86 ± 0.06 | 0.92 ± 0.07 | 0.80 ± 0.09 |
| IVSs (mm) | 0.94 ± 0.11 | 0.98 ± 0.12 | 1.06 ± 0.10 | 0.89 ± 0.08 |
| LVIDd (mm) | 3.50 ± 0.30 | 3.71 ± 0.22 | 3.48 ± 0.24 | 3.52 ± 0.25 |
| LVIDs (mm) | 2.33 ± 0.19 | 2.46 ± 0.14 | 2.45 ± 0.17 | 2.39 ± 0.16 |
| LVPWd (mm) | 0.94 ± 0.14 | 0.78 ± 0.06 | 0.92 ± 0.11 | 0.81 ± 0.13 |
| LVPWs (mm) | 1.23 ± 0.12 | 1.17 ± 0.14 | 1.20 ± 0.17 | 1.09 ± 0.15 |
| LVEDV (μl) | 51.49 ± 10.13 | 58.81 ± 8.31 | 50.62 ± 8.14 | 51.77 ± 8.29 |
| LVESV (μl) | 18.92 ± 3.60 | 21.62 ± 3.07 | 21.34 ± 3.49 | 20.11 ± 3.25 |
| LV mass (corrected) (mg) | 85.72 ± 13.87 | 86.36 ± 10.28 | 91.96 ± 13.08# | 77.22 ± 10.37# |
| LVEF (%) | 63.19 ± 0.79 | 63.24 ± 0.42⁎ | 59.63 ± 1.30# | 61.14 ± 1.04# |
| LVFS (%) | 33.42 ± 0.71 | 33.62 ± 0.35 | 30.99 ± 0.88 | 31.98 ± 0.81 |
| HR (beats/min) | 551.60 ± 23.83 | 545.90 ± 31.33 | 576.50 ± 22.45# | 562.70 ± 21.51# |
Supplementary Table 3.
Echocardiographic measurements in 1-week mice (Post-TAC).
IVSd, interventricular septum depth at end-diastole; IVSs, interventricular septum depth at end-systole; LVIDd, left ventricular internal diameter at end-diastole; LVIDs, left ventricular internal dimension at end-systole; LVPWd, left ventricular posterior wall depth at end-diastole; LVPWs, left ventricular posterior wall depth at end-systole; LVEDV, left ventricular end-diastolic volume; LVESV, left ventricular end-systolic volume; LV mass (corrected); LVEF, left ventricular ejection fraction; LVFS, left ventricular fractional shortening; HR, heart rate; bpm, beats per minute. The statistical significance of distributed data was analyzed by one-way ANOVA followed by Tukey’s multiple comparisons test. (n = 10 per group)
* P < 0.0001 sham vs. NC114
# P < 0.01 vehicle (untreated TAC mice) vs. NC114

## Slide 5
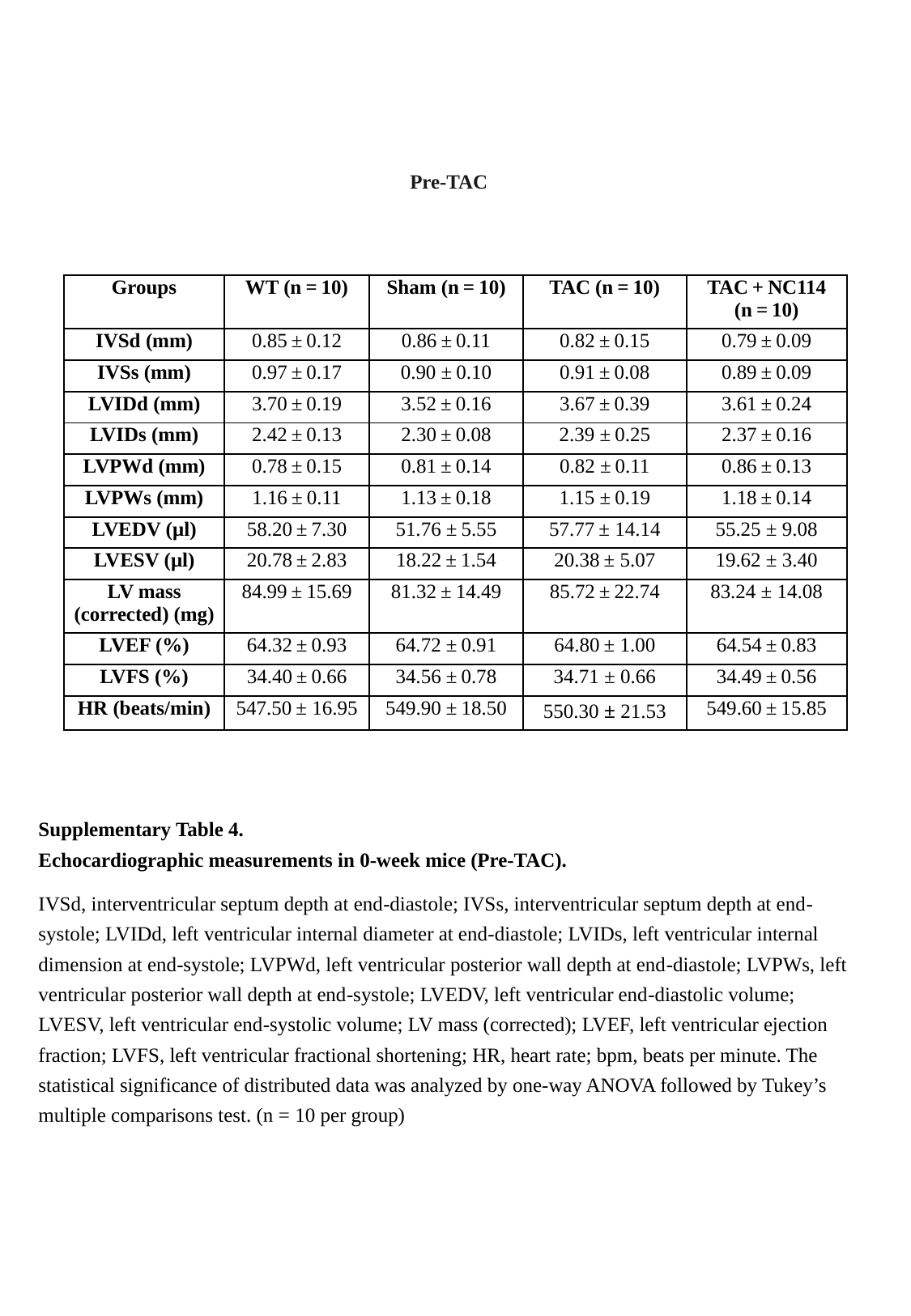

Pre-TAC
| Groups | WT (n = 10) | Sham (n = 10) | TAC (n = 10) | TAC + NC114 (n = 10) |
| --- | --- | --- | --- | --- |
| IVSd (mm) | 0.85 ± 0.12 | 0.86 ± 0.11 | 0.82 ± 0.15 | 0.79 ± 0.09 |
| IVSs (mm) | 0.97 ± 0.17 | 0.90 ± 0.10 | 0.91 ± 0.08 | 0.89 ± 0.09 |
| LVIDd (mm) | 3.70 ± 0.19 | 3.52 ± 0.16 | 3.67 ± 0.39 | 3.61 ± 0.24 |
| LVIDs (mm) | 2.42 ± 0.13 | 2.30 ± 0.08 | 2.39 ± 0.25 | 2.37 ± 0.16 |
| LVPWd (mm) | 0.78 ± 0.15 | 0.81 ± 0.14 | 0.82 ± 0.11 | 0.86 ± 0.13 |
| LVPWs (mm) | 1.16 ± 0.11 | 1.13 ± 0.18 | 1.15 ± 0.19 | 1.18 ± 0.14 |
| LVEDV (μl) | 58.20 ± 7.30 | 51.76 ± 5.55 | 57.77 ± 14.14 | 55.25 ± 9.08 |
| LVESV (μl) | 20.78 ± 2.83 | 18.22 ± 1.54 | 20.38 ± 5.07 | 19.62 ± 3.40 |
| LV mass (corrected) (mg) | 84.99 ± 15.69 | 81.32 ± 14.49 | 85.72 ± 22.74 | 83.24 ± 14.08 |
| LVEF (%) | 64.32 ± 0.93 | 64.72 ± 0.91 | 64.80 ± 1.00 | 64.54 ± 0.83 |
| LVFS (%) | 34.40 ± 0.66 | 34.56 ± 0.78 | 34.71 ± 0.66 | 34.49 ± 0.56 |
| HR (beats/min) | 547.50 ± 16.95 | 549.90 ± 18.50 | 550.30 ± 21.53 | 549.60 ± 15.85 |
Supplementary Table 4.
Echocardiographic measurements in 0-week mice (Pre-TAC).
IVSd, interventricular septum depth at end-diastole; IVSs, interventricular septum depth at end-systole; LVIDd, left ventricular internal diameter at end-diastole; LVIDs, left ventricular internal dimension at end-systole; LVPWd, left ventricular posterior wall depth at end-diastole; LVPWs, left ventricular posterior wall depth at end-systole; LVEDV, left ventricular end-diastolic volume; LVESV, left ventricular end-systolic volume; LV mass (corrected); LVEF, left ventricular ejection fraction; LVFS, left ventricular fractional shortening; HR, heart rate; bpm, beats per minute. The statistical significance of distributed data was analyzed by one-way ANOVA followed by Tukey’s multiple comparisons test. (n = 10 per group)

## Slide 6
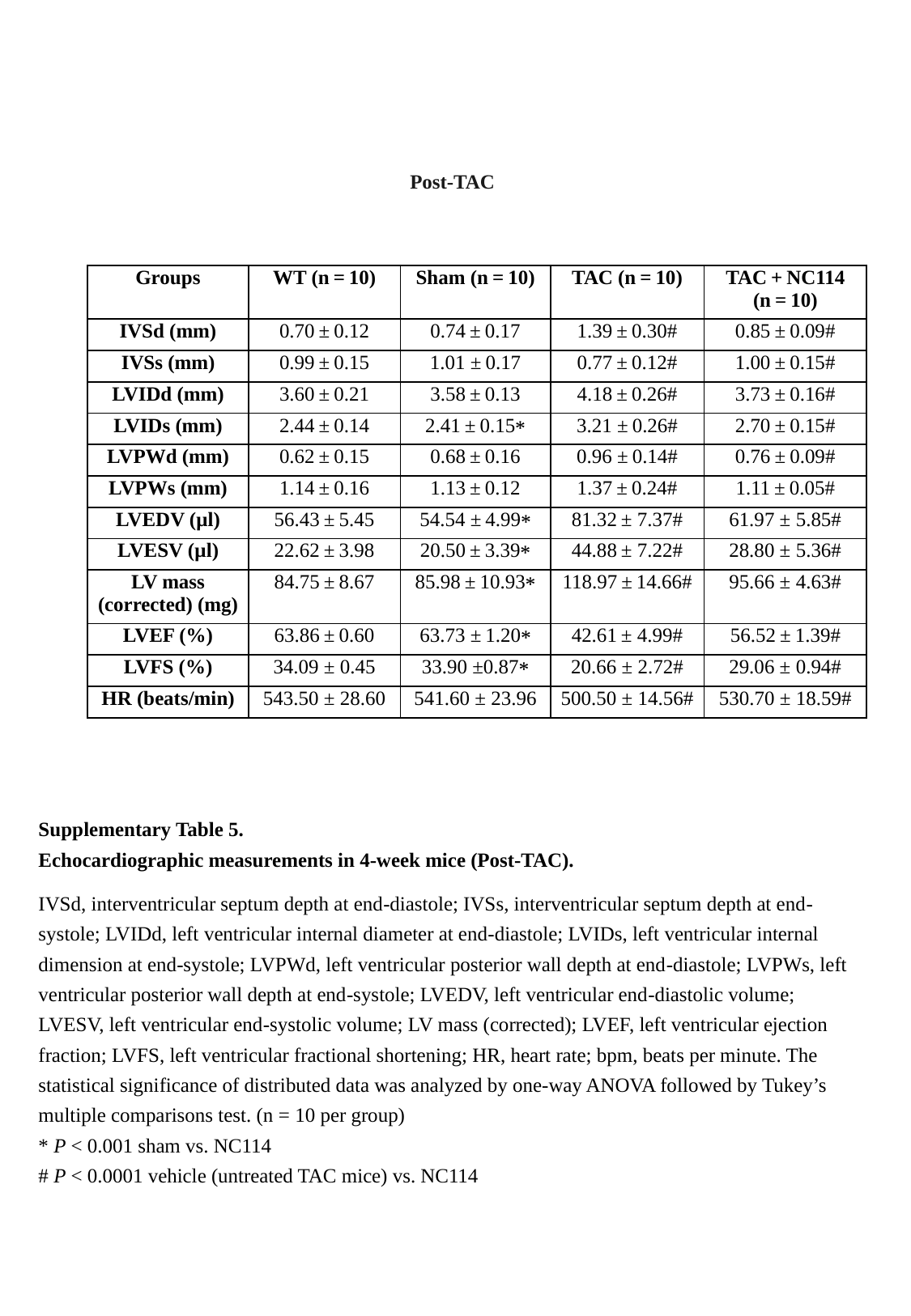

Post-TAC
| Groups | WT (n = 10) | Sham (n = 10) | TAC (n = 10) | TAC + NC114 (n = 10) |
| --- | --- | --- | --- | --- |
| IVSd (mm) | 0.70 ± 0.12 | 0.74 ± 0.17 | 1.39 ± 0.30# | 0.85 ± 0.09# |
| IVSs (mm) | 0.99 ± 0.15 | 1.01 ± 0.17 | 0.77 ± 0.12# | 1.00 ± 0.15# |
| LVIDd (mm) | 3.60 ± 0.21 | 3.58 ± 0.13 | 4.18 ± 0.26# | 3.73 ± 0.16# |
| LVIDs (mm) | 2.44 ± 0.14 | 2.41 ± 0.15⁎ | 3.21 ± 0.26# | 2.70 ± 0.15# |
| LVPWd (mm) | 0.62 ± 0.15 | 0.68 ± 0.16 | 0.96 ± 0.14# | 0.76 ± 0.09# |
| LVPWs (mm) | 1.14 ± 0.16 | 1.13 ± 0.12 | 1.37 ± 0.24# | 1.11 ± 0.05# |
| LVEDV (μl) | 56.43 ± 5.45 | 54.54 ± 4.99⁎ | 81.32 ± 7.37# | 61.97 ± 5.85# |
| LVESV (μl) | 22.62 ± 3.98 | 20.50 ± 3.39⁎ | 44.88 ± 7.22# | 28.80 ± 5.36# |
| LV mass (corrected) (mg) | 84.75 ± 8.67 | 85.98 ± 10.93⁎ | 118.97 ± 14.66# | 95.66 ± 4.63# |
| LVEF (%) | 63.86 ± 0.60 | 63.73 ± 1.20⁎ | 42.61 ± 4.99# | 56.52 ± 1.39# |
| LVFS (%) | 34.09 ± 0.45 | 33.90 ±0.87⁎ | 20.66 ± 2.72# | 29.06 ± 0.94# |
| HR (beats/min) | 543.50 ± 28.60 | 541.60 ± 23.96 | 500.50 ± 14.56# | 530.70 ± 18.59# |
Supplementary Table 5.
Echocardiographic measurements in 4-week mice (Post-TAC).
IVSd, interventricular septum depth at end-diastole; IVSs, interventricular septum depth at end-systole; LVIDd, left ventricular internal diameter at end-diastole; LVIDs, left ventricular internal dimension at end-systole; LVPWd, left ventricular posterior wall depth at end-diastole; LVPWs, left ventricular posterior wall depth at end-systole; LVEDV, left ventricular end-diastolic volume; LVESV, left ventricular end-systolic volume; LV mass (corrected); LVEF, left ventricular ejection fraction; LVFS, left ventricular fractional shortening; HR, heart rate; bpm, beats per minute. The statistical significance of distributed data was analyzed by one-way ANOVA followed by Tukey’s multiple comparisons test. (n = 10 per group)
* P < 0.001 sham vs. NC114
# P < 0.0001 vehicle (untreated TAC mice) vs. NC114

## Slide 7
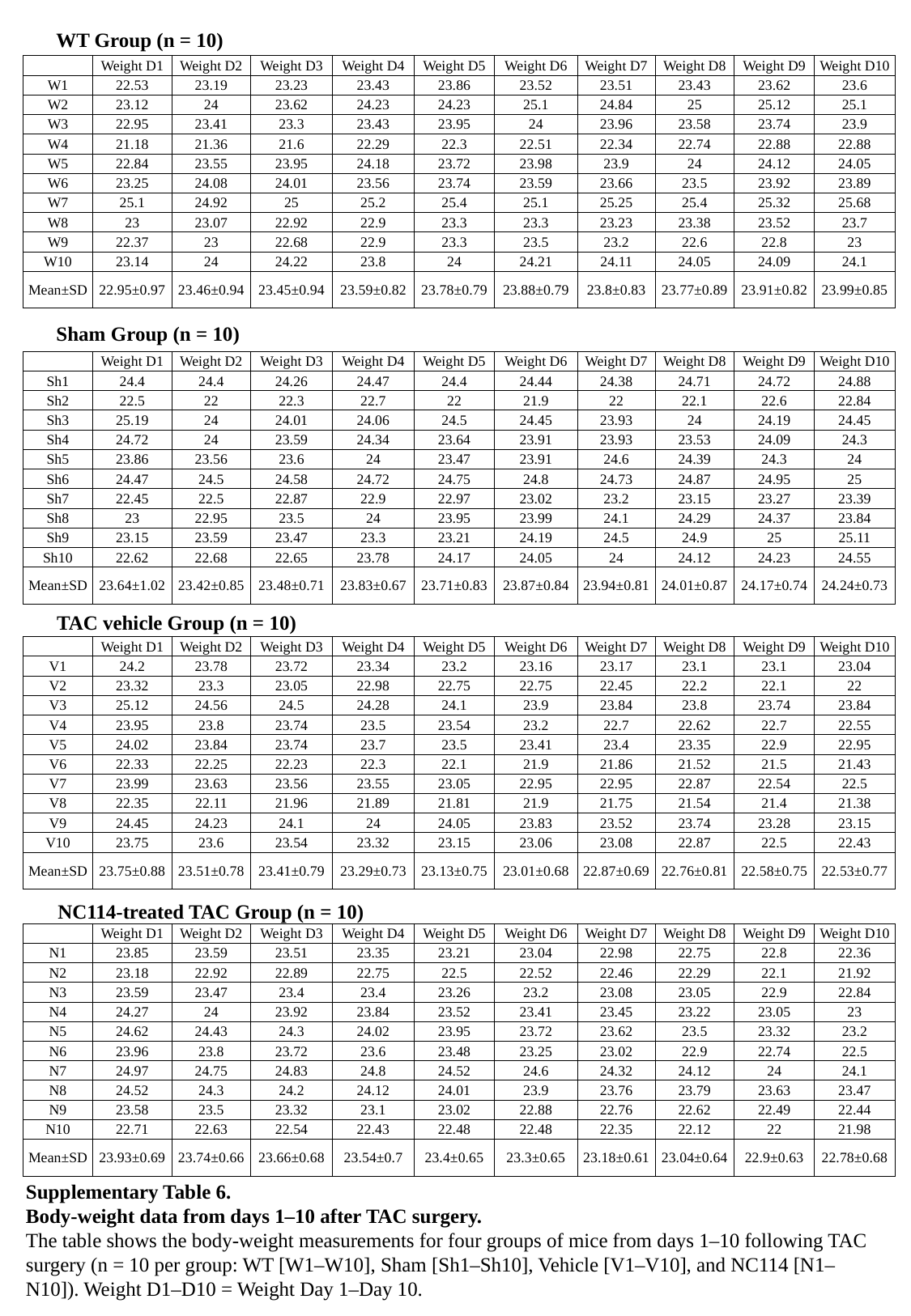

WT Group (n = 10)
| | Weight D1 | Weight D2 | Weight D3 | Weight D4 | Weight D5 | Weight D6 | Weight D7 | Weight D8 | Weight D9 | Weight D10 |
| --- | --- | --- | --- | --- | --- | --- | --- | --- | --- | --- |
| W1 | 22.53 | 23.19 | 23.23 | 23.43 | 23.86 | 23.52 | 23.51 | 23.43 | 23.62 | 23.6 |
| W2 | 23.12 | 24 | 23.62 | 24.23 | 24.23 | 25.1 | 24.84 | 25 | 25.12 | 25.1 |
| W3 | 22.95 | 23.41 | 23.3 | 23.43 | 23.95 | 24 | 23.96 | 23.58 | 23.74 | 23.9 |
| W4 | 21.18 | 21.36 | 21.6 | 22.29 | 22.3 | 22.51 | 22.34 | 22.74 | 22.88 | 22.88 |
| W5 | 22.84 | 23.55 | 23.95 | 24.18 | 23.72 | 23.98 | 23.9 | 24 | 24.12 | 24.05 |
| W6 | 23.25 | 24.08 | 24.01 | 23.56 | 23.74 | 23.59 | 23.66 | 23.5 | 23.92 | 23.89 |
| W7 | 25.1 | 24.92 | 25 | 25.2 | 25.4 | 25.1 | 25.25 | 25.4 | 25.32 | 25.68 |
| W8 | 23 | 23.07 | 22.92 | 22.9 | 23.3 | 23.3 | 23.23 | 23.38 | 23.52 | 23.7 |
| W9 | 22.37 | 23 | 22.68 | 22.9 | 23.3 | 23.5 | 23.2 | 22.6 | 22.8 | 23 |
| W10 | 23.14 | 24 | 24.22 | 23.8 | 24 | 24.21 | 24.11 | 24.05 | 24.09 | 24.1 |
| Mean±SD | 22.95±0.97 | 23.46±0.94 | 23.45±0.94 | 23.59±0.82 | 23.78±0.79 | 23.88±0.79 | 23.8±0.83 | 23.77±0.89 | 23.91±0.82 | 23.99±0.85 |
Sham Group (n = 10)
| | Weight D1 | Weight D2 | Weight D3 | Weight D4 | Weight D5 | Weight D6 | Weight D7 | Weight D8 | Weight D9 | Weight D10 |
| --- | --- | --- | --- | --- | --- | --- | --- | --- | --- | --- |
| Sh1 | 24.4 | 24.4 | 24.26 | 24.47 | 24.4 | 24.44 | 24.38 | 24.71 | 24.72 | 24.88 |
| Sh2 | 22.5 | 22 | 22.3 | 22.7 | 22 | 21.9 | 22 | 22.1 | 22.6 | 22.84 |
| Sh3 | 25.19 | 24 | 24.01 | 24.06 | 24.5 | 24.45 | 23.93 | 24 | 24.19 | 24.45 |
| Sh4 | 24.72 | 24 | 23.59 | 24.34 | 23.64 | 23.91 | 23.93 | 23.53 | 24.09 | 24.3 |
| Sh5 | 23.86 | 23.56 | 23.6 | 24 | 23.47 | 23.91 | 24.6 | 24.39 | 24.3 | 24 |
| Sh6 | 24.47 | 24.5 | 24.58 | 24.72 | 24.75 | 24.8 | 24.73 | 24.87 | 24.95 | 25 |
| Sh7 | 22.45 | 22.5 | 22.87 | 22.9 | 22.97 | 23.02 | 23.2 | 23.15 | 23.27 | 23.39 |
| Sh8 | 23 | 22.95 | 23.5 | 24 | 23.95 | 23.99 | 24.1 | 24.29 | 24.37 | 23.84 |
| Sh9 | 23.15 | 23.59 | 23.47 | 23.3 | 23.21 | 24.19 | 24.5 | 24.9 | 25 | 25.11 |
| Sh10 | 22.62 | 22.68 | 22.65 | 23.78 | 24.17 | 24.05 | 24 | 24.12 | 24.23 | 24.55 |
| Mean±SD | 23.64±1.02 | 23.42±0.85 | 23.48±0.71 | 23.83±0.67 | 23.71±0.83 | 23.87±0.84 | 23.94±0.81 | 24.01±0.87 | 24.17±0.74 | 24.24±0.73 |
TAC vehicle Group (n = 10)
| | Weight D1 | Weight D2 | Weight D3 | Weight D4 | Weight D5 | Weight D6 | Weight D7 | Weight D8 | Weight D9 | Weight D10 |
| --- | --- | --- | --- | --- | --- | --- | --- | --- | --- | --- |
| V1 | 24.2 | 23.78 | 23.72 | 23.34 | 23.2 | 23.16 | 23.17 | 23.1 | 23.1 | 23.04 |
| V2 | 23.32 | 23.3 | 23.05 | 22.98 | 22.75 | 22.75 | 22.45 | 22.2 | 22.1 | 22 |
| V3 | 25.12 | 24.56 | 24.5 | 24.28 | 24.1 | 23.9 | 23.84 | 23.8 | 23.74 | 23.84 |
| V4 | 23.95 | 23.8 | 23.74 | 23.5 | 23.54 | 23.2 | 22.7 | 22.62 | 22.7 | 22.55 |
| V5 | 24.02 | 23.84 | 23.74 | 23.7 | 23.5 | 23.41 | 23.4 | 23.35 | 22.9 | 22.95 |
| V6 | 22.33 | 22.25 | 22.23 | 22.3 | 22.1 | 21.9 | 21.86 | 21.52 | 21.5 | 21.43 |
| V7 | 23.99 | 23.63 | 23.56 | 23.55 | 23.05 | 22.95 | 22.95 | 22.87 | 22.54 | 22.5 |
| V8 | 22.35 | 22.11 | 21.96 | 21.89 | 21.81 | 21.9 | 21.75 | 21.54 | 21.4 | 21.38 |
| V9 | 24.45 | 24.23 | 24.1 | 24 | 24.05 | 23.83 | 23.52 | 23.74 | 23.28 | 23.15 |
| V10 | 23.75 | 23.6 | 23.54 | 23.32 | 23.15 | 23.06 | 23.08 | 22.87 | 22.5 | 22.43 |
| Mean±SD | 23.75±0.88 | 23.51±0.78 | 23.41±0.79 | 23.29±0.73 | 23.13±0.75 | 23.01±0.68 | 22.87±0.69 | 22.76±0.81 | 22.58±0.75 | 22.53±0.77 |
NC114-treated TAC Group (n = 10)
| | Weight D1 | Weight D2 | Weight D3 | Weight D4 | Weight D5 | Weight D6 | Weight D7 | Weight D8 | Weight D9 | Weight D10 |
| --- | --- | --- | --- | --- | --- | --- | --- | --- | --- | --- |
| N1 | 23.85 | 23.59 | 23.51 | 23.35 | 23.21 | 23.04 | 22.98 | 22.75 | 22.8 | 22.36 |
| N2 | 23.18 | 22.92 | 22.89 | 22.75 | 22.5 | 22.52 | 22.46 | 22.29 | 22.1 | 21.92 |
| N3 | 23.59 | 23.47 | 23.4 | 23.4 | 23.26 | 23.2 | 23.08 | 23.05 | 22.9 | 22.84 |
| N4 | 24.27 | 24 | 23.92 | 23.84 | 23.52 | 23.41 | 23.45 | 23.22 | 23.05 | 23 |
| N5 | 24.62 | 24.43 | 24.3 | 24.02 | 23.95 | 23.72 | 23.62 | 23.5 | 23.32 | 23.2 |
| N6 | 23.96 | 23.8 | 23.72 | 23.6 | 23.48 | 23.25 | 23.02 | 22.9 | 22.74 | 22.5 |
| N7 | 24.97 | 24.75 | 24.83 | 24.8 | 24.52 | 24.6 | 24.32 | 24.12 | 24 | 24.1 |
| N8 | 24.52 | 24.3 | 24.2 | 24.12 | 24.01 | 23.9 | 23.76 | 23.79 | 23.63 | 23.47 |
| N9 | 23.58 | 23.5 | 23.32 | 23.1 | 23.02 | 22.88 | 22.76 | 22.62 | 22.49 | 22.44 |
| N10 | 22.71 | 22.63 | 22.54 | 22.43 | 22.48 | 22.48 | 22.35 | 22.12 | 22 | 21.98 |
| Mean±SD | 23.93±0.69 | 23.74±0.66 | 23.66±0.68 | 23.54±0.7 | 23.4±0.65 | 23.3±0.65 | 23.18±0.61 | 23.04±0.64 | 22.9±0.63 | 22.78±0.68 |
Supplementary Table 6.
Body-weight data from days 1–10 after TAC surgery.
The table shows the body-weight measurements for four groups of mice from days 1–10 following TAC surgery (n = 10 per group: WT [W1–W10], Sham [Sh1–Sh10], Vehicle [V1–V10], and NC114 [N1–N10]). Weight D1–D10 = Weight Day 1–Day 10.
